# Iron-Catalyzed Cross-[2 + 2] Cycloaddition of Butadiene and α,ω-Dienes for Ductile and Chemically Recyclable Poly(oligocyclobutanes)

**DOI:** 10.1021/jacs.5c23398

**Published:** 2026-04-10

**Authors:** Cherish Nie, Sahana V. Sundar, Hang Zhang, Richard A. Register, Michael A. Webb, Rodney D. Priestley, Emily C. Davidson, Paul J. Chirik

**Affiliations:** † Department of Chemistry, 6740 Princeton University, Princeton, New Jersey 08544, United States; ‡ Department of Chemical and Biological Engineering, 6740 Princeton University, Princeton, New Jersey 08544, United States; § Princeton Materials Institute, Princeton University, Princeton, New Jersey 08544, United States

## Abstract

Poly­(divinyloligocyclobutanes) (pDVOCBs) are a distinct class of butadiene-derived, chemically recyclable polyolefins prepared from iron-catalyzed [2 + 2] cycloaddition followed by ruthenium-catalyzed ADMET polymerization. These polymers are highly crystalline, with high melting temperatures and unique rotator phases. The high degree of crystallinity in these materials not only leads to high stiffness over a broad temperature range but also results in brittle failure for polymers of modest molecular weights. Iron-catalyzed cross-[2 + 2] cycloaddition of butadiene and α,ω-dienes was accomplished, where the number of methylene units between the four-membered rings was systematically varied. Subsequent ADMET polymerization of the resulting telechelic oligomers provided a new class of chemically recyclable polyolefins that exhibit distinct thermomechanical properties. Specifically, methylene units induce changes in crystallinity that, in combination with the increased molecular weight, favored the formation of stable necks upon tensile deformation, with ∼10× enhanced ductility and improved toughness compared to pDVOCB. Through combined experimental and computational studies, the effects of methylene spacers on the crystal-to-rotator transition of the cyclobutane polymers were established. Ultimately, these methylene-modified cyclobutane polymers expand the range of properties and potential applications of this class of recyclable polyolefins.

## Graphical abstract

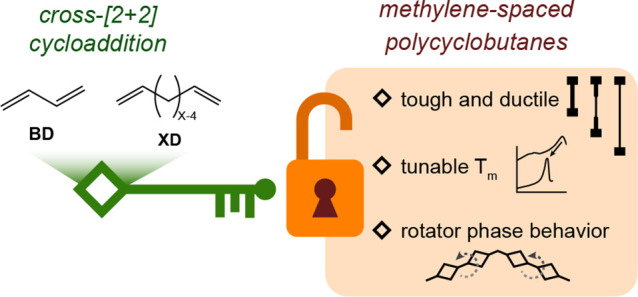

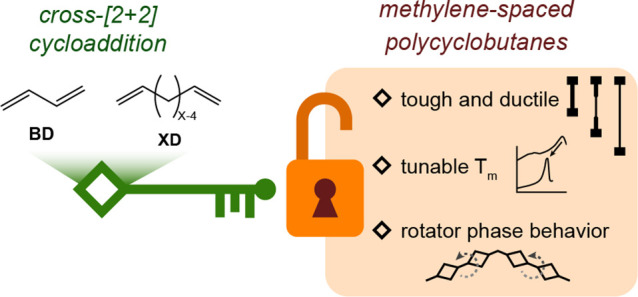

## Introduction

Polyolefins, specifically polyethylene (PE) and polypropylene (PP), constitute the majority of plastic resins and postconsumer waste and are derived from inexpensive and abundant feedstock monomers.^1,2^ Current strategies for mitigation of polyolefin waste include mechanical recycling, which results in lower-value materials, and incineration, which enables energy recovery but results in the loss of valuable hydrocarbon feedstocks with the generation of CO_2_.^3,4^ Chemical recycling back to monomer represents a more attractive approach to mitigating plastic accumulation; however, the high thermodynamic stability of PE and PP imposes substantial kinetic barriers to the regeneration of the starting alkenes.

The pursuit of more sustainable alternatives to traditional polyolefins has motivated the development of new polymers sourced from commodity olefins and designed with end-of-life considerations such as chemical recyclability.^5–7^ Examples of chemically recyclable polyolefins include those derived from cycloolefins, such as fused-ring cyclooctene-derived polymers, where ring strain is used to enable depolymerization back to monomer.^8,9^ However, these and other chemically recyclable polyolefins rely on specialized cyclic monomers to achieve depolymerization through ring-closing pathways.^8,10^

A new class of butadiene-derived polyolefins has been synthesized by iron-catalyzed [2 + 2] cycloaddition. The resulting (1,*n*′-divinyl)-oligocyclobutane (DVOCB) features a backbone of enchained 1,3-cyclobutane units consisting of a stereoirregular, equal mixture of *syn* and *anti* conformations. DVOCB is chemically recyclable, as re-exposure to the iron catalyst under vacuum returns free butadiene (Scheme 1A).^11^ DVOCB of varying oligomer lengths has been prepared and chain-extended by acyclic diene metathesis (ADMET) polymerization to yield tunable semicrystalline pDVOCB polymers with high thermal stability (*T*_d_ ∼ 365 °C), high melting temperatures (*T*_m_ > 240 °C), and modulus and tensile strengths comparable to those of isotactic polypropylene (iPP) and high-density polyethylene (HDPE).^12^ These materials, which are amenable to two-tiered chemical recycling, demonstrated that the introduction of enchained cyclobutanes into polymer backbones imparts increased rigidity to polymer chains without compromising thermal stability.^13–19^ Both DVOCB and pDVOCB exhibited increased crystallinity and *T*_m_ with longer sequences of 1,3-enchained cyclobutane rings in the backbone.

**Scheme 1:**
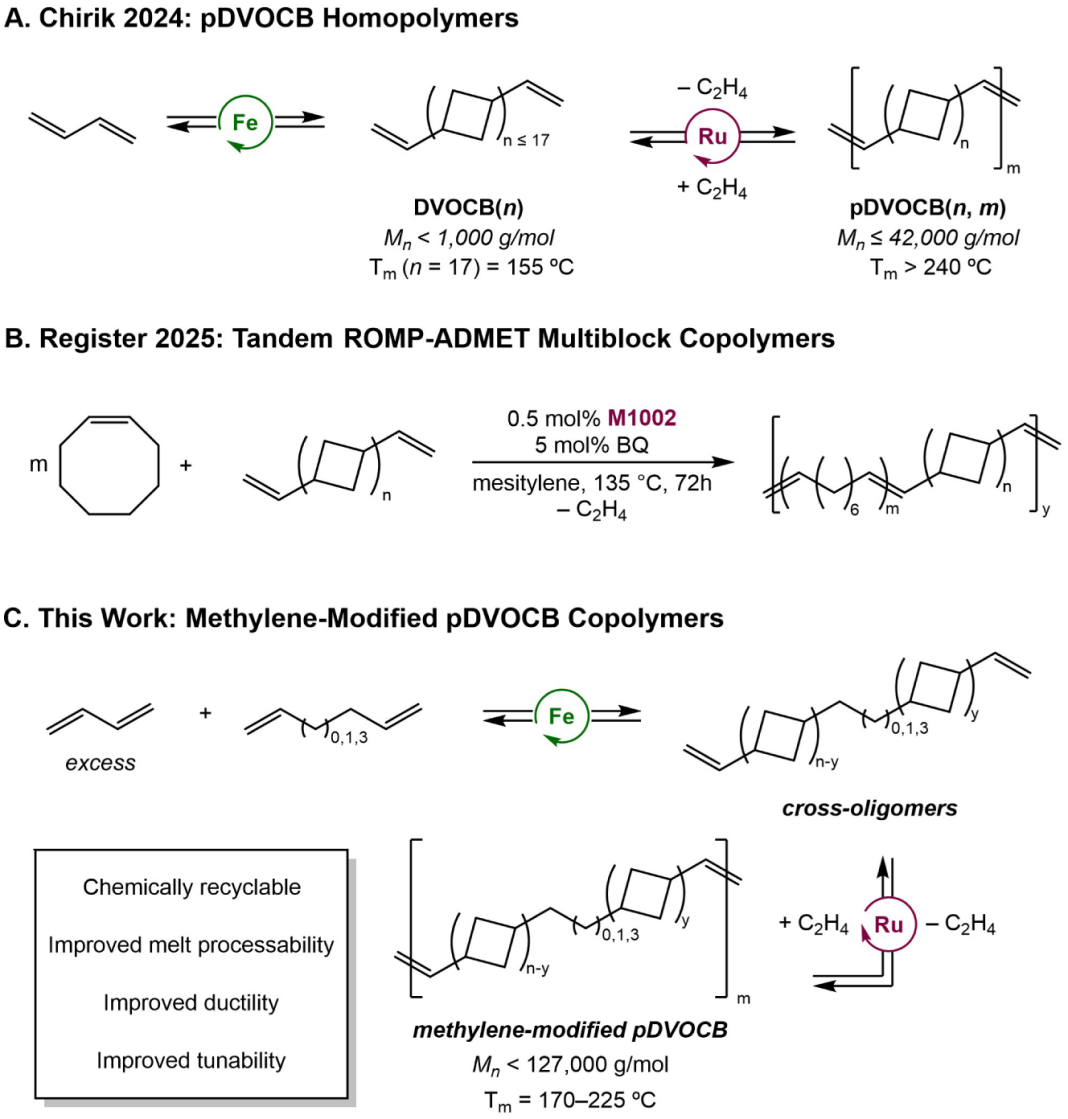
Incorporation of Methylene Spacers into Polycyclobutanes

The 1,3-enchainment of cyclobutyl groups also induces the formation of rotator phases,^20,21^ distinguished by semi-independent rotation of enchained rings in both DVOCB oligomers and pDVOCB polymers.^22^ Such rotationally disordered phases are well-studied in short *n*-alkanes^23–26^ but have rarely been observed in polymers.^27^ Despite this, rotator phases are frequently observed across DVOCB-derived oligomers and polymers, and modulation of these properties is anticipated to be critical for tailoring mechanical properties in these emerging materials. While the high degree of crystallinity and high *T*_m_ of pDVOCB preserve stiffness up to high temperatures, they also impart brittle behavior and necessitate higher temperature processing than typically employed for commodity polyolefins.^28^ To overcome these limitations, methylene spacers were inserted between cyclobutane units to preserve chemical recyclability while tuning the crystallinity and rotator phase interactions.

The addition of methylene spacers between monomer units has been shown to control crystallinity and flexibility in several polymer structures.^29–31^ Tandem ring-opening metathesis and acyclic diene metathesis (ROMP-ADMET) copolymerization of cyclooctene and DVOCB­(*n*) has proven to be a successful approach to access random multiblock copolymers with tunable crystallinity by modulating the ratio of flexible methylene segments and rigid 1,3-enchained cyclobutanes in the backbone (Scheme 1B). However, extending [2 + 2] cycloaddition more broadly to other linear α,ω-dienes to incorporate methylene spacers between 1,3-cyclobutane groups in a more controlled manner has been synthetically challenging due to various competing isomerization pathways leading to the loss of terminal alkenes.^32,33^ Cross-cycloaddition of butadiene and α,ω-dienes was explored as a strategy to avoid such deleterious isomerization and access more controlled methylene-modified poly­(oligocyclobutane) structures.

Here, we describe the co-oligomerization of butadiene with α,ω-dienes as comonomers to insert methylene units into the resulting DVOCB oligomers to disrupt crystallinity (Scheme 1C). Both choices of α,ω-diene and comonomer ratio were used to control the methylene-to-cyclobutane ratio and distribution, enabling targeted design of a wide range of methylene-modified poly­(oligocyclobutane)­s that feature improved toughness and ductility and enable processing at more accessible temperatures. In addition, the effects of methylene modification on the rotator phase were elucidated through a combined computational and experimental approach. Finally, the interplay among these changes in crystallinity, rotator phase transitions, and mechanical properties exhibited by this series of modified polymers was examined.

## Results and Discussion

### Synthesis and Deoligomerization of Cross-[2 + 2] Oligocyclobutanes

To incorporate methylene spacers between cyclobutane units along the backbone of cyclobutane-containing oligomers and polymers, the iron-catalyzed cross-[2 + 2] cycloaddition of butadiene and α,ω-dienes was explored. To provide a range of methylene lengths, 1,4-pentadiene (PD), 1,5-hexadiene (HD), and 1,7-octadiene (OD) were chosen as representative α,ω-dienes. However, the pyridine­(diimine) (PDI) iron dinitrogen complexes [(^Me^RPDI)­Fe­(N_2_)]_2_(μ-N_2_) (^Me^RPDI = 2,6-(2,6-Me_2_-C_6_H_3_–N = CR)_2_C_5_H_3_N, R = Me, Et, ^i^Pr) that catalyze the [2 + 2] cycloaddition-oligomerization of butadiene to form DVOCB^11,12^ are also known to promote rapid intramolecular (cyclo)­isomerization of PD to piperylene,^34^ HD to methylenecyclopentane,^32^ and OD to 1-methyl-2-methylenecyclohexane or trans-[4.2.0]-bicyclooctane.^33^ It was hypothesized that using an excess of butadiene relative to the α,ω-diene comonomer would avoid these unwanted (cyclo)­isomerization reactions due to the strong coordination affinity of butadiene to the reduced iron center.^35^ The intramolecular isomerization of α,ω-dienes likely arises from competing cyclometalation of the aryl substituents to form iron hydrides. However, in the presence of a sufficient excess of butadiene, the formation of (PDI)­Fe­(η^2^, η^2^-C_4_H_6_), which can only engage in productive [2 + 2] cycloaddition, was expected to outcompete this process.

To probe the feasibility of cross-[2 + 2] cycloaddition with butadiene and α,ω-dienes, the cycloaddition of butadiene with PD was evaluated with BD:PD ratios of 2:1, 3:1, 4:1, 6:1, and 10:1 and with ((^Me^EtPDI)­FeN_2_)_2_(μ-N_2_) as the iron precatalyst (Scheme 2A).^12^ This specific iron complex was chosen due to a combination of factors including its relative ease of synthesis, purification, and storage stability. Butadiene was first added to the precatalyst to generate (^Me^EtPDI)­Fe­(η^4^-C_4_H_6_) in situ followed by the introduction of PD. After 24–48 h at 50 °C, 100% conversion of monomers was confirmed by ^1^H NMR spectroscopy, and the resulting oligomers were isolated in quantitative yield with morphologies ranging from colorless viscous oil (2:1, 3:1) to colorless semisolid residue (4:1 and 6:1) to off-white semicrystalline powder (10:1). The oligomers synthesized with a BD:PD ratio of *n*:1 will be referred to by their dominant oligomer composition and denoted as B*n*P1 (B2P1, B4P1, B6P1, and B10P1). In most cases, the dominant oligomer composition is equivalent to the starting ratio of comonomers, with one exception (*vide infra*). The successful formation of cross-[2 + 2] oligomers was confirmed by 1D and 2D NMR spectroscopy. This analysis established both PD incorporation at the ends of oligomer chains, producing allyl end groups, as well as PD incorporation within the chains, resulting in methylene units flanked by cyclobutyl groups. It should be noted that the cyclobutyl nomenclature used here is synonymous with cyclobutanedienyl. The average *M*_n_ of oligomers and the ratio of vinyl to allyl chain ends were determined by relative ^1^H NMR integration and correlated with increasing butadiene content (Table 1). It was observed that increasing the fraction of butadiene to α,ω-diene comonomer (increasing *n*:1) led to materials with increased molecular weight and crystallinity. Notably, propenyl chain ends (2%) that arise from [2 + 2] cycloaddition with piperylene were observed in the NMR spectrum of B2P1 (Figure S1–S2), indicating that minor amounts of isomerization occurred under moderate excesses of butadiene. The lack of propenyl chain ends in the other oligomers indicated that isomerization was eliminated with larger excesses of butadiene.

**Scheme 2:**
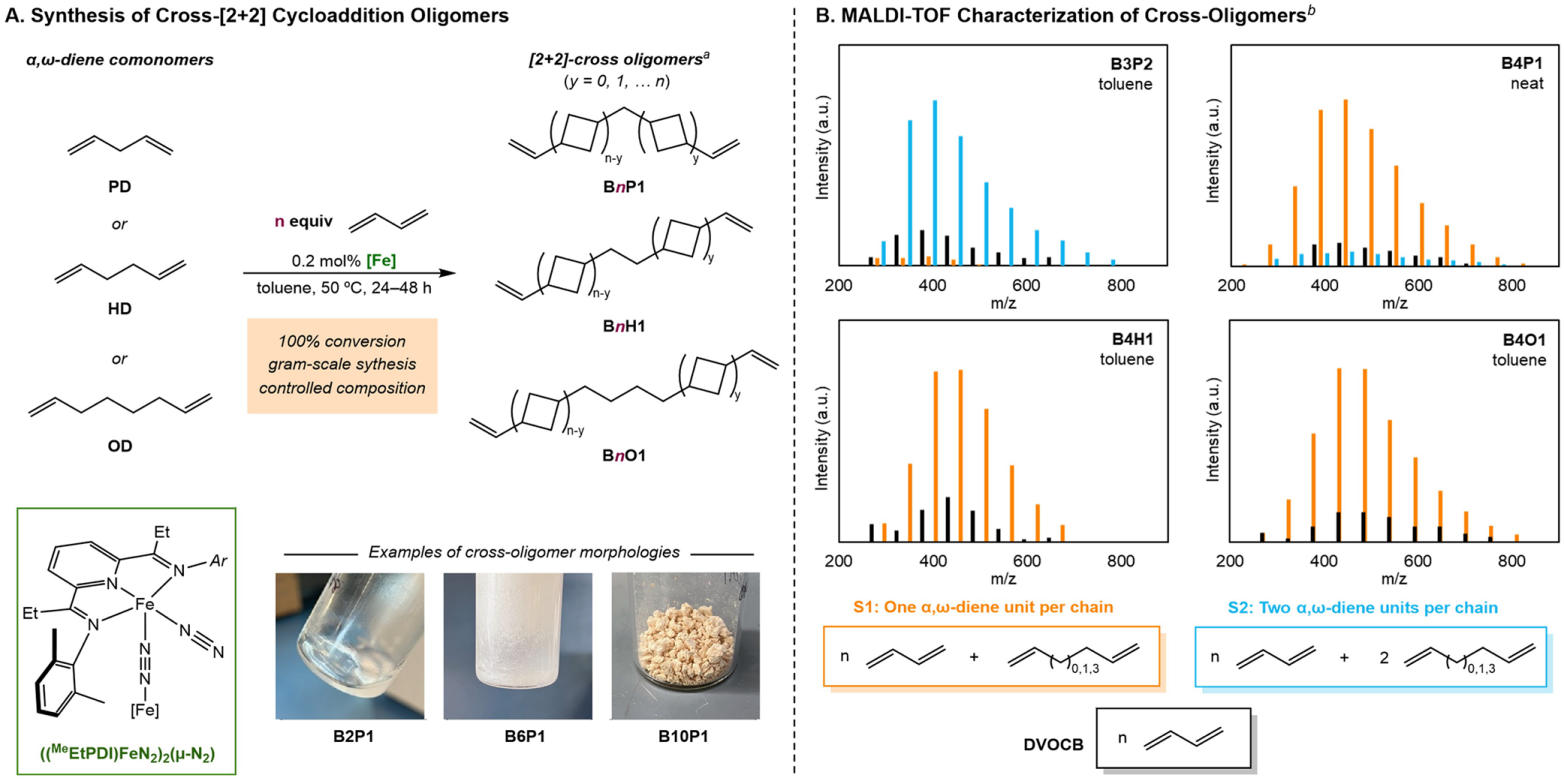
Synthesis of Cross-[2 + 2] Cycloaddition Oligomers

a Approximate representations based on MALDI data.
b 2,5-dihydrobenzoic acid was chosen as the matrix, and silver nitrate as cationizing agent where the Ag+ isotopic pattern for each peak was omitted for clarity.

**Table 1:** Summary of Molecular Characterization Data for Cross-Oligomers

Oligomer	Vinyl:Alkenyl^a^	*M*_n_(g/mol)^b^	S1:S2:DVOCB^c^	Đ^c^
B2P1	76:22 (2% isom)	284	85:9:6	1.02
B3P1	76:24	260	^d^	^d^
B3P2	80:20	299	4:80:16	1.05
B4P1	80:20	317	85:8:7	1.09
B6P1	88:12	376	73:7:20	1.04
B10P1	94:6	587	^e^	^e^
B4H1	85:14	326	84:16	1.04
B6H1	89:11	380	88:12	1.04
B4O1	83:17	245	82:18	1.07
B6O1	86:14	408	77:23	1.09

a Alkenyl = allyl, butenyl, or hexenyl.

b Determined by total proton integration relative to end groups by ^1^H NMR spectroscopy assuming an average oligomer composition of *n* equiv butadiene and 1 equiv *α,ω*-diene.

c Determined by MALDI-TOF MS and reported as averages over multiple spots (>3) and scans (>3 per spot).

d Not quantified by MALDI-TOF MS.

e Not soluble in conventional solvents at room temperature precluding analysis by MALDI-TOF MS.

With the development of a successful method for the cross-[2 + 2] oligomerization of BD and PD, this approach was extended to HD and OD comonomers. At low excesses of butadiene to PD (2:1), PD isomerization was observed, while at high (10:1) ratios, the resulting oligomer was insoluble in organic solvents at ambient temperature. Therefore, BD:α,ω-diene ratios of 4:1 and 6:1 were used for the synthesis of oligomers with HD or OD comonomers to afford B*n*H1 (B4H1, B6H1) and B*n*O1 (B4O1, B6O1), respectively. All oligomers were isolated as colorless semisolid materials. Characterization by NMR spectroscopy (Figures S1–S10) confirmed the incorporation of α,ω-diene units both along the main chain as well as at chain ends, consistent with observed structures in B*n*P1.

Matrix-assisted laser desorption ionization-time-of-flight (MALDI-TOF) mass spectrometry was used to analyze the composition and distribution of cross-[2 + 2] cycloaddition oligomers. MALDI-TOF analysis of B*n*P1 oligomers revealed that three distinct populations of oligomers were possible: cross-oligomers with incorporation of one α,ω-diene (S1), cross-oligomers with two α,ω-dienes (S2), and oligomers with no α,ω-dienes (DVOCB) (Scheme 2B). Due to the potential differences in ionization efficiency of different oligomer populations and chain lengths, it is important to note that the MALDI-TOF results are not quantitative but provide a useful qualitative understanding of the composition of cross-oligomers. In the cases of B*n*H1 and B*n*O1 oligomers, only S1 and DVOCB were observed. While DVOCB was present in all isolated cross-oligomers, it comprised a minor fraction of the product composition, ranging from 12% to 23%. The dominant series of oligomers was S1 for all cross-oligomers synthesized, with the exception of B4P1 for which the dominant oligomer series depended on the specific reaction conditions (S2 when synthesized in toluene and S1 when synthesized neat). Analysis of the S2 product of B4P1 indicates a true composition of B3P2, as labeled in Scheme 2B. Coupled with the estimated *M*_n_ by ^1^H NMR spectroscopy, the average oligomer chain for a given cross-oligomer distribution corresponded to the starting monomer composition; specifically, a starting butadiene:α,ω-diene ratio of *n*:1 produces cross-oligomers with an average composition of *n* units of BD and 1 unit of α,ω-diene. This indicated that oligomer growth is primarily driven by the addition of butadiene to growing oligomer chains in a chain-growth fashion, until all butadiene is consumed (Scheme S2). However, the ability of PD to be incorporated twice within a chain to give S2-type oligomers, which is not observed with HD or OD, may indicate the possibility of alkene–alkene [2 + 2] cycloaddition under specific conditions. As the S2 structure for B4P1 effectively incorporates 3 units of butadiene and 2 units of pentadiene, the sample with a dominant S2 composition is also denoted B3P2. The lower incorporation of butadiene into the B3P2 structures likely resulted in the production of additional DVOCB that consumes the excess butadiene. The synthesis and characterization data for cross-[2 + 2] oligomers are summarized in Table 1.

While DVOCB is composed of 1,3-enchainment of cyclobutanes, the regiochemistry of these cross-oligomers may be different. Iron-catalyzed alkene–alkene cycloaddition is known to yield 1,2-substituted cyclobutanes, while alkene-diene and diene–diene cycloaddition afford 1,3-substituted cyclobutanes (where dienes refer to conjugated dienes, and nonconjugated dienes behave similar to terminal alkenes).^11,36^ Therefore, chain growth by the addition of BD is expected to result in 1,3-enchainment, whereas cycloaddition between two α,ω-dienes, between two oligomer chain ends, or between an α,ω-diene and an oligomer chain end would result in 1,2-enchained cyclobutanes. However, the [2 + 2] cycloaddition between oligomer chains was not observed, as [2 + 2] cycloaddition involving sterically demanding alkenes is unfavorable (see additional discussion in Section S2). For B*n*H1 and B*n*O1 cross-oligomers that are composed of an average of one α,ω-diene and *n* butadiene units, the primary regiochemistry is therefore expected to be 1,3-enchainment of cyclobutanes. In the case of B*n*P1 oligomers, which contain either one or two PD units per oligomer chain, a mixture of 1,3-enchainment and 1,2-enchainment is expected. However, due to the complexity of the NMR spectra arising from the multiple possible series and isomers of oligomers, 1,3- and 1,2-substitution of cyclobutanes cannot be definitively differentiated.

To probe the feasibility of chemical recycling for the cross-oligomers, the oligomer B4P1 was synthesized neat (predominantly 1 PD unit per chain) and subjected to retro-[2 + 2] cycloaddition. Using an established liquid nitrogen trap strategy,^12^ B4P1 was treated with 2.5 mol % ((^Me^EtPDI)­FeN_2_)_2_(μ-N_2_)) in mesitylene at 90 °C for 36 h. The resulting volatiles were collected and quantified by ^13^C NMR spectroscopy relative to an internal standard, indicating 57 wt % yield of butadiene, 5 wt % yield of pentadiene, and 18 wt % yield of vinylallylcyclobutane (Scheme 3). Together, the volatiles constitute 80 wt % conversion of the starting oligomer and demonstrate that, like DVOCB, cross-oligomers are amenable to depolymerization by retro-[2 + 2] cycloaddition.

**Scheme 3:**
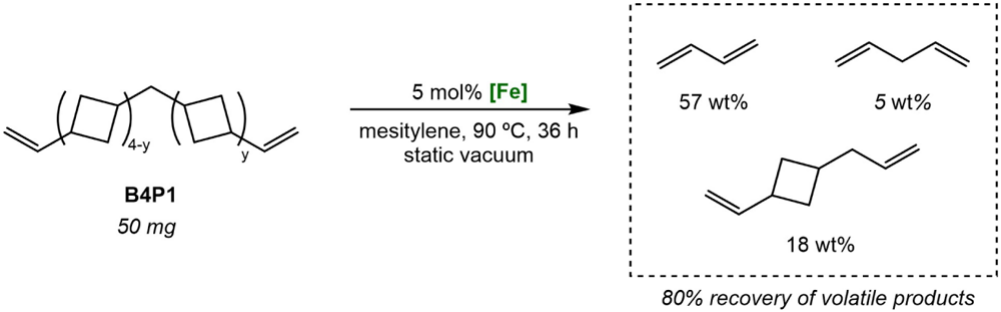
Chemical Recycling of B4P1 by Retro-[2 + 2] Cycloaddition

### Synthesis of Cross-[2 + 2] ADMET Polymers

With a series of modified cyclobutyl-enchained oligomers bearing methylene spacers in hand, chain-extended polyolefins were generated through ADMET polymerization, analogous to the chain extension of DVOCB to synthesize pDVOCB.^12^ Polymerizations were carried out on 2.0 g of oligomer using the same high-temperature metathesis catalyst M1002 and high-temperature solution ADMET conditions previously used for pDVOCB (Scheme 4A).^12^ ADMET polymerization of the cross-[2 + 2] oligomers B*n*P1, B*n*H1, and B*n*O1 resulted in polymers denoted as pB*n*P1, pB*n*H1, and pB*n*O1, respectively. Collectively, this series of polymers will be termed pB*n*X1 (X = P, H, or O). These polymers were obtained in good (80–97%) yields as white or off-white solids with morphologies ranging from semicrystalline powders to rubbery flakes.

**Scheme 4:**
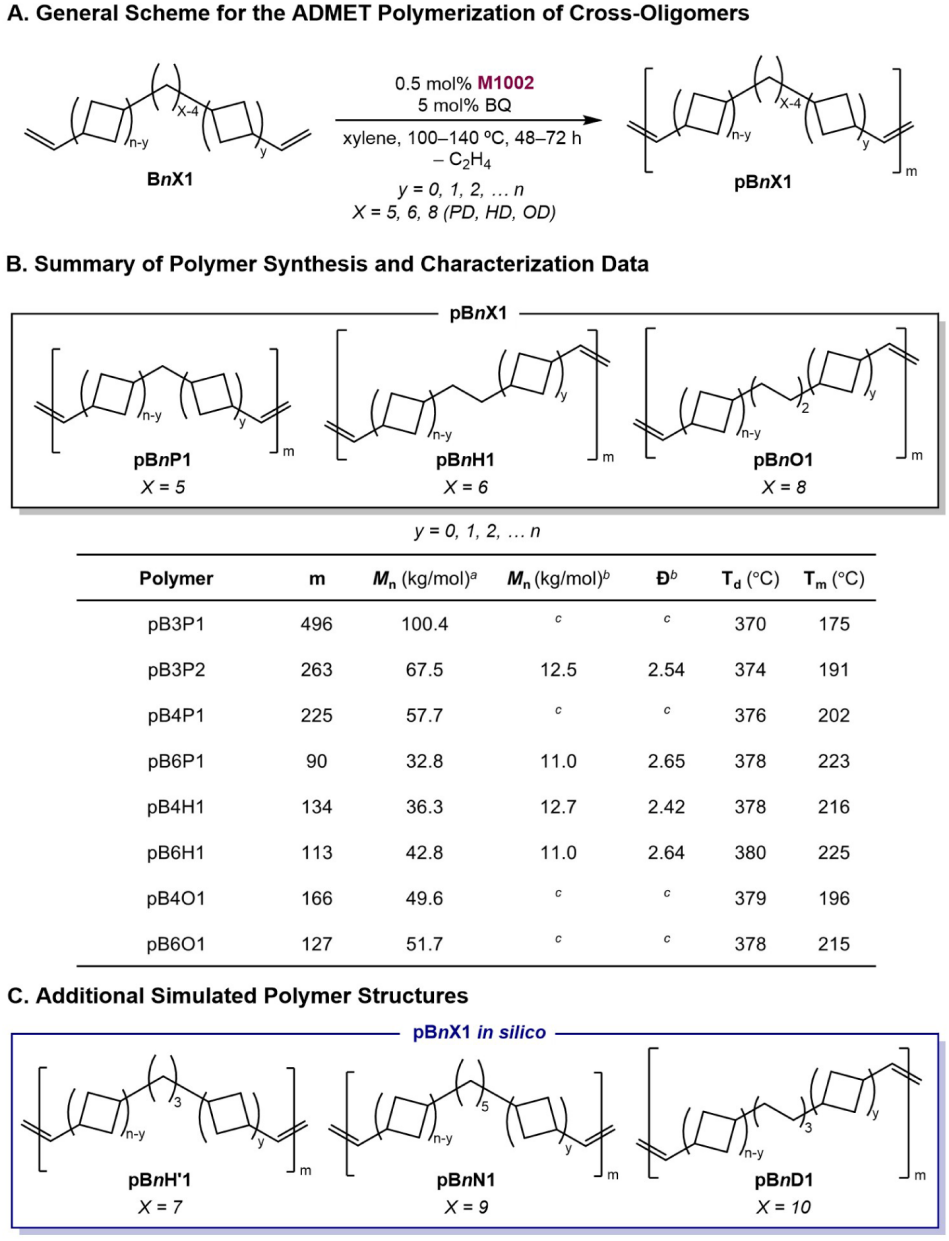
ADMET Polymerization and Characterization of Methylene-Modified Polycyclobutanes

a Determined by high-temperature ^1^H NMR spectroscopy in TCE-*d*_2_ at 140 °C.
b Determined by HT-GPC in TCB at 145 °C relative to polyethylene.
c HT-GPC not available.

Consistent with commodity polyolefins and pDVOCB, pB*n*X1 products were insoluble in common organic solvents at ambient or moderate temperatures. *M*_n_ was determined by end-group analysis with high-temperature ^1^H NMR spectroscopy in 1,1,2,2-tetrachloroethane-*d*_2_ (TCE-*d*_2_) at 140 °C that was able to distinguish the internal alkene protons from the terminal alkene methylene protons. High-temperature ^13^C NMR spectroscopy confirmed the presence of internal double bonds arising both from metathesis between vinyl end groups as well as metathesis between vinyl and allyl end groups (polymer ^1^H and ^13^C NMR shown in Figures S19–S30). Weight-average molecular weights (*M*_w_) and dispersities (Đ) were determined relative to polyethylene by high-temperature GPC (HT-GPC) in 1,2,4-trichlorobenzene (TCB) at 145 °C. As anticipated, the values of *M*_n_ from end-group analysis by ^1^H NMR spectroscopy were consistently higher than the values of *M*_n_ from HT-GPC, which are attributed to the difference in hydrodynamic volume between ADMET polymers and the linear polyethylene used for calibration. While the formation of cyclic products is also possible and would lead to higher values of *M*_n_ by end-group analysis, the rigid enchained cyclobutane sequences make significant cyclic formation unlikely but nevertheless is a consideration. Notably, the polymers formed from cross-oligomers exhibited noticeably improved solubility in the polymerization reaction compared to polymers formed from DVOCB, likely contributing to the higher polymer molecular weights obtained for pB*n*X1 polymers compared to pDVOCB. Synthesis and molecular and thermal characterization details are summarized in Scheme 4B. In addition to the pB*n*P1, pB*n*H1, and pB*n*O1 (synthesized using α,ω-dienes containing X = 5, 6, and 8 carbons, respectively), three additional pB*n*X1 structures were generated *in silico* for computational studies (X = 7, 9, and 10, Scheme 4C). The full series of six pB4X1 polymers (X = 5 – 10) is examined in simulations detailed in a later section.

### Impact of Methylene Modification on Melting Temperature

All copolymer samples exhibited moderately suppressed melting temperatures compared to standard pDVOCB, with copolymer *T*_m_ ∼ 200 – 220 °C (compared to pDVOCB with *T*_m_ ∼ 260 °C) as shown in Figure 1. While the methylene spacers were expected to disrupt crystallization imparted by the cyclobutyl sequences, the increased backbone flexibility may accommodate crystallization. These competing effects may be responsible for the retention of high *T*_m_ and large melting enthalpies (Figure S40 and Table S3). Similar effects were previously observed in backbone-modified pDVOCB, where hydrogenation increases *T*_m_ due to increased chain flexibility, while isomerization decreased *T*_m_ due to crystal defects induced by increased chain irregularity.^37^ For each family of pB*n*X1 polymers, the polymer *T*_m_ increases with increasing butadiene content (i.e., increasing *n*), which also corresponds to the number of cyclobutane units in an average oligomer structure. This trend is consistent, albeit with slightly depressed *T*_m_ values, with previous work on DVOCB­(*n*) and pDVOCB­(*n, m*) where thermal transition temperatures increase with cyclobutyl sequence length. Additionally, this behavior is consistent with the trends in the pB*n*X1 crystal thickness as measured in small-angle X-ray scattering (SAXS) (Figure S49). Polymers with increased cyclobutyl sequence length (e.g., pB4P1 compared to pB3P1) generally exhibit an increased long period, which represents the combined thickness of the crystal and amorphous layers. Because the relative degree of crystallinity increases with the number of enchained cyclobutanes in the oligomeric repeat unit (see melting enthalpy comparisons in Table S3), this increase in the long period must also correspond to an increase in crystal thickness and supports the increase in *T*_m_.

**Figure 1:**
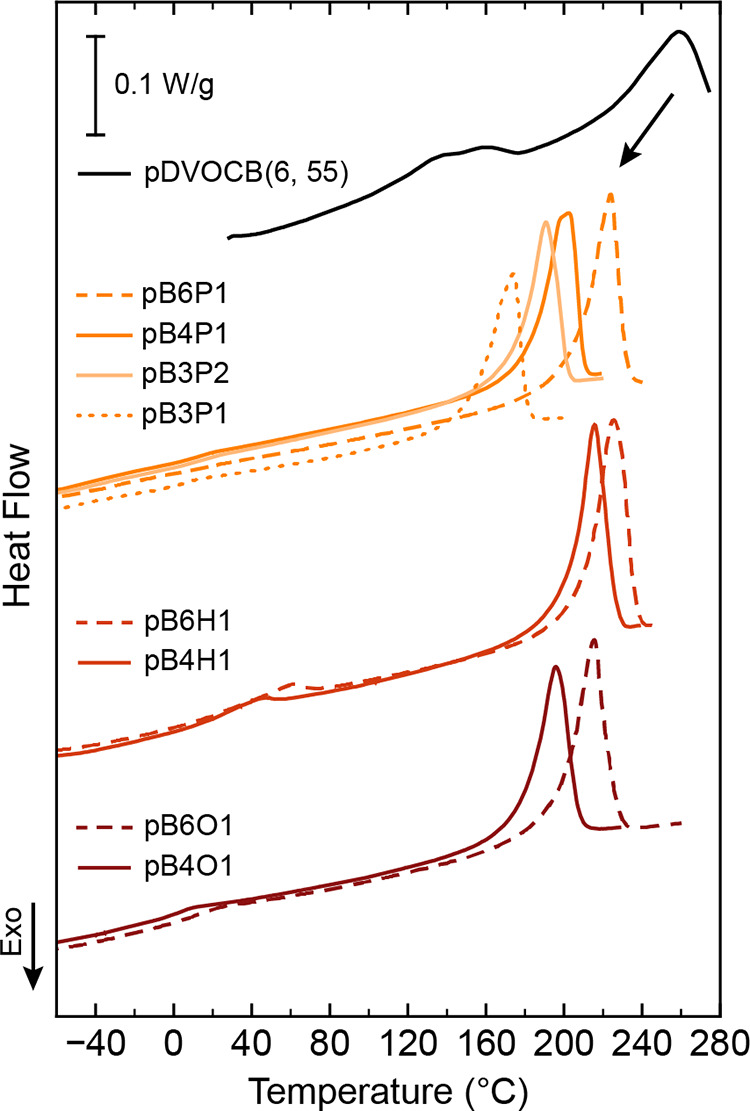
DSC measurements for pB*n*X1 polymers, including several BD:PD polymers (orange), BD:HD polymers (scarlet), and BD:OD polymers (maroon), compared to a representative pDVOCB­(6, 55) sample (black). All data shown is taken from the second heating cycle at a rate of 5 °C/min. Different families of polymers are vertically shifted for clarity. For all pB*n*X1 families, 4:1 (solid) and 6:1 (dashed) samples are shown. For the BD:PD polymers, additional 3:1 (pB3P1, dotted line) and 3:2 (pB3P2, light orange) samples are included.

Interestingly, the *T*_m_ across all pB*n*X1 polymers does not decrease monotonically with increasing length of the α,ω-diene comonomer. For a given *n*:1 ratio, hexadiene-based copolymers consistently show higher melting temperatures than pentadiene or octadiene copolymer samples (for *T*_m_ peak values, see Scheme 4). This nonlinear melting behavior with methylene spacer length is likely due to odd–even entropic effects related to molecular conformation; such effects are common in polymers composed of alternating stiff and flexible segments.^38^

In addition to suppressing the melting temperature, methylene modification alters *T*_g_ and rotator phase behavior compared to pDVOCB. Standard or backbone-modified pDVOCB experiences two rotator transitions between *T*_g_ and the melting point, corresponding to two endothermic peaks in calorimetry.^37^ In all pB*n*X1 polymers, only a single weak transition was observed near 0–40 °C (∼180 °C lower than *T*_m_). This transition occurs at a temperature range that may be appropriate for either the *T*_g_ or the crystal-to-rotator phase transition (T_r_) based on previous studies of cyclobutane-containing polymers.^12,37^ While the glass transition was observed in pDVOCB between 60 – 80 °C, the increased chain mobility imparted by the methylene segments would result in a lower *T*_g_. We expect both *T*_g_ and T_r_ to have significant impacts on pB*n*X1 mechanical behavior.

### Mechanical Testing of Methylene-Modified Copolymers

With an established series of methylene-modified polymers, the corresponding changes in mechanical behavior were examined. Previous studies demonstrated that although pDVOCB­(*n, m*) possesses a high modulus comparable to commercial polyolefins, its properties and applications are severely limited by extreme brittle behavior with failure at strains as low as 10%.^12^ This limitation is largely due to the high crystallinity as well as the consequently low molecular weights that can be achieved through standard pDVOCB synthesis.

Tensile testing of pB4X1 polymers established significantly enhanced ductility compared to standard pDVOCB, with strain at break values reaching over 400% engineering strain (Figure 2). Multiple factors may contribute to these dramatic changes in mechanical properties. Compared to pDVOCB­(*n, m*), pB*n*X1 polymers can access significantly higher molecular weights due to their improved solubility during reaction (*M*_n_ range ∼36–100k compared to ∼ 15–20k in pDVOCB).^12,37^ This increased molecular weight is expected to allow significant tie-chain formation and enable greater distribution of stress and higher ductility.^39,40^ This molecular weight dependence of ductility is also seen in pDVOCB, allowing strain to increase from ∼6% to ∼42% strain at break between pDVOCB­(5, 40) (*M*_n_ ∼ 12 kg/mol) and pDVOCB­(5, 67) (*M*_n_ ∼ 20 kg/mol).^12^ As noted in Figure 2, the pB*n*X1 polymers that consistently reach strains ∼400% are the samples with higher molecular weights (58 kg/mol for pB4P1 and 100 kg/mol for pB3P1). These samples experience stable neck propagation that extends through the entire sample length before failure. In comparison, the pB4H1 and pB4O1 polymers (*M*_n_ ∼ 36 and 50 kg/mol, respectively) consistently fail at relatively lower strains from 100% to 250% and experience only partial neck propagation (Figure S58–S59). The molecular weights of these polymers may be too low to enable stable neck propagation, leading to premature failure at incomplete necking. Unstable neck propagation is one indication that the molecular weights are in the transition region between brittle and ductile behavior.^39,41^ While pDVOCB is in the brittle regime, and pB4P1 and pB3P1 appear to be in the ductile regime, it is possible that the intermediate strain values of pB4H1 and pB4O1 indicate that they are in the transition regime. If higher molecular weights were synthesized, it is likely that these samples may also reach strains much higher than those currently observed.

**Figure 2:**
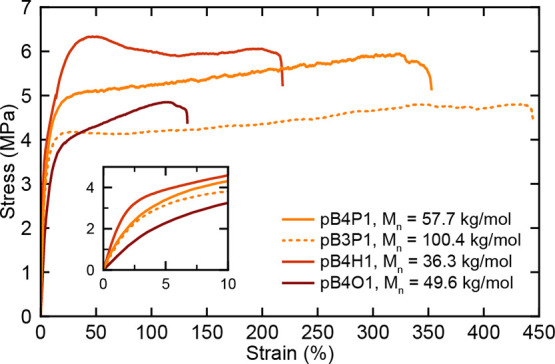
Tensile testing results for selected polymer samples, including the pB4X1 series (solid lines) and pB3P1 sample (dashed line). Testing was performed at a rate of 3% engineering strain per minute. The inset shows a magnified view of the 0 – 10% strain region.

In addition to the higher molecular weight, pB*n*X1 polymers also likely exhibit lower *T*_g_, lower T_r_, and lower crystallinity, which will further alter mechanical behavior and ductility. Ultimately, the increased molecular weight likely has the most significant effect toward increased ductility, but the lower crystallinity and *T*_g_ help to explain the lower yield stress and tensile strength compared to pDVOCB. The effects of crystal-to-rotator transitions on mechanical behavior are not clearly understood, especially considering the rarity of rotator phase behavior in polymers. Studies of the rotator phase transition in hydrogenated polynorbornene (hPN) found that polymer yield stress dropped sharply after passing through the crystal-to-rotator transition (denoted *T*_cc_ in that work), but the Young’s modulus was not significantly affected by the transition.^42^ However, previous studies of rotator transitions in pDVOCB have noted a relaxation and decrease in modulus corresponding to the rotator transition.^12,37^ As the yield stress of all pB*n*X1 polymers is significantly lower than that of pDVOCB samples, it is important to probe the contributions of the rotator phase behavior to the room-temperature mechanical behavior.

While ductility is significantly enhanced, the Young’s modulus of all pB*n*X1 polymers is significantly reduced compared to pDVOCB. This lower modulus is primarily due to a suppression in *T*_g_ below room temperature, resulting from the increased backbone flexibility from the methylene units. Furthermore, while all pB*n*X1 polymers were tested at the same strain rate as pDVOCB (3% engineering strain per minute), the lower *T*_g_ of pB*n*X1 may have allowed more stress relaxation during tensile testing, leading to the observed reduced stress values. Tensile testing of pB4P1 at higher strain rates (10%/min and 100%/min) confirms that the stress values increase by over 20% and strain at failure reaches over 450%, as demonstrated in Figure S55. Notably, the higher strain rates tested were all sufficient to allow the stable neck propagation needed for the high observed ductility. Additionally, pB*n*X1 polymers may possess a slightly lower crystallinity than pDVOCB based on melting enthalpy comparisons (Figure S40). Within the pB4X1 series, pB4H1 exhibits the highest Young’s modulus and tensile strength. Furthermore, the T_r_ for pB4H1 (∼40 °C) is higher than that for the remaining polymers; pB4P1 and pB4O1 exhibit rotator transition temperatures near and below room temperature, respectively, indicating that both polymers may be in a rotator phase at ambient conditions. It is expected that pB*n*X1 polymers would soften after passing through T_r_, which may partially explain why pB4P1 and pB4O1 are softer than pB4H1. Additionally, the yield stress of pB4H1 is higher, which may relate to differences in crystallinity or the differences in the T_r_ transition between pB4H1, pB4P1, and pB4O1 based on the relationship between T_r_ and polymer yield stress observed in literature.^42^

Ultimately, the toughness of pB*n*X1 polymers exceeds the toughness of pDVOCB polymers despite the lower modulus values; pB3P1 has an average toughness of 18.1 MJ/m^3^ compared to ∼1.2 MJ/m^3^ for pDVOCB­(5,40) (Table S4).^12^ This represents over an order of magnitude improvement in toughness and indicates that pB*n*X1 polymers could be tuned for applications requiring high toughness and ductility.

### Effects of Crystalline Phases on Mechanical Behavior of Copolymers

Due to the many factors affecting the mechanical behavior of pB*n*X1 polymers, it is difficult to deconvolute the effects of the changing T_r_, crystallinity, *T*_g_ and increased molecular weight. The increased understanding of how methylene modification alters the rotator and crystalline phases in cyclobutane polymers motivates further investigation of the mechanisms of tensile deformation occurring in these soft crystalline polymers. Overall, the high ductility of pB*n*X1 polymers is achieved through the necking and neck propagation that occur during deformation (Figure 3B). This necking behavior is also observed in many ductile commercial polymers, such as high-density polyethylene, and is desirable in many applications requiring high deformation before failure.

**Figure 3:**
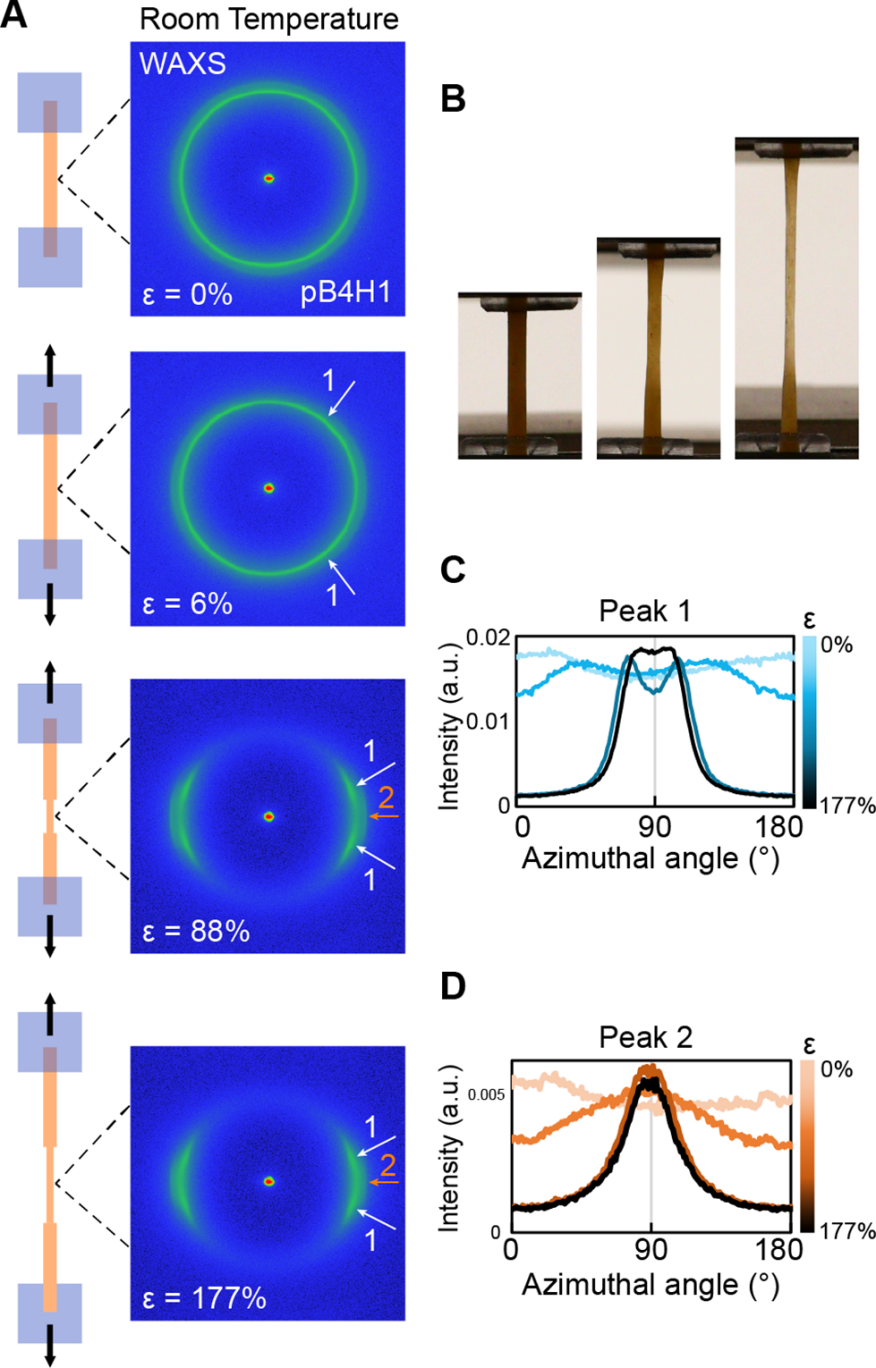
Examination of deformation in pB4X1 polymers. (A) In situ WAXS measurements taken during tensile elongation of the pB4H1 sample, next to schematics depicting necking and neck propagation. The draw direction is vertical. (B) Photos of pB4P1 sample taken during tensile testing showing necking behavior. (C) Azimuthal orientation of the lower q peak (peak 1) with increasing strain. (D) Azimuthal orientation of the higher q peak (peak 2).

To gain more insight into the deformation mechanisms of pB*n*X1 polymers, the changes in crystallinity during tensile deformation were examined by in situ X-ray scattering. Wide-angle X-ray scattering (WAXS) measurements of pB4H1 indicate the development of a four-spot pattern that reorients with increasing strain, indicated by the white arrows in Figure 3A. These changes in the orientation of pB4H1 crystalline peaks are consistent with known deformation mechanisms in polyethylene and similar semicrystalline polymers. When experiencing tensile strain, the preferred slip direction is at an angle offset relative to the chain axis. With increasing strain, the crystals rotate in opposite directions to orient with the preferred slip direction. Eventually, the crystal lamellae fragment and reorient in the strain direction as fibrils.^43,44^ Here, the peak designated as “Peak 1” in Figure 3 is analogous to the 110 peak in polyethylene, which shows a four-spot pattern that reorients closer to the equator with increasing strain. Similarly, the 200 peak in polyethylene is analogous to “Peak 2” in pB4H1, which appears at slightly higher q values and shows an increasing orientation at the equator with increasing strain.

At the room temperature conditions for the tensile testing previously shown in Figure 2, pB*n*X1 samples are expected to be near the crystal-to-rotator transition temperature. The samples may soften after passing through the rotator transition, so the location of the T_r_ relative to room temperature may lead to significant changes in the mechanical properties. In particular, a higher T_r_ (∼40 °C) may be responsible for the higher tensile strength and modulus exhibited in pB4H1 compared with pB4P1 or pB4O1 samples. This difference motivated further investigation into how the rotator transition may be affecting the modes of polymer deformation, as the rotationally disordered structure may facilitate fine or coarse slip mechanisms. In situ X-ray scattering during tensile deformation was performed on a pB4H1 sample held at 60 °C (∼T_r_ + 20 °C) to examine the effects of the rotator phase. While the initial WAXS results recorded at 0% strain confirmed that pB4H1 was in the hexagonal rotator phase, the WAXS pattern during increasing strain indicated a transformation back to the crystalline phase (Figure S51). The reappearance of the secondary crystalline peak perpendicular to the strain direction shows the crystallization of the polymer back to the monoclinic structure despite the temperature remaining above T_r_. This strain-induced crystallization out of the rotator phase may be relevant for future processing or patterning and may also be responsible for the high toughness of pB4X1 polymers.^45^

### Examination of Low-Temperature Thermal Transitions in Experiments and Simulations

For the following discussions, focus is placed on the pB4X1 polymer series synthesized from a BD:XD ratio of 4:1, namely pB4P1, pB4H1, and pB4O1 samples. Simulations of these three samples were performed as well as three additional pB4X1 structures that exist only *in silico* (pB4H’1, pB4N1, and pB4D1, Scheme 4C) with the goal of elucidating trends with increasing α,ω-diene length. The full simulated series of pB4X1 from effective α,ω-dienes of lengths X = 5–10 carbons is shown in Scheme S3.

Results from both simulation and experiment suggest that pB*n*X1 polymers undergo a broad glass transition below 0 °C and a subsequent crystal-to-rotator transition near 0–40 °C. While DSC measurements of all synthesized copolymers display a weak feature near 0–40 °C, this feature is difficult to conclusively identify from calorimetry alone.

At first appearance, this DSC feature appears consistent with a glass transition (*T*_g_), with a slight step change appearing for pB4P1 and pB4O1 plus an additional endothermic peak which could correspond to enthalpic relaxation for pB4H1 (Figure 4A). Temperature-modulated DSC (TM-DSC) and dynamic mechanical analysis (DMA) were both conducted to further elucidate the behavior of this transition. TM-DSC suggests that the peak may be a crystal-to-rotator transition (T_r_) as the peak did not separate into a clean step change in the reversing heat flow and an enthalpic peak in the nonreversing heat flow that would be expected due to aging overlaid with a *T*_g_ step change (Figure S44). Instead, the peak remains in the reversing heat flow, consistent with the endothermic features of rotator transitions of pDVOCB. This endotherm therefore appears to be more consistent with T_r_ rather than physical aging.^37^ In addition, DMA temperature sweep measurements further support a T_r_, with an additional broad relaxation corresponding to the *T*_g_ at lower temperatures (−50 to −10 °C). As shown in Figure 4C, all three polymers experience a significant relaxation process indicated by the sharp peak in the tan delta and a corresponding drop in the storage modulus by almost an order of magnitude (Figure S52). However, this must be attributed to the rotator transition and not to the glass transition as polymer chains must be significantly above *T*_g_ to possess the mobility required for rearrangement from the crystal to rotator phase. Instead, the broader transition seen in DMA around −50 to −10 °C is attributed to the glass transition; over this range, all three samples exhibit a decrease in the storage modulus by an order of magnitude (Figure S52). The additional drop in modulus that occurs at the rotator transition near 40 °C is consistent with previous studies of the crystal-to-rotator transition in cyclobutane polymers^12,37^ and is analogous to the softening of polyethylene upon undergoing the crystal relaxation process (commonly known as the polyethylene alpha relaxation in literature).^46,47^ The polyethylene alpha relaxation corresponds to a twist-shift motion within the crystalline domains, which increases mobility at the crystal–amorphous interface and reduces the barrier to deformation, which can be observed as a decrease in modulus.^47,48^ While this process is not a rotator transition, both the polyethylene crystal relaxation and the pB*n*X1 rotator transition correspond to additional relaxation processes within the polymer crystals that lower the barrier to deformation and result in a decrease in the modulus.

**Figure 4:**
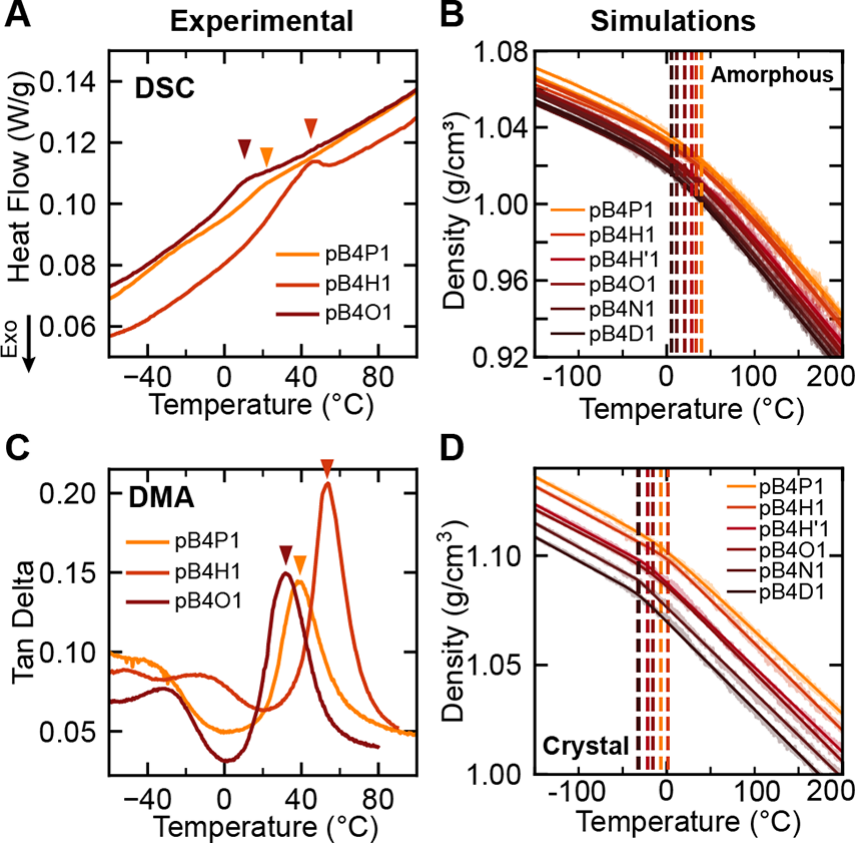
Thermal transitions in pB4X1 polymers. (A) DSC measurements focusing on the −60 to 100 °C window, with features indicated by the arrows. (B) Density simulations on amorphous pB4X1 polymers. The change in slope corresponds to the glass transition *T*_g_. Average *T*_g_ values (dotted lines) show a monotonic trend with diene comonomer length. (C) Tan delta measurements from DMA temperature sweep experiments taken at 1 Hz. (D) Density simulations on ideal crystalline pB4X1 samples, showing a change in slope that corresponds to a T_r_ transition (dotted lines) in the crystalline domains. One ideal crystalline sample is simulated and shown for each pB4X1, showing a nonmonotonic trend in T_r_. Note: the dotted lines for pB4N1 and pB4D1 are almost completely overlapping.

To further investigate these transitions at a molecular level, temperature ramp simulations were performed for pure amorphous and pure crystalline pB4X1 materials, and the density was examined as a function of temperature to probe the thermal transitions present. In amorphous simulations, glass phase transitions are found from slope changes in the density–temperature curve (Figure 4B). The resulting simulated *T*_g_ of pB4X1 amorphous phases shows a monotonic decrease with an increasing diene length. However, this monotonic trend is not consistent with the DSC features observed and supports the TM-DSC and DMA results, suggesting that the crystal-to-rotator transition instead of a *T*_g_ is occurring in this experimental temperature range. Furthermore, these simulated glass transition temperatures are expected to significantly overestimate the experimental *T*_g_ due to effective differences in heating rate compared to experiments.

For crystal material density simulations, slope changes were also observed, indicating a thermal transition, and a series of transition temperatures were extracted (Figure 4D). Because the crystal simulations have no amorphous content, this slope change cannot be a result of the *T*_g_ in the amorphous domains. The increasing rotation of the polymer chain during the simulation suggests that this is a crystal-to-rotator transition, so this extracted transition temperature is referred to as “T_r, density_.” The transition temperature T_r, density_ of pB4X1 polymers shows an odd–even effect: materials with an even number of methylene spacers (pB4H1, pB4O1, pB4D1) show a higher T_r, density_ than materials with an odd number of methylene spacers (pB4P1, pB4H’1, pB4N1), which is consistent with the higher transition temperature of pB4H1 in experiments.

Together, the experimental signals in DMA and DSC and the results of crystal simulations suggest that the pB4X1 materials undergo a rotator phase transition between 0 and 40 °C. Although the glass transition is not directly observable in calorimetry due to the low amorphous fraction and the breadth of the transition, DMA indicates a *T*_g_ between −50 and −10 °C in pB4X1. While amorphous simulations suggest a glass transition at higher temperatures, these simulations overestimate the experimental *T*_g_ due to differences in the temperature ramp rate between experiments and simulations.

Experimental and simulated variable-temperature X-ray scattering confirms the presence of a crystalline-rotator phase transition near 40 °C in pB4H1. As shown in Figure 5, the rotator phase transition is indicated by the change in crystal structure corresponding to the merging of peaks 1 and 2 into a single intermediate peak 3. This transformation was observed in both experimental and simulated X-ray scattering and represents the transformation from a monoclinic crystal structure to a rotationally disordered hexagonal structure. The monoclinic crystal structure is further examined by order parameters in simulations (Supporting Information, Section S9.2). The observed hexagonal phase is akin to the R_II_ rotator phase observed in *n*-alkanes and has been previously observed in DVOCB­(*n*) oligomers and pDVOCB­(*n, m*) polymers.^22,24,37^ The transition temperatures from simulated and experimental X-ray scattering are in agreement, as shown by the plot of the extracted peak positions versus temperature in Figure 5C. Furthermore, the transition temperature from WAXS is in excellent agreement with the temperature of the endothermic feature shown in DSC (44.7 °C from WAXS vs 45 °C from DSC, shown in Figure 7), additionally supporting the idea that the endothermic feature is the result of a crystal-to-rotator transition. The T_r_ of pB4H1 extracted from the simulated X-ray scattering peak transition is different from the T_r, density_ based on density–temperature simulations shown previously. This difference originates from the complex density change during crystal-to-rotator transitions compared to the glass transition, so the common slope-change method used to extract *T*_g_ provides only an estimate of T_r_. To distinguish these two series of T_r_ simulations, the scattering peak transition temperature is referred to as“T_r, peak_” in the following discussion.

**Figure 5:**
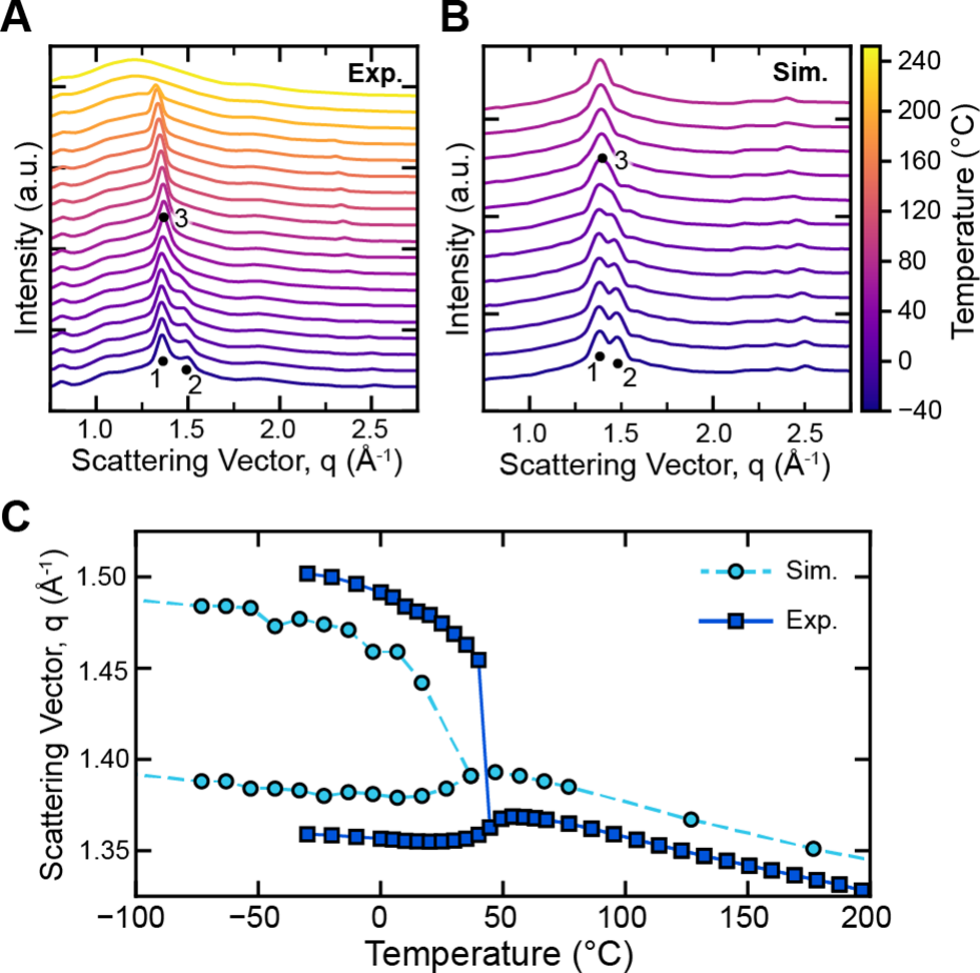
Experimental and simulated X-ray scattering data of the pB4H1 polymer. (A) Experimental variable-temperature WAXS data taken during heating, showing a crystal-to-rotator transition and melting transition. (B) Simulated X-ray scattering data of a pB4H1 polymer crystal, showing a crystal-to-rotator transition. Data is shifted vertically for clarity. Both panels share the temperature scale on the right-hand side. (C) Observation of the crystal-to-rotator transition through experimental and simulated X-ray scattering, indicated by the merging of two scattering peaks into a single peak at T = T_r_. This merging shows the transition into a hexagonal rotationally disordered phase.

Notably, pB4P1 and pB4O1 do not exhibit the X-ray scattering transitions observed in pB4H1; instead of a two-peak to one-peak transition, both samples show only a single crystalline peak throughout the relevant temperature range (Figures S46–S48). This behavior is consistent across experimental and simulated X-ray scattering (Figure S63) and indicates that the samples may undergo a different type of crystal-to-rotator transition than that experienced by pB4H1.

Orientation order parameter analysis in simulation shows that pB4X1 polymers other than that experienced by pB4H1 undergo a transition from a hexagonal crystal to a hexagonal rotator phase (Supporting Information, Section S9.2), resulting in a single scattering peak throughout the T_r_ transition. This also supports the different appearance of the calorimetric feature in pB4P1 and pB4O1 compared to pB4H1, as the endothermic peak is only present when there is a rearrangement of the crystal lattice at the rotator transition. Otherwise, the onset of rotational disorder at the T_r_ presents as a second-order transition in calorimetry, which has previously been observed for rotator transitions in alkanes that are not accompanied by a significant lattice rearrangement.^24^

Molecular details of the crystal-to-rotator transition are revealed by the rotational autocorrelation function (ACF) and mean-squared displacement (MSD) in the simulation. As shown in Figure 6A, crystal pB4H1 shows a rapid decrease in the ACF with heating. Similar behaviors are found in other pB4X1 materials, as reported in Supporting Information, Sections S9.3−S9.4. Through the simulated T_r, peak_ of pB4H1, the effective T_r, peak_ of other pB4X1 materials was extracted by matching the average rotational relaxation times with pB4H1 (Supporting Information, Section S9.3, Figure S68). As shown in Figure 6C, average rotational relaxation times from different materials collapse into the same curve after shifting the temperature by T_r, peak_, which indicates that all pB4X1 materials share very similar rotational relaxation behavior.

**Figure 6:**
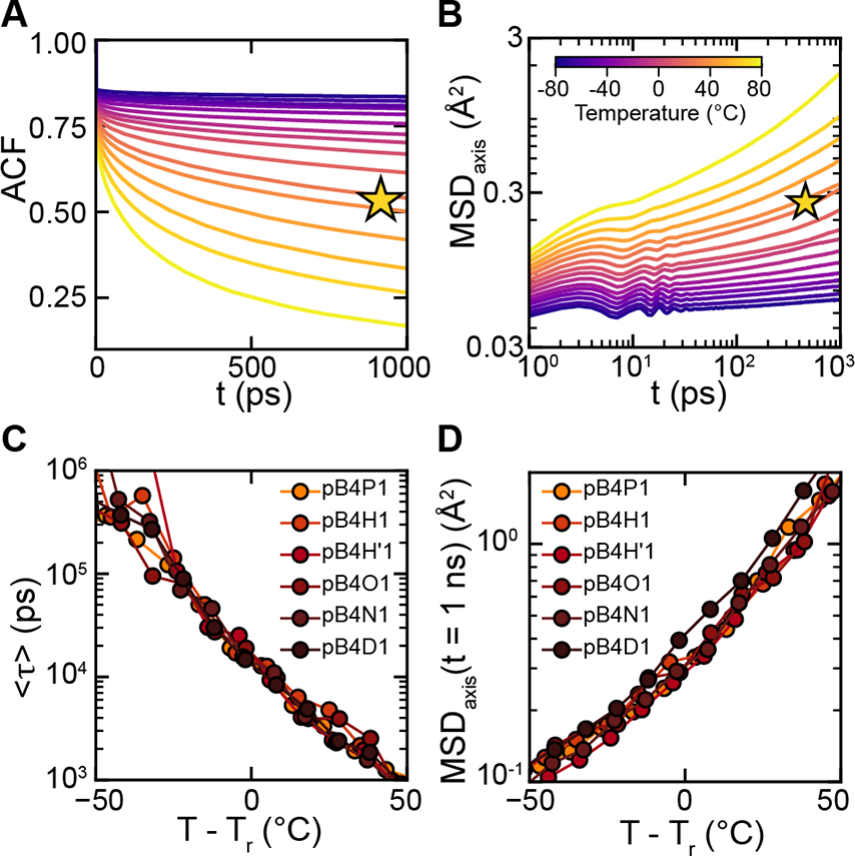
Rotational and translational properties of polymers. (A) The ACF of pB4H1 at different temperatures. (B) The MSD along the axis direction of pB4H1 at different temperatures. (C) The average rotational relaxation time of polymers with respect to T − T_r, peak_. (D) The MSD along the axis at 1 ns for all polymers with respect to T − T_r, peak_. The stars in panels A and B indicate the rotator transition temperatures of pB4H1.

The rotator phase transition observed in pB4X1 is similar to pDVOCB­(4, *m*) simulations (Supporting Information, Section S9.3) and shows a more gradual transition in rotational relaxations, compared to the clearer gap in rotational relaxation times observed in rotator phase transitions in DVOCB and polyethylene-like systems.^22^ This behavior likely arises from the longer polymer backbone, which suppresses the collective chain rotation. Consequently, the rotator phase transition in the polymer is dominated by local monomer rotations rather than whole-chain motion. This shift to local rotations is confirmed by the parallel order parameter simulations, which indicate a higher ordering of local cyclobutane rings compared to the whole chain (Section S9.2). As a result, the distinct discontinuity in the rotational relaxation time, characteristic of collective chain rotation during the transition, disappears in the polymer system. It is also noted that the more gradual rotator phase transition seen here is analogous to the glass transition in the amorphous polymer. At low temperatures, rotational relaxation occurs on long time scales, and the chains’ rotations are largely frozen. At the intermediate temperature range, an increasing fraction of chains starts to rotate frequently, which introduces additional degrees of freedom into the system and changes the slope of the density–temperature curve. At high temperature, the rotational relaxation time is small, as all chains can rotate freely as in the R_II_ rotator phase in *n*-alkane systems.

To examine whether translational motion contributes to the transition, the MSD along the chain axis was also calculated as shown in Figure 6B and D. The translational motion of pB4X1 increases with temperature, but during the rotator phase transition process, the chains remain in the subdiffusion regime (indicated by the small slope in Figure 6B and S69). The MSD shows a similar trend after shifting the temperature by the T_r, peak_, as shown in Figure 6D. Therefore, the translational motion of chains does not play a main role during the rotator phase transition in pB4X1; instead, the rotational relaxation is the primary component of this transition.

The transition temperatures extracted from experimental and computational approaches, including the T_r_, *T*_g_, and *T*_m_ were compared. Transition temperatures from experiments and simulations are plotted in Figure 7.

**Figure 7:**
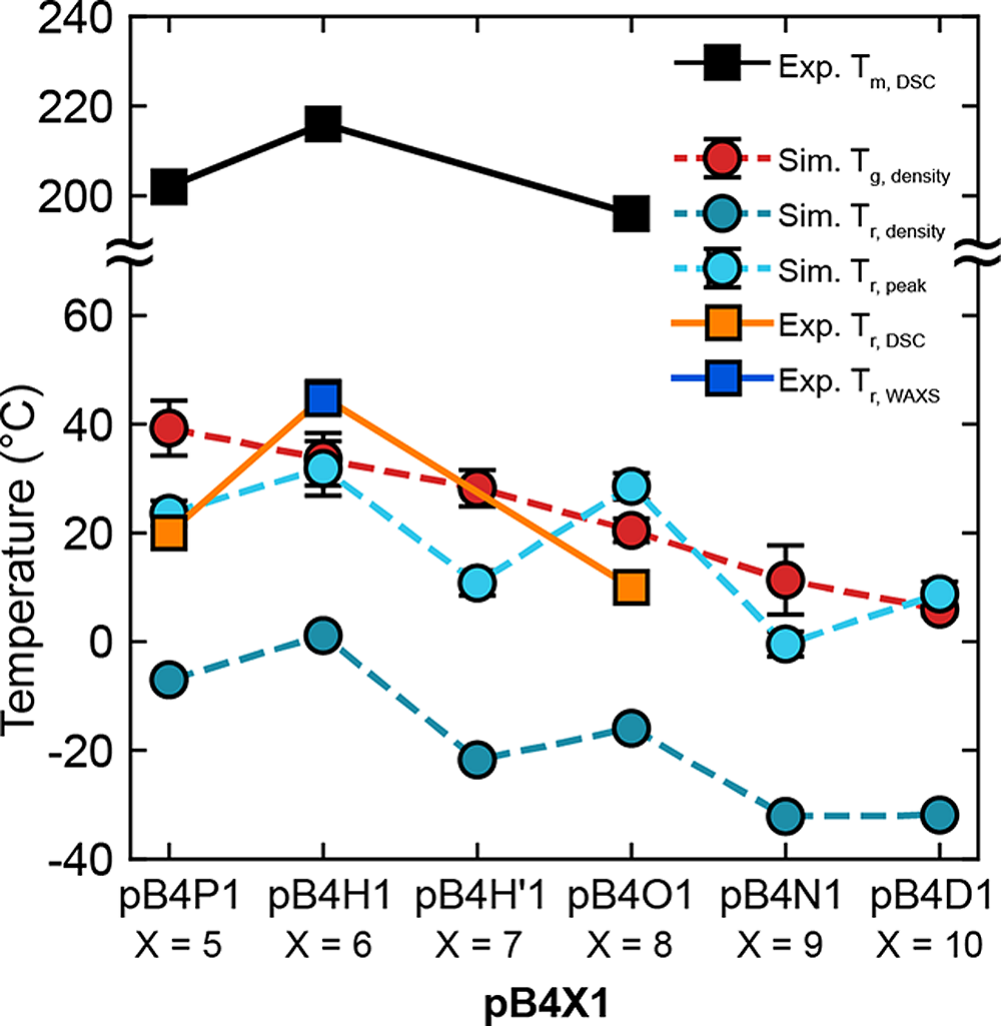
Experimental and simulated thermal transition temperatures for the series of pB4X1 polymers, including the melting temperature (*T*_m_), crystal-to-rotator transition (T_r_), and glass transition (*T*_g_). The experimental T_r_ based on the merging of crystalline peaks observed in WAXS is shown for pB4H1 and agrees well with the T_r_ from DSC (note the overlap of orange and dark blue squares for pB4H1). Simulated *T*_g_ values are extracted from the amorphous polymer density. Simulated T_r_ values are shown from two methods: crystal density and scattering peak, with ACF simulations.

In summary, the results support that pB4X1 polymers experience a rotator phase transition resulting in significant softening between 0 and 40 °C. In simulations, amorphous pB4X1 shows a monotonic decrease of *T*_g_ with increasing length of methylene spacers due to the resulting backbone flexibility, while crystal pB4X1 shows odd–even effects on T_r_ with increasing length of methylene spacer because of the different favored structures and rotational behaviors experienced at low temperatures. While amorphous simulations indicate *T*_g_ values between 15 and 40 °C, these results overestimate the experimental glass transition based on DMA (−50 to −10 °C). This is likely a result of effective differences in the heating rate between experiments and simulations. Furthermore, fully amorphous simulations cannot capture the effects of the crystal–amorphous layer interface, which can significantly influence the *T*_g_ of semicrystalline polymers. When comparing simulation methods of the rotator transition, the T_r, peak_ values (cyan circles in Figure 7) are more tightly connected to polymer chain rotation behavior and more representative of experimental values than T_r, density_ (blue circles in Figure 7). Consequently, the ACF simulations are considered to be a more representative measure of the rotator transition behavior in these systems than crystal density. Overall, the combined effects from the transitions in both the amorphous and crystal phase lead to the higher T_r_ of pB4H1 compared to pB4P1 and pB4O1 observed in the experiments. Notably, the *T*_m_ also shows the same trend as the T_r_ (pB4H1 > pB4P1 > pB4O1), further supporting that the odd–even effects seen in the crystal or rotator phase contribute to both nonmonotonic trends. The higher T_r_ as well as the slightly higher crystallinity observed in pB4H1 are likely both responsible for its increased tensile strength and modulus in comparison to those of pB4P1 and pB4O1 (Figure 2, Tables S4 and S5). The relatively softer pB4P1 and pB4O1 materials both have T_r_ values that are near or below room temperature, meaning that both samples may be in the hexagonal rotator phase rather than a crystalline phase during room temperature tensile testing. Ultimately, these insights into the polymer thermal transitions aid our understanding of room temperature pB4X1 properties and the exploration of potential applications of pB4X1 materials in the future.

While it remains challenging to fully isolate the effects of the crystal-to-rotator phase transition on the mechanical behavior of pB*n*X1 polymers, these findings show that the unique rotator phase behavior of cyclobutane-based polymers offers a rare pathway to tune the mechanical properties and crystallinity. By establishing the relationship between structural modifications and thermal transitions and in turn investigating the interplay between these phase transitions and bulk mechanical properties, this work can guide the future design of highly tunable, recyclable polycycloolefins for a range of applications.

## Conclusions

The iron-catalyzed cross-[2 + 2] cycloaddition of butadiene and a series of α,ω-dienes has enabled the synthesis of a new family of methylene-modified cyclobutane oligomers, where the excess butadiene was used to suppress competing isomerization of the comonomer. The resulting reactant ratio was used to directly tune the oligomer length and content of the methylene spacers. Chain extension of this series of telechelic oligomers by ruthenium-catalyzed ADMET polymerization resulted in a host of chemically recyclable, methylene-modified polycycloolefins. The improved solubility of these materials by introduction of various methylene spacers enabled the synthesis of significantly higher polymer molecular weights, ultimately resulting in notably higher ductility and toughness compared with the highly crystalline, brittle, and relatively low molecular weight pDVOCB polymers. A combined experimental and computational investigation of these methylene-modified polymers provides insight into their complex crystallization behavior and associated trends as well as the resulting changes in bulk material properties, including mechanical and thermal performance. Overall, the development of this new class of modified cyclobutane copolymers, together with an improved understanding of the relationship between the rotator phase and mechanical properties, provides a foundation for the future design of tunable, semicrystalline, and chemically recyclable poly­(cycloolefins).

## Supplementary Material



## Notes

General synthesis considerations and procedures for oligomers and polymers, supplementary reaction schemes, ^1^H NMR and ^13^C NMR spectroscopic analysis for all oligomers and polymers, MALDI-TOF characterization of cross-oligomers, experimental characterization of polymers including TGA, DSC, WAXS, DMA, and Instron tensile testing, simulation details including additional XRD simulation results, order parameter, ACF, and MSD simulations (PDF)
