# Supplementary material for: Iron-Catalyzed Cross-[2 + 2] Cycloaddition of Butadiene and α,ω-Dienes for Ductile and Chemically Recyclable Poly(oligocyclobutanes)
Source: J Am Chem Soc. 2026 Apr 10;148(15):15983–95. doi: 10.1021/jacs.5c23398 (PMC13107453; doi:10.1021/jacs.5c23398)
Supplement: Supplementary file 1 [file ja5c23398_si_001.pdf]

**Supplementary Information**

# Iron-catalyzed cross [2+2]-cycloaddition of butadiene and α,ω-dienes for ductile and chemically recyclable poly(oligocyclobutanes)

Cherish Nie,[a,‡] Sahana V. Sundar,[b,‡] Hang Zhang,[a] Richard A. Register,[b,c] Michael A. Webb,[b] Rodney D. Priestley,[b,c] Emily C. Davidson,[b,*] Paul J. Chirik[a,*]

[a]Department of Chemistry, Princeton University, Princeton, NJ, 08544, United States

[b]Department of Chemical and Biological Engineering, Princeton University, Princeton, NJ, 08544, United States

[c]Princeton Materials Institute, Princeton University, Princeton, NJ, 08544, United States

*Correspondence: pchirik@princeton.edu, edavidson@princeton.edu

## Table of Contents

S1. Materials and Methods .......... 3
S1.1 General Considerations and Procedures .......... 3
General Procedure for the [2+2] Cycloaddition-Oligomerization of α,ω-Dienes. .......... 3
General Procedure for the ADMET Polymerization of [2+2] Cross-Oligomers. .......... 5
S1.2 Nuclear Magnetic Resonance Spectroscopy .......... 6
S1.3 High-Temperature Gel Permeation Chromatography .......... 6
S1.5 Thermogravimetric Analysis .......... 6
S1.6 Differential Scanning Calorimetry .......... 7
S1.7 Wide-Angle and Small-Angle X-ray Scattering .......... 7
S1.8 Dynamic Mechanical Analysis .......... 8
S1.9 Tensile Testing .......... 8
S1.10 Molecular Dynamics Simulation .......... 9
S2. Investigation of the Limitations of Cross-[2+2] Cycloaddition .......... 9
S3. Additional Reaction Schemes .......... 11
S4. Spectroscopic Data .......... 13
S4.1. Oligomer NMR .......... 13
S4.2. Polymer NMR .......... 26
S5. Thermogravimetric Characterization .......... 33
S5.1. Oligomer TGA .......... 33
S5.2. Polymer TGA .......... 36
S6. Calorimetric Characterization .......... 38
S7. Experimental X-ray Scattering Measurements .......... 43
S7.1. Variable-temperature Wide-Angle X-ray Scattering .......... 43
S7.2 Tensile-stage Wide-Angle X-ray Scattering .......... 46
S8. Mechanical Characterization .......... 48
S8.1 Dynamic Mechanical Analysis .......... 48
S8.2 Tensile Testing .......... 49
S9. Simulation Details .......... 55
S9.1. Simulation Results of XRD .......... 56
S9.2 Simulation Results of Order Parameter .......... 57
S9.3 Simulation Results of ACF .......... 59
S9.4 Simulation Results of MSD .......... 61
References .......... 62

## S1. Materials and Methods

### S1.1 General Considerations and Procedures

All air- and moisture-sensitive manipulations were carried out using vacuum line, Schlenk and cannula techniques or in an MBraun inert atmosphere (nitrogen) dry box unless otherwise noted. All glassware was stored in a pre-heated oven prior to use. The solvents used for air- and moisture-sensitive manipulations were dried and deoxygenated using literature procedures.[1] 1,3-butadiene was purchased in reagent grade from Matheson, dried/deoxygenated over n-butyllithium, and stored in a thick-walled glass vessel containing activated 4-Å molecular sieves and sealed with a Teflon valve for at least 24 hours prior to use. Ethylene was purchased from Sigma Aldrich and dried and stored in a thick-walled glass vessel containing activated 4-Å molecular sieves and sealed with a Teflon valve for at least 24 hours prior to use. The ruthenium metathesis catalyst, M1002 was provided by Umicore. $[(^{Me}PDI)Fe(N_2)]_2(\mu_2\text{-}N_2)$[2] and $[(^{Me}(Et)PDI)Fe(N_2)]_2(\mu_2\text{-}N_2)$[3] were prepared according to literature procedures. 1,1,2,2-tetrachloroethane-$d_2$ (TCE-$d_2$) and chloroform-*d* were purchased from Cambridge Isotope Laboratories and dried over 4 Å molecular sieves. Benzene-$d_6$ used for air- or moisture-sensitive applications was distilled from sodium metal and stored over 4 Å molecular sieves under inert atmosphere. $H_2$ gas was purchased from Airgas® and passed through a column of alternating layers of 4 Å molecular sieves and MnO/Vermiculite before being introduced into the reaction vessel. Unless specified, all other reagents were purchased commercially and used as received without further purification.

<u>General Procedure for the [2+2] Cycloaddition-Oligomerization of α,ω-Dienes.</u>

This is a modified version of a previously reported literature procedure.[4] In a nitrogen-filled glovebox, an oven-dried, thick-walled Schlenk bomb equipped with a magnetic stir bar was charged with 0.1 mol % of $((^{Me}EtPDI)FeN_2)_2(\mu\text{-}N_2)$ (0.2 mol % [Fe]) relative to combined comonomer charge. Toluene (approximately 1 mL per 8 mmol of total monomers) was added if desired or omitted if reaction was run neat. The flask was sealed with a Teflon valve and brought out of the glovebox. The reaction vessel was frozen in liquid nitrogen and the headspace was evacuated on a high-vacuum line. The desired amount of butadiene was condensed into the reaction vessel by vacuum transfer using a calibrated gas bulb. The flask was sealed, and upon thawing to room temperature, the evolution of $N_2$ gas from the activation of the precatalyst was observed. Once $N_2$ evolution ceased, the resulting dark red reaction mixture was frozen again with liquid nitrogen and the headspace evacuated. The desired amount of diene comonomer was then added to the reaction vessel under conditions chosen depending on diene volatility: for highly volatile dienes (1,4-pentadiene), the diene comonomer was added by vacuum transfer from a separate, degassed Schlenk bomb containing an excess of the diene comonomer using a calibrated gas bulb; for less volatile dienes (1,5-hexadiene and 1,7-octadiene), the desired mass of diene comonomer was added to a separate Schlenk bomb in the glovebox, degassed, and transferred by direct vacuum transfer. *Note: butadiene must be added before the α,ω-diene comonomer and the butadiene:α,ω-diene ratio must be greater than 2:1 to avoid undesirable side reactions such as*

*isomerization of the diene comonomer.* The reaction was thawed to room temperature and stirred at 50 °C for 48 hours. The reaction was observed to become more viscous over time. Aliquots of the reaction were taken in the glovebox at the end of the reaction time to confirm complete conversion of all comonomers to oligomers by $^{1}$H NMR spectroscopy. The reaction was then quenched by opening the flask to air and the reaction mixture was redissolved in room temperature or boiling hexanes depending on the solubility of the oligomers. The hexanes solution was filtered through a silica plug to remove catalyst residue. The flask and silica plug were washed with additional hexanes (room temperature or heated) up to three times. The filtrate was concentrated under reduced pressure and dried under vacuum to afford the desired [2+2] cycloaddition oligomer as a colorless semisolid. For the cross-cycloaddition of 10 equiv. butadiene and 1 equiv. 1,4-pentadiene, due to the insolubility of the resulting cross-oligomer, the oligomer was precipitated into methanol and isolated by filtration. The $M_n$ was determined by total proton integration relative to unsaturated end-groups by $^{1}$H NMR spectroscopy.

*Representative Synthesis of B4O1.*

Prepared according to the general procedure with 37 mg (0.038 mmol, 0.1 mol % of total monomers) of $((^{Me}EtPDI)FeN_2)_2(\mu\text{-}N_2)$, 5 mL toluene, 1.64 g (30.4 mmol, 6 equiv) of butadiene, and 0.838 g (7.60 mmol, 1 equiv) of 1,7-octadiene. Complete conversion of comonomers was confirmed by $^{1}$H NMR spectroscopy after 48 hours, and workup with room temperature hexanes gave an isolated yield of 2.44 g (98%) of B4O1 as a colorless semisolid.

**Table S1. Summary of Synthetic Data for [2+2]-Cross Oligomers.**

| **Oligomer** | **Yield** (%) | $M_n$ (g/mol)[a] | **Physical Appearance** |
|---|---|---|---|
| B2P1 | 64[b] | 284 | Colorless oil |
| B3P1 | 98 | 260 | Colorless semisolid |
| B3P2 | >99 | 299 | Colorless semisolid |
| B4P1 | >99 | 317 | Colorless semisolid |
| B6P1 | >99 | 376 | Colorless semisolid |
| B10P1 | 67[c] | 587 | Colorless powder |
| B4H1 | 99 | 326 | Colorless semisolid |
| B6H1 | >99 | 380 | Colorless semisolid |
| B4O1 | 98 | 245 | Colorless semisolid |
| B6O1 | >99 | 408 | Colorless semisolid |

[a] Determined by total proton integration relative to end groups by $^{1}$H NMR spectroscopy based on an average oligomer composition of *n* equiv butadiene and 1 equiv *α,ω*-diene. [b] Reduced isolated

yield due to moderate volatility. [c] Reduced isolated yield due to workup by filtration and loss of the soluble fraction.

General Procedure for the ADMET Polymerization of [2+2] Cross-Oligomers.

Adapted according to a previously reported procedure:[4] To an oven-dried 100mL two-necked round bottom flask equipped with a magnetic stir bar and reflux condenser was added 2.0 g of cross oligomer, benzoquinone (5.0 mol % relative to cross oligomer), and xylenes (10 mL/g). The mixture was stirred at 60°C to fully dissolve the oligomer and deoxygenated by bubbling with argon for 20 minutes. In a nitrogen-filled glovebox, M1002 (0.5 mol % relative to cross oligomer) was dissolved in minimum dry toluene, then removed from the glovebox and added to the reaction flask via syringe. The argon flow was reduced to a gentle flow to minimize solvent loss. The temperature was increased to the desired temperature (100–140 ºC) which was sufficient to maintain polymer solubility and stirred for 48 to 72 hours, with periodic addition of degassed solvent if significant evaporation was observed. The reaction was diluted with xylenes and quenched with 2 mL ethyl vinyl ether diluted with 10 mL xylenes. The polymer was then precipitated into acidic methanol, isolated via filtration, and washed with methanol, acetone, and hexanes to yield an off-white powder which was dried under vacuum. The material was characterized by $^{1}H$ and $^{13}C\{^{1}H\}$ NMR (TCE-$d_2$; 140°C) spectroscopy. End-group analysis was used to determine degree of polymerization, and high-temperature GPC was used to measure the molecular weight distribution where possible.

*Representative Synthesis of pB4H1*

Prepared according to the general procedure with 2.0 g (6.7 mmol) of B4H1, 36 mg (0.335 mmol, 5 mol% relative to oligomer) of benzoquinone, 22 mg (0.034 mmol, 0.5 mol % relative to oligomer) of M1002, and 20 mL of xylenes at a reaction temperature of 120 ºC for 72 hours to yield 1.657 g (92%) of pB4H1 as a flaky, colorless solid. The material was characterized by $^{1}H$ and $^{13}C\{^{1}H\}$ NMR (TCE-$d_2$, 140°C) spectroscopy and HT-GPC (TCB, 145 °C, polyethylene calibration).

**Table S2. Summary of Synthetic Data for ADMET Polymers.**

| **Polymer** | **m** | $T_{synth}$ (ºC) | **Yield** (%) | $M_n$ (kg/mol)[a] | $M_n$ (kg/mol)[b] | Đ[b] |
|---|---|---|---|---|---|---|
| pB3P1 | 496 | 100 | 87 | 100.4 | [c] | [c] |
| pB3P2 | 263 | 120 | 97 | 67.5 | 12.5 | 2.54 |
| pB4P1 | 225 | 120 | 98 | 57.7 | [c] | [c] |

| | | | | | | |
|---|---|---|---|---|---|---|
| pB6P1 | 90 | 140 | 79 | 32.8 | 11.0 | 2.65 |
| pB4H1 | 134 | 120 | 92 | 36.3 | 12.8 | 2.42 |
| pB6H1 | 113 | 140 | 91 | 42.8 | 11.0 | 2.64 |
| pB4O1 | 166 | 120 | 84 | 49.6 | [c] | [c] |
| pB6O1 | 127 | 140 | 91 | 51.7 | [c] | [c] |

[a] Determined by high-temperature $^1H$ NMR spectroscopy in TCE-$d_2$ at 140 °C. [b] Determined by HT-GPC in TCB at 145 °C relative to polyethylene. [c] HT-GPC not available.

### S1.2 Nuclear Magnetic Resonance Spectroscopy

$^1H$ NMR spectra were recorded on either Bruker AVANCE 400 or 500 spectrophotometers operating at 399.80 MHz, and 500.46 MHz, respectively. $^{13}C$ NMR spectra were recorded on either Bruker AVANCE 400 or 500 spectrometers operating at 100.54 MHz and 125.85 MHz, respectively. All $^1H$ and $^{13}C$ NMR (**Section S4, Figures S1-S30**) chemical shifts are reported in ppm relative to $SiMe_4$ using the $^1H$ (chloroform-*d*: 7.26 ppm; 1,1,2,2-tetrachloroethane-$d_2$: 6.00 ppm) and $^{13}C$ (chloroform-*d*: 77.16 ppm; 1,1,2,2-tetrachloroethane-$d_2$: 73.78 ppm) chemical shifts of the solvent as a standard. $^1H$ NMR data for diamagnetic compounds are reported as follows: chemical shift, multiplicity (s = singlet, d = doublet, t = triplet, q = quartet, p = pentet, br = broad, m = multiplet, app = apparent, obs = obscured), coupling constants (Hz), integration, assignment.

### S1.3 High-Temperature Gel Permeation Chromatography

High-Temperature Gel Permeation Chromatography (HT-GPC) was conducted using a Polymer Laboratories 220 GPC system. The system used three Polymer Laboratories PLgel 10 μm Mixed-B LS columns with 1,2,4 trichlorobenzene at 145 °C as the mobile phase, a 325 μL injection volume, and a 1.0 mL/min flow rate. Elution times were calibrated against polyethylene.

### S1.4 Matrix-Assisted Laser Desorption/Ionization Time-of-Flight

Matrix-Assisted Laser Desorption/Ionization Time-of-Flight (MALDI-TOF) mass spectrometry data were acquired in positive ion and reflection mode on a Bruker ultrafleXtreme MALDI TOF/TOF system equipped with a solid-state Smartbeam II laser operating at 355 nm; m/z was calibrated with red phosphorous clusters (spanning m/z = 60−600 g/mol). Resulting spectra were analyzed using Polymerix. Sample preparation followed a layered spotting procedure onto a Bruker stainless steel plate in the following order: 0.2 μL of a solution of matrix (2,5-dihydrobenzoic acid or retinoic acid) in THF (10 mg/mL), 0.1 μL of a saturated solution of silver nitrate in ethanol, and 0.2 μL of a solution of DVOCB in THF or pentane (10 mg/mL), allowing each layer to air-dry before spotting the next layer.

### S1.5 Thermogravimetric Analysis

To prepare samples for TGA, approximately 2 – 10 mg of material was loaded into 100 μL platinum sample pans. Depending on the experiment, the samples were run under air or nitrogen gas (**Figures S31-S38**) at a constant flow rate of 60 mL/min using a TA Instruments TGA Q50. An increase in mass was observed prior to degradation while heating under air due to oxidation. During each experiment, the samples were heated from ambient to 105 °C at 20 °C/min, held isothermally for a minimum of 10 minutes to remove any adsorbed water or residual solvent, and then heated to 550 °C at a rate of 20 °C/min.

### S1.6 Differential Scanning Calorimetry

Differential scanning calorimetry (DSC) samples were prepared by loading approximately 3 – 10 mg of material into aluminum Tzero Pans before crimp sealing the pan with an aluminum lid. A pin hole was made in the center of the top of the crimped DSC sample to ensure samples were run under inert conditions. For all standard DSC traces (**Figures S39-S43**), at least two heating/cooling cycles were performed at a heating/cooling rate of 5 °C/min. For modulated DSC (MDSC) measurements (**Figure S44**), at least two heating/cooling cycles were performed, and data was analyzed from the second heating to eliminate any thermal history effects. An underlying heating rate, modulation amplitude, and modulation period of 2 °C/min, ±1.272 °C, and 60.0 s, respectively, were used to improve signal for even slight changes in heat capacity. The temperature assigned to each thermal transition was obtained from the peaks of DSC curves from the second heating to control the thermal history. All DSC samples were run using a TA Instruments DSC2500 and data analysis was performed with TA Instruments TRIOS software.

### S1.7 Wide-Angle and Small-Angle X-ray Scattering

Wide-angle and small-angle X-ray scattering (WAXS and SAXS) measurements were performed on a Xenocs Xeuss 3.0 in-lab beamline equipped with a Cu Kα source and 2D detector system (Dectris Eiger 2R 1M-pixel 2D). For SAXS measurements, the distance between the detector and the sample was calibrated using a silver behenate (AgBeh) standard sample under ESAXS high-resolution beam conditions (extra small-angle X-ray scattering, with a sample-detector distance of 1100 mm). SAXS measurements (**Figure S49**) were each taken for 300 s with samples mounted on the standard stage. For WAXS measurements, the sample-detector distance was calibrated using a lanthanum hexaboride ($LaB_6$, simple cubic)[5] standard under WAXS high-resolution beam conditions. Variable-temperature WAXS data were fitted to a convolution of Gaussian peaks to extract crystalline peak locations (example shown in **Figure S45**).

Samples for X-ray scattering measurements were prepared by loading polymer powder into a steel washer approximately 0.5 mm thick with a 5.0 mm inner diameter. Using a Carver melt-press, the samples were heated above their melting temperature (based on DSC results) and compressed to form a disk-shaped specimen. To limit thermally induced oxidation during processing, the washer was sandwiched between Kapton sheets. For variable-temperature WAXS measurements, the polymer disk was then punched out of the washer using a steel rod and loaded into a Linkam heating cell, ensuring good thermal contact. Prior to data collection, samples were heat cycled to

above their melting temperature and then cooled at 5 °C/min to match DSC cooling conditions and eliminate processing effects on crystallinity. Finally, raw two-dimensional (2D) scattering images were collected either at room temperature or on heating with a collection time of ten minutes per sample and a beam size of 0.3 mm × 0.3 mm, and then azimuthally integrated into one-dimensional (1D) patterns for further analysis. For all variable-temperature X-ray scattering measurements (**Figures S46-S48**), an in-house temperature calibration was applied to the data.[6]

Tensile-stage WAXS and SAXS measurements were taken using melt-pressed polymer bars loaded into the Linkam Modular Force Stage with a 200 N load cell. To ensure necking first initiates in the path of the X-ray beam, each sample was slightly notched using a circular punch to create a narrower region in the center of the bar that was aligned with the X-ray beam path (**Figure S50**). Measurements were taken at room-temperature and at 60 °C during tensile elongation (**Figure S51**).

### S1.8 Dynamic Mechanical Analysis

Samples for mechanical testing were prepared by melt-pressing polymer powder in Teflon molds sandwiched between Kapton sheets to minimize oxidation during processing. Molds were then loaded into a Carver melt-press at temperatures above the melting temperature (as determined from DSC) while applying minimal pressure. After melt-pressing, molds were removed from the press and quenched to room temperature through contact with a room-temperature metal block before removing the solid polymer bars from the Teflon. The resulting bars were approximately 3 mm wide x 20 mm long x 0.5 mm thick.

Polymer bar samples were then loaded into a PerkinElmer DMA 8000 in tension mode. Temperature sweep experiments were run at a frequency of 1 Hz and a heating rate of 3 °C/min (**Figure S52**). Frequency multiplex experiments were run at a range of frequencies between 0.1 and 40 Hz, where data was collected at 4 °C intervals with a heating rate of 3 °C/min.

### S1.9 Tensile Testing

Samples for mechanical testing were prepared by loading polymer powder in metal shims (approximately 0.4 mm thick with a 20 mm inner diameter) sandwiched between Kapton sheets, before melt-pressing above polymer melting temperature to form a solid disk. The polymer disk was then removed from the shim and cut into approximately 2 mm wide strips using a razor blade (sample preparation schematic shown in **Figure S53**). To minimize clamping effects, the ends of the strips were then glued into 3D printed resin pucks printed on a Form 4 SLA printer using clear resin. Uniaxial tensile testing was then performed using an Instron 5865 with a 500 N load cell, using the Instron clamps to grip the resin pucks on either end of the sample. Tensile tests were conducted at a strain rate of 3% engineering strain per minute unless otherwise specified to ensure consistency with previous tensile testing of pDVOCB and pDVOCB-related polymers.[4,7] At least three replicates were run to ensure consistency in data (**Fig. S54, S56-S57**). Toughness values were calculated as the area underneath the stress-strain curves (**Table S4, Fig. S60**) and Young's

modulus values were calculated from the initial slope in the linear elastic region (**Table S5**, **Fig. S61**).

### S1.10 Molecular Dynamics Simulation

All simulations were performed with version 3 Mar 2020 of LAMMPS simulation.[8] Periodic boundary conditions were used, and the long-range electrostatic interactions were handled with PPPM Algorithm[9] with a 14 Å cutoff. All simulations were in NPT ensemble with 1 fs timestep, and Nosé-Hoover[10] thermostat and barostat were used. The pB4X1 systems were described with a force field based on an adaptation of TAFFI framework.[6,11] More details about system preparation and production can be found in **Section S9**.

## S2. Investigation of the Limitations of Cross-[2+2] Cycloaddition

The exclusive formation of oligomers in these cross-[2+2] cycloaddition reactions prompted investigation into why [2+2] cycloaddition does not occur between oligomer chains to form higher molecular weight polymers. The [2+2] cycloaddition of terminal alkenes has been demonstrated to be inhibited by steric bulk near the terminal alkene;[12] thus, oligomers whose chain ends consist primarily of vinyl groups are likely sterically inhibited from engaging in [2+2] cycloaddition. An additional complication is that vinylcyclobutyl chain ends also may undergo ring-opening oxidative addition and retro-[2+2][13,14]which are presumed to outcompete alkene-alkene [2+2] cycloaddition between two oligomer chains, as the [2+2] homodimerization of vinylcyclobutane (VCB) has never been observed (**Scheme S1A**).[13,15,16] To deconvolute the steric effects of VCB from its propensity for ring-opening, 3-methyl-1-pentene (3M1P) was chosen as a noncyclic but similarly substituted vinyl olefin for evaluating [2+2] cycloaddition reactivity (**Scheme S1B**). The homodimerization was first evaluated by adding 3M1P to a benzene-$d_6$ solution containing 5 mol% of ((MeEtPDI)FeN2)2(μ-N2) (10 mol% total [Fe]). At room temperature, slow isomerization to 3-methyl-2-pentene (3M2P) was observed. When heated at 50 °C for 16 hours to replicate oligomerization conditions, 3M1P underwent complete isomerization to 3M2P. This outcome suggests that terminal olefins with substituents at the 3-position such as VCB are too sterically hindered to engage in [2+2] homodimerization regardless of the possibility of competing ring-opening. Next, the cross cycloaddition between a sterically hindered and sterically accessible alkene was evaluated by exposing a neat 5:1 mixture of 3M1P and 1-hexene to 5 mol% of ((MeEtPDI)FeN2)2(μ-N2) (10 mol% total [Fe]) at room temperature for 48 hours. Filtration through a plug of silica with pentane and removal of volatiles yielded an approximately 50:50 mixture of the cross [2+2] cycloaddition product trans-1-(sec-butyl)-2-butylcyclobutane and the homodimer trans-1,2-dibutylcyclobutane. While the presence of the cross-product confirmed that [2+2] cycloaddition is possible when only one of the two coupling partners is sterically hindered, the disproportionally high fraction of the homodimer despite the use of excess of 3M1P in the reaction indicated that [2+2] cycloaddition involving bulky alkenes is highly unfavorable. These

results support that alkene-alkene [2+2] cycloaddition is unlikely to occur between oligomers, thus restricting the cross-[2+2] strategy to the formation of oligomers rather than polymers.

Additionally, the excess of butadiene in the reaction coupled with the strong preference of the iron catalyst to be a butadiene complex rather than an alkene or bis(alkene) complex makes it highly unlikely to be able to facilitate an alkenyl [2+2] reaction under the oligomerization reaction conditions (with the exception of pentadiene which is undergoing further investigation for future publication). As the active catalyst is the iron butadiene complex, oligomers grow through a chain-growth processes by the addition of butadiene monomers to chain ends rather than by the step-growth combination of oligomer chains (see Scheme S2). As such, oligomerization can and does proceed through the dimerization of butadiene which is likely to lead to the formation of the observed amounts of DVOCB in all cross-oligomerization product mixtures.

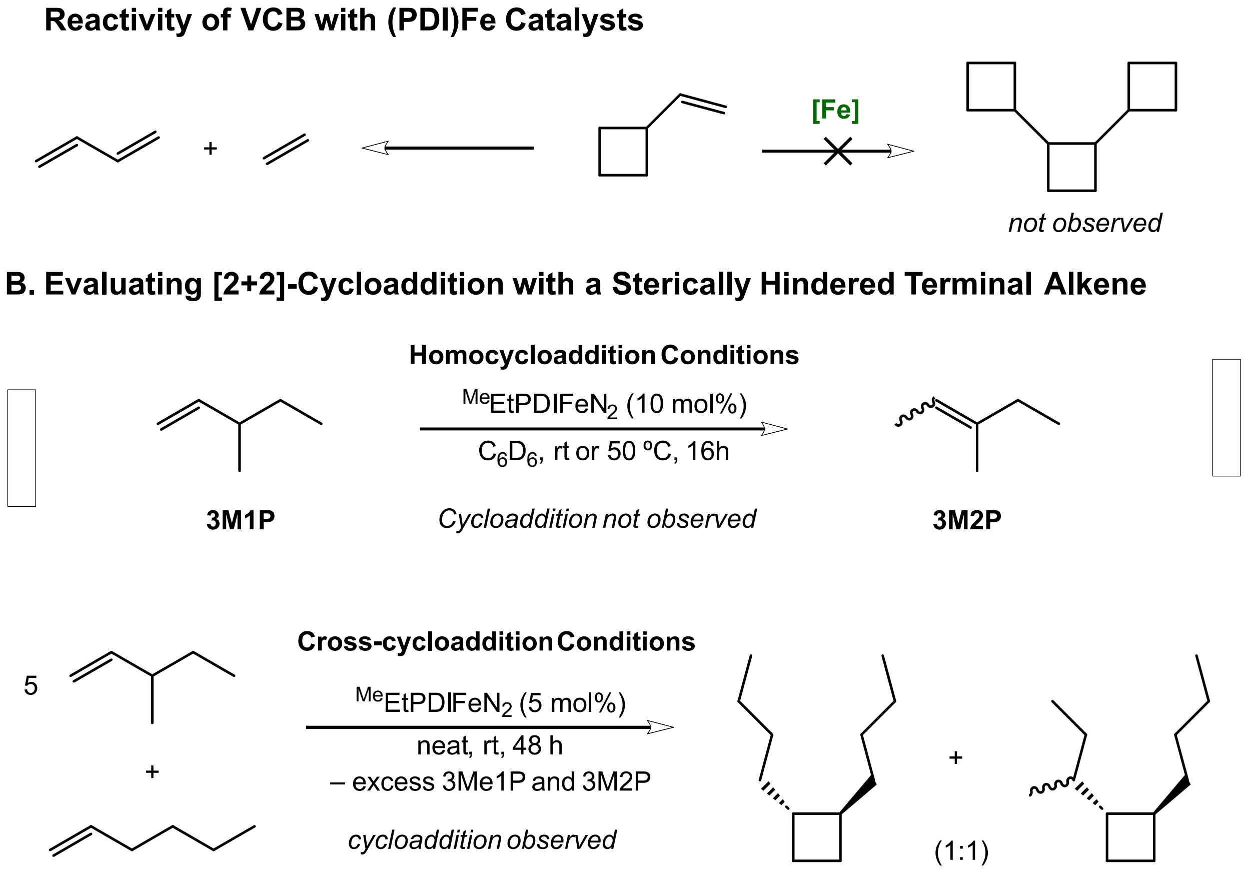


**Scheme S1.** Investigation of Iron-Catalyzed Cross-[2+2] with Hindered Alkenes.

## S3. Additional Reaction Schemes

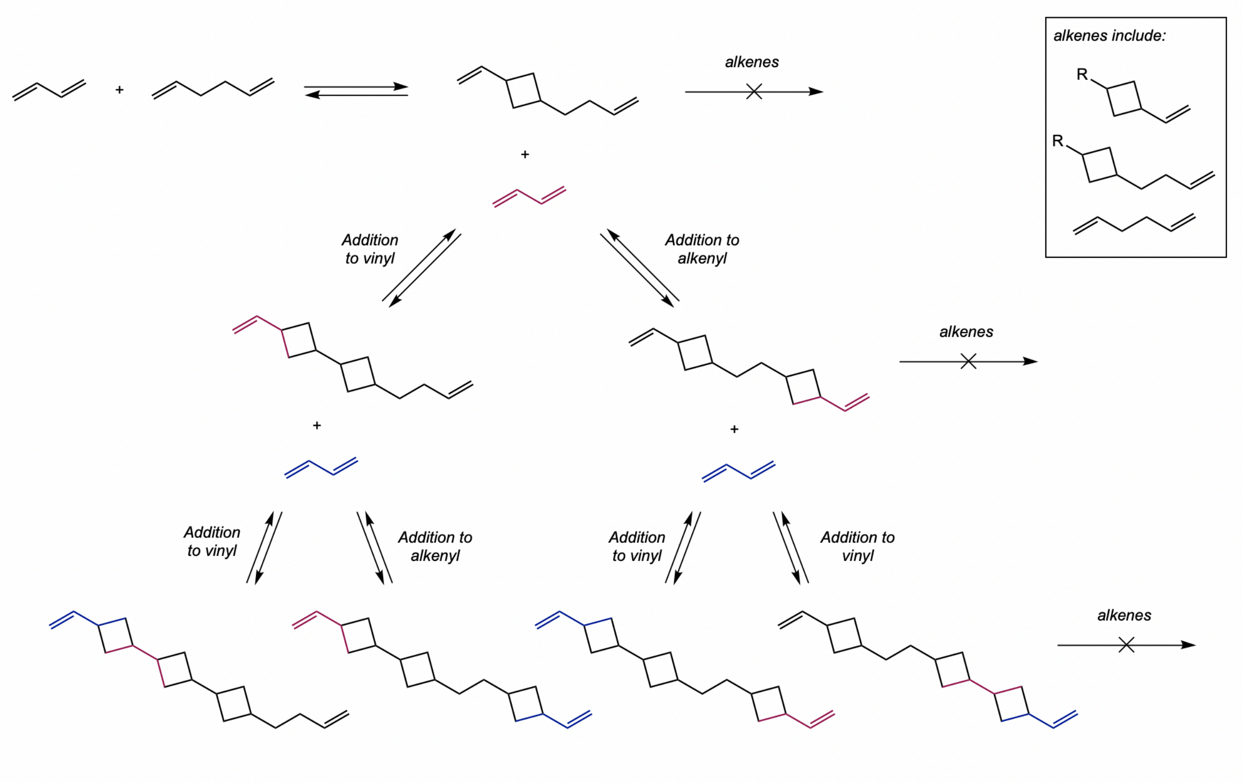


**Scheme S2.** Cross-oligomerization process between BD and α,ω-diene (HD shown here). After the reaction of BD and HD, the remaining excess BD can be added to either end of the chain in subsequent steps until it is consumed. The active catalyst for the cross-oligomerization reaction is a Fe(butadiene) complex, so chains primarily grow through the addition of butadiene to chain ends. Alkene-alkene addition is disfavored and is rarely observed (except for in the BD:PD case, leading to the S1 and S2 structures of B4P1 and B3P2), so each oligomer has an average incorporation of 1 α,ω-diene and *n* BD for a starting ratio of *n*:1 BD:XD. The addition of butadiene to chain ends can result in a distribution of structures with different placement of the methylene spacers from the α,ω-diene, as shown.

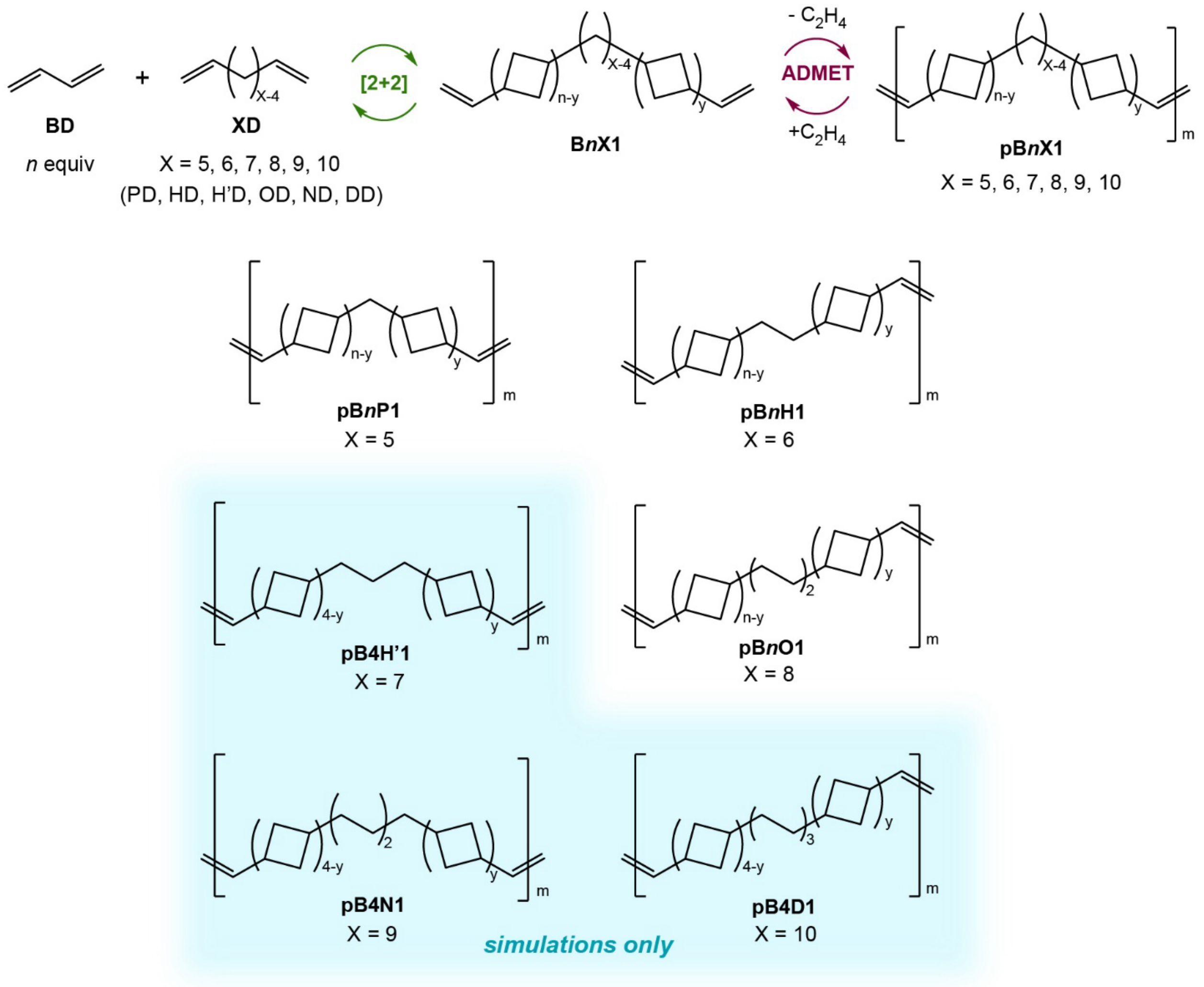


**Scheme S3.** Representative [2+2] cycloaddition and ADMET polymerization for a range of α,ω-diene comonomers. Cross-oligomers B*n*X1 and copolymers pB*n*X1 were synthesized using pentadiene (PD, X = 5), hexadiene (HD, X = 6), and octadiene (OD, X = 8). Simulated polymer structures were investigated for pBnX1 for the six structures shown (X = 5 – 10), including the three synthesized polymers as well as pBnH'1, pBnN1, and pBnD1 which were not synthesized (cyan highlight).

## S4. Spectroscopic Data

### S4.1. Oligomer NMR Data

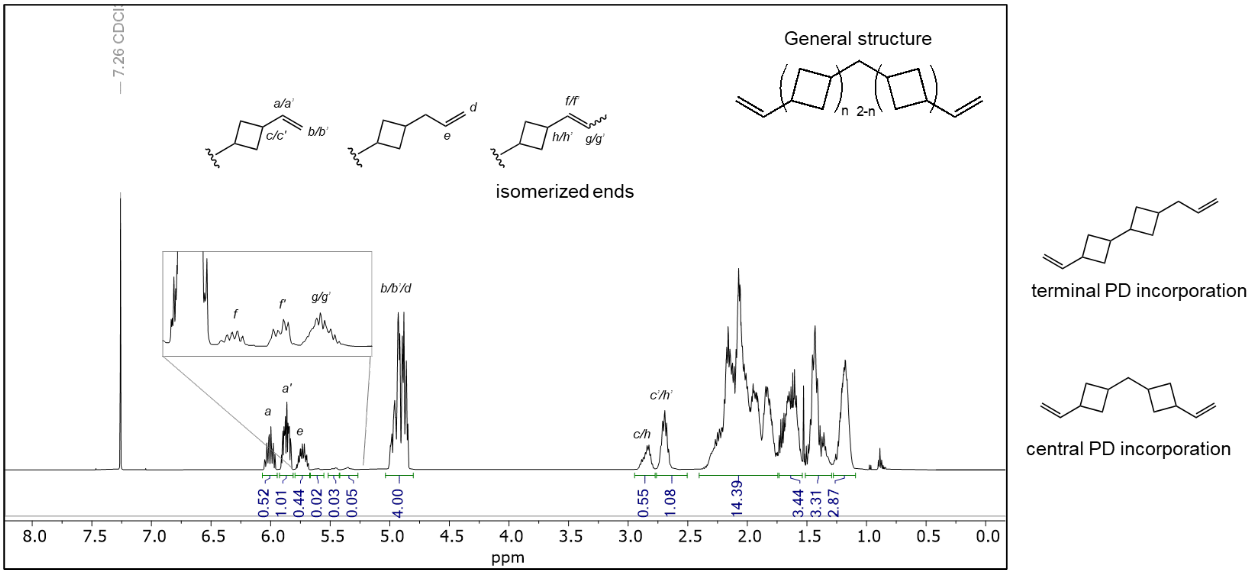


**Figure S1.** Representative $^{1}$H NMR spectrum of the B2P1 oligomer, displaying signals from isomerized ends. $^{1}$H NMR (500 MHz, chloroform-*d*, 23 ºC) δ 6.07 – 5.95 (m, 0.5H), 5.87 (tdt, *J* = 12.3, 6.2, 2.9 Hz, 1H), 5.73 (dddt, *J* = 14.6, 12.9, 10.0, 5.2 Hz, 0.4H), 5.61 (dt, *J* = 15.7, 8.1 Hz, 0H), 5.47 (dd, *J* = 15.2, 6.8 Hz, 0H), 5.35 (tp, *J* = 18.4, 6.2, 5.4 Hz, 0H), 5.02 – 4.82 (m, 4H), 2.93 – 2.77 (m, 0.6H), 2.77 – 2.51 (m, 1H), 2.38 – 1.76 (m, 14H), 1.76 – 1.54 (m, 3H), 1.51 – 1.29 (m, 3H), 1.29 – 1.10 (m, 3H).

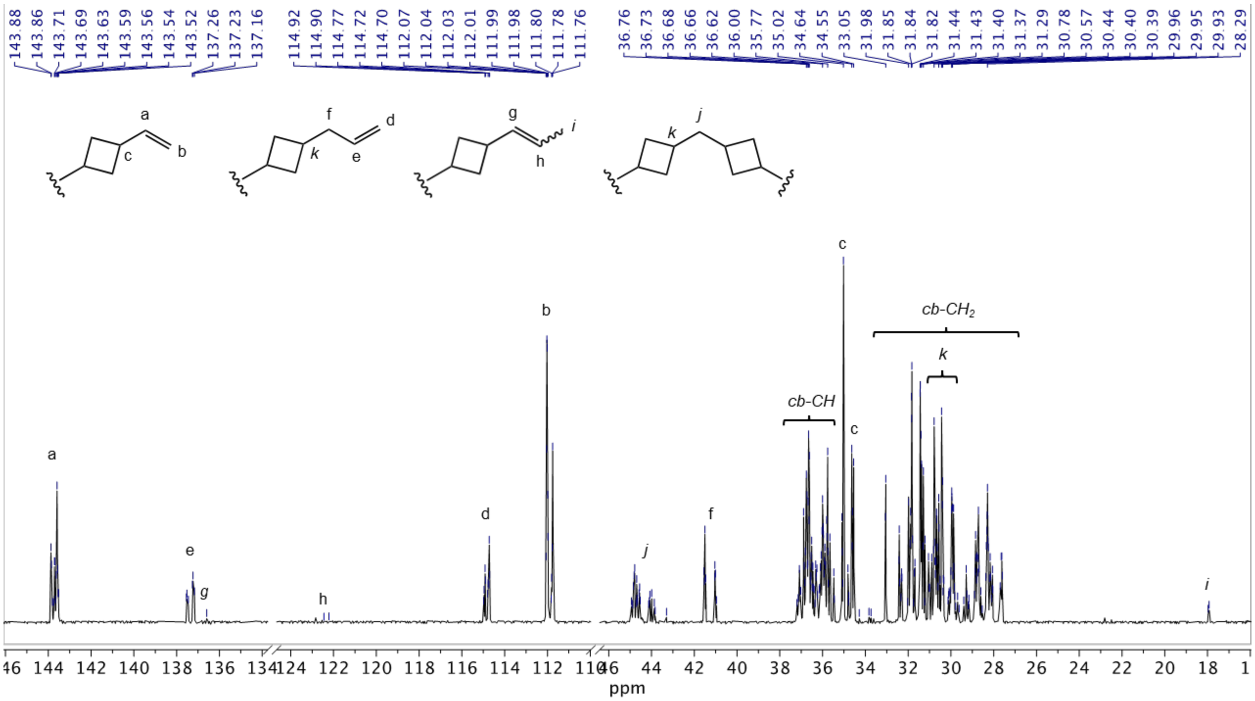


**Figure S2.** Representative $^{13}$C NMR spectrum for the B2P1 oligomer. $^{13}$C{$^{1}$H} NMR (126 MHz,

chloroform-*d*, 23 ºC) δ 143.88, 143.86, 143.81, 143.74, 143.71, 143.69, 143.66, 143.63, 143.59, 143.56, 143.54, 143.52, 137.54, 137.52, 137.45, 137.26, 137.23, 137.16, 136.82, 136.69, 136.59, 122.84, 122.46, 114.98, 114.96, 114.92, 114.90, 114.80, 114.77, 114.74, 114.72, 114.70, 112.07, 112.04, 112.03, 112.01, 111.99, 111.98, 111.83, 111.80, 111.78, 111.76, 44.94, 44.91, 44.88, 44.84, 44.81, 44.79, 44.72, 44.69, 44.68, 44.65, 44.58, 44.56, 44.53, 44.13, 44.10, 44.08, 43.99, 43.87, 43.83, 41.54, 41.53, 41.50, 41.47, 41.04, 41.03, 41.01, 40.97, 37.20, 37.14, 37.08, 37.04, 37.03, 36.89, 36.86, 36.83, 36.80, 36.76, 36.73, 36.71, 36.69, 36.68, 36.66, 36.63, 36.62, 36.57, 36.55, 36.53, 36.51, 36.50, 36.48, 36.45, 36.43, 36.40, 36.35, 36.32, 36.31, 36.29, 36.26, 36.24, 36.16, 36.14, 36.10, 36.06, 36.03, 36.00, 35.98, 35.96, 35.93, 35.91, 35.89, 35.87, 35.81, 35.79, 35.77, 35.75, 35.73, 35.66, 35.47, 35.45, 35.09, 35.08, 35.06, 35.02, 34.81, 34.79, 34.77, 34.64, 34.62, 34.60, 34.55, 33.06, 33.05, 32.43, 32.42, 32.40, 32.34, 32.32, 32.31, 32.29, 32.02, 31.98, 31.91, 31.89, 31.85, 31.84, 31.82, 31.80, 31.75, 31.72, 31.70, 31.68, 31.44, 31.43, 31.40, 31.37, 31.31, 31.29, 31.23, 31.21, 31.05, 31.01, 30.99, 30.89, 30.80, 30.78, 30.77, 30.75, 30.72, 30.70, 30.67, 30.66, 30.64, 30.60, 30.57, 30.55, 30.51, 30.44, 30.40, 30.39, 30.37, 30.35, 30.33, 30.13, 30.11, 30.09, 30.04, 30.03, 30.00, 29.97, 29.96, 29.95, 29.93, 29.90, 29.88, 29.86, 29.84, 29.82, 29.78, 29.68, 29.61, 29.40, 29.29, 29.26, 29.20, 29.18, 29.16, 28.90, 28.87, 28.85, 28.83, 28.79, 28.78, 28.75, 28.73, 28.71, 28.68, 28.60, 28.53, 28.37, 28.34, 28.31, 28.30, 28.29, 28.27, 28.25, 28.20, 28.16, 28.14, 28.09, 28.07, 28.06, 28.04, 27.70, 27.65, 27.62, 27.60, 22.81, 17.98, 17.93, 14.28, 14.21.

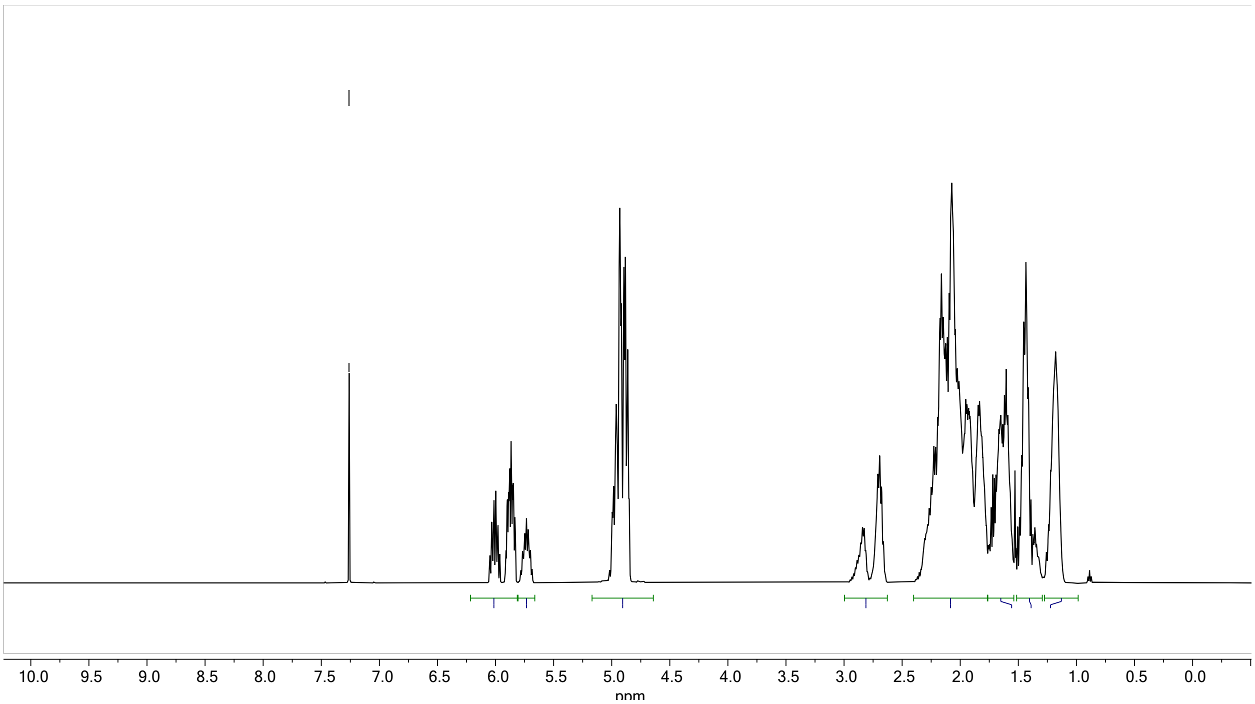


**Figure S3.** Representative $^{1}$H NMR spectrum for the B3P1 oligomer.

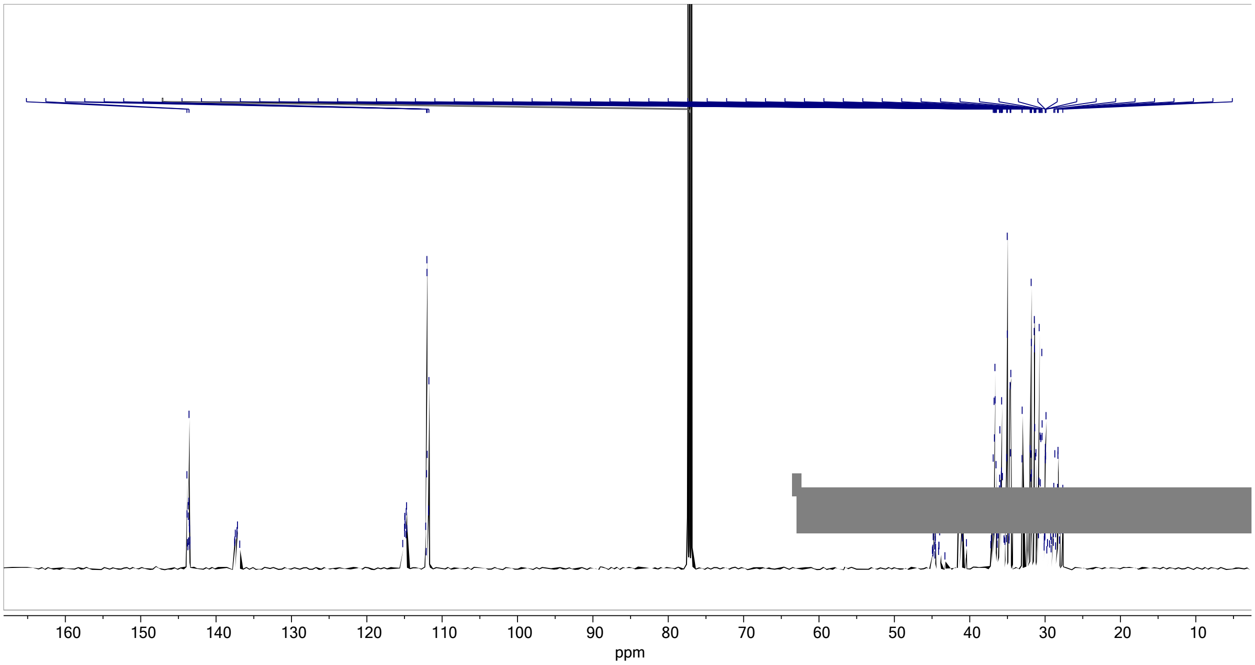


**Figure S4.** Representative $^{13}C$ NMR spectrum for the B3P1 oligomer. $^{13}C$ NMR (126 MHz, $CDCl_3$) δ 143.87, 143.85, 143.80, 143.74, 143.71, 143.68, 143.65, 143.62, 143.59, 143.56, 143.53, 143.52, 143.49, 143.48, 137.54, 137.52, 137.45, 137.25, 137.23, 137.16, 136.86, 115.23, 115.00, 114.98, 114.96, 114.92, 114.90, 114.80, 114.77, 114.72, 114.70, 112.19, 112.12, 112.07, 112.02, 112.01, 111.99, 111.81, 111.80, 111.76, 44.95, 44.91, 44.88, 44.84, 44.81, 44.78, 44.69, 44.65, 44.62, 44.58, 44.56, 44.53, 44.13, 44.10, 44.08, 43.99, 43.30, 41.54, 41.53, 41.50, 41.46, 41.38, 41.04, 41.03, 41.00, 40.96, 40.44, 37.20, 37.14, 37.10, 37.08, 37.04, 36.89, 36.86, 36.83, 36.80, 36.76, 36.73, 36.66, 36.62, 36.57, 36.55, 36.51, 36.45, 36.43, 36.40, 36.35, 36.32, 36.30, 36.29, 36.26, 36.24, 36.15, 36.14, 36.10, 36.06, 36.03, 36.00, 35.91, 35.89, 35.87, 35.81, 35.79, 35.76, 35.66, 35.47, 35.45, 35.33, 35.12, 35.09, 35.08, 35.06, 35.02, 35.01, 34.81, 34.79, 34.77, 34.64, 34.60, 34.55, 34.32, 33.06, 33.04, 32.43, 32.41, 32.40, 32.37, 32.32, 32.30, 32.29, 31.98, 31.90, 31.89, 31.85, 31.82, 31.80, 31.72, 31.70, 31.68, 31.44, 31.43, 31.40, 31.37, 31.31, 31.29, 31.21, 31.05, 31.01, 30.99, 30.89, 30.87, 30.80, 30.78, 30.75, 30.72, 30.70, 30.66, 30.64, 30.60, 30.57, 30.50, 30.44, 30.40, 30.39, 30.35, 30.33, 30.13, 30.11, 30.09, 30.04, 30.03, 30.00, 29.97, 29.96, 29.95, 29.93, 29.87, 29.83, 29.78, 29.68, 29.40, 29.28, 29.26, 29.20, 29.16, 28.91, 28.89, 28.87, 28.85, 28.83, 28.78, 28.75, 28.71, 28.68, 28.61, 28.60, 28.37, 28.34, 28.30, 28.29, 28.25, 28.20, 28.16, 28.14, 28.09, 28.06, 28.04, 27.72, 27.69, 27.65, 27.62, 27.60.

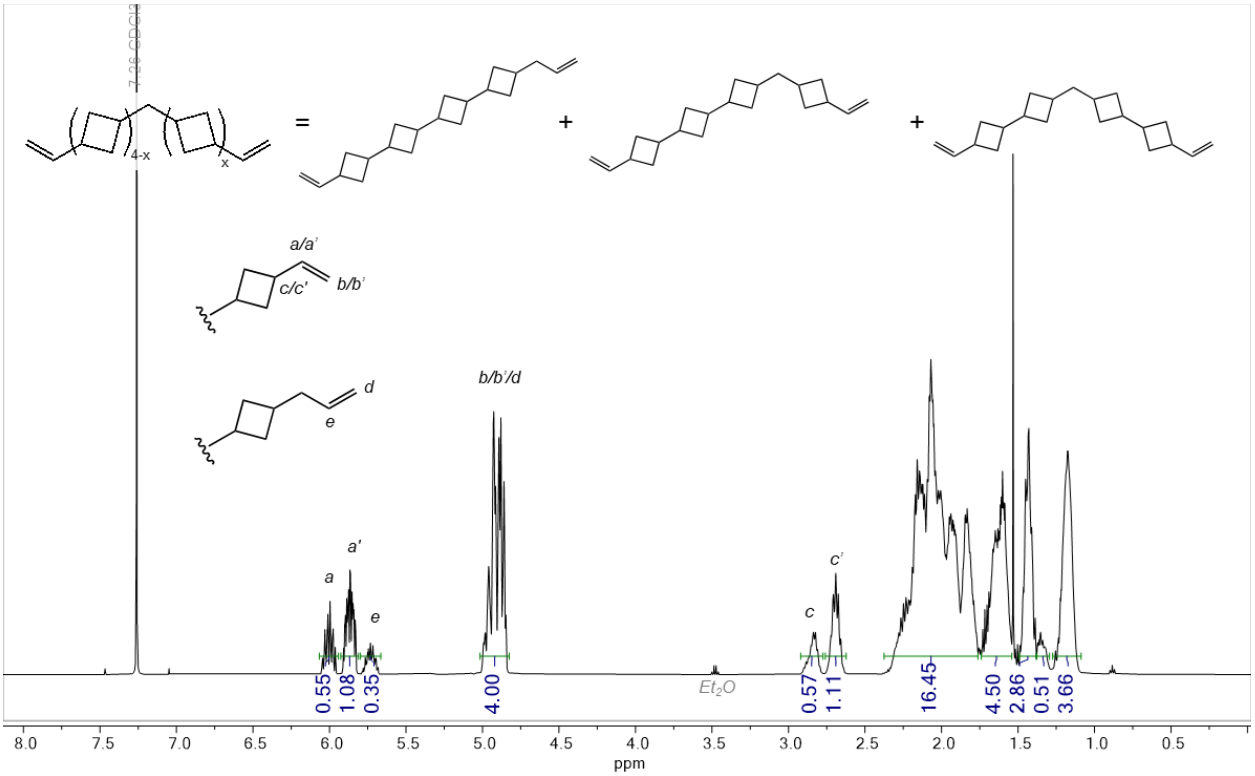


**Figure S5.** Representative $^{1}H$ NMR spectrum and annotated structures for the B4P1 oligomer.

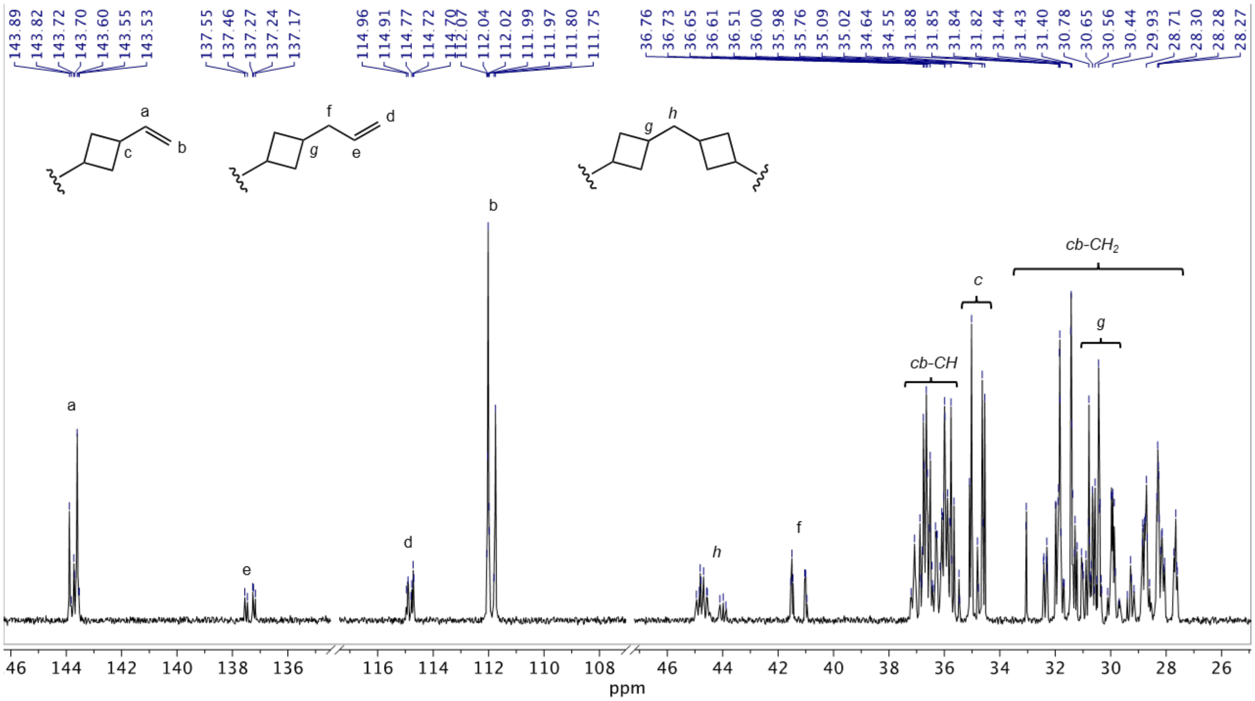


**Figure S6.** Representative $^{13}C$ NMR spectrum for the B4P1 oligomer.

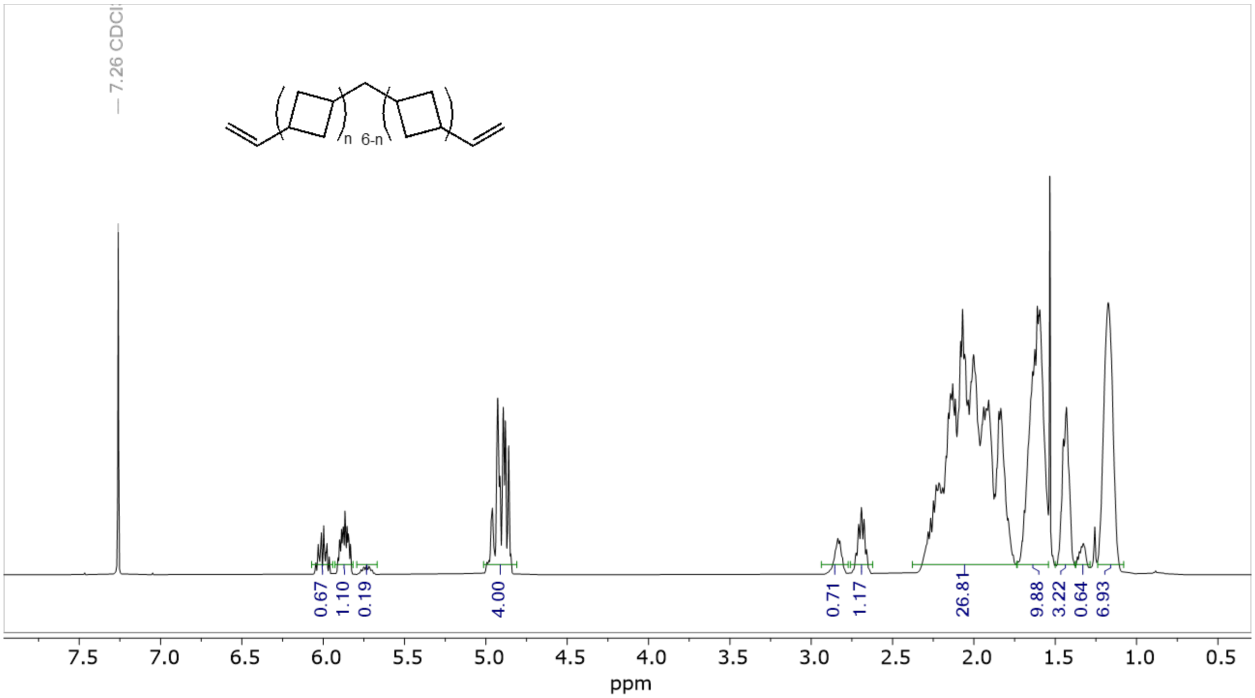


**Figure S7.** Representative $^{1}H$ NMR spectrum for the B6P1 oligomer. $^{1}H$ NMR (500 MHz, chloroform-*d*, 23 ºC) δ 6.00 (ddd, *J* = 17.0, 10.0, 6.9 Hz, 0.7H), 5.87 (ddd, *J* = 16.7, 10.5, 5.5 Hz, 1H), 5.73 (ddd, *J* = 19.4, 9.8, 6.8 Hz, 0.2H), 5.02 – 4.82 (m, 4H), 2.85 (dh, *J* = 14.7, 7.5 Hz, 0.7H), 2.69 (hept, *J* = 8.8 Hz, 1H), 2.35 – 1.74 (m, 27H), 1.74 – 1.54 (m, 10H), 1.50 – 1.37 (m, 3H), 1.34 (dt, *J* = 11.4, 5.9 Hz, 0.6H), 1.24 – 1.09 (m, 7H).

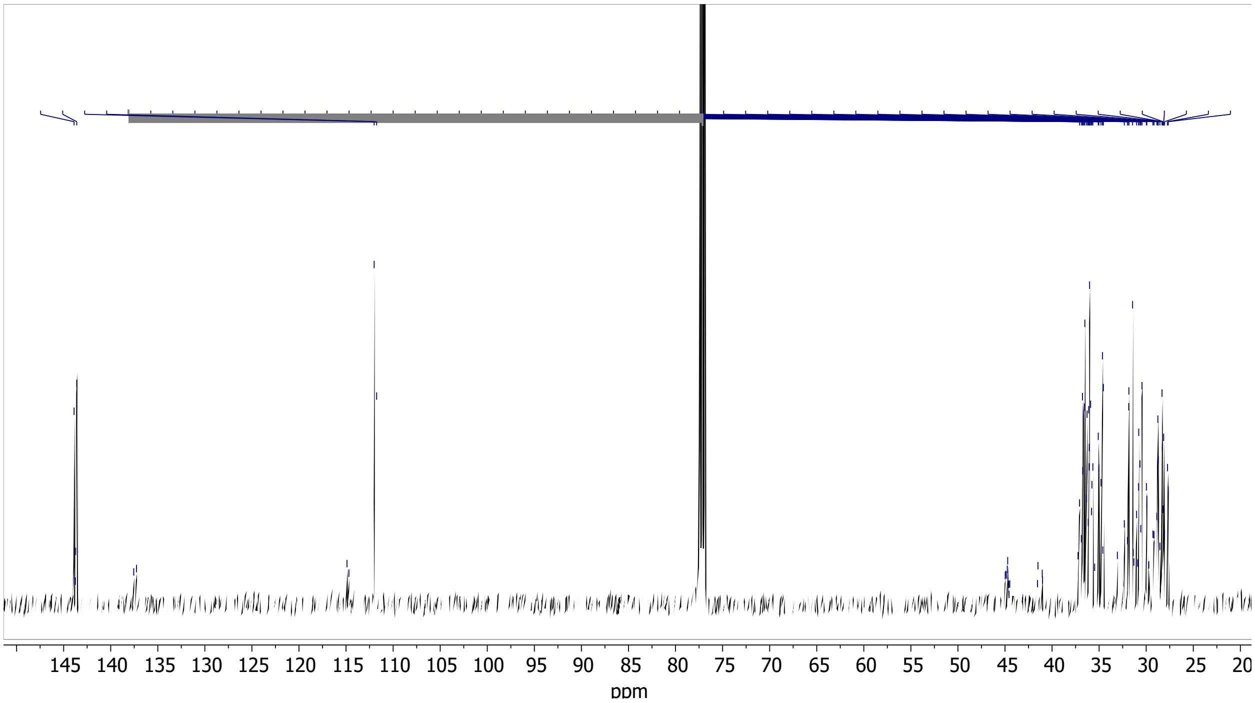


**Figure S8.** Representative $^{13}C$ NMR spectrum for the B6P1 oligomer. $^{13}C\{^{1}H\}$ NMR (126 MHz, chloroform-*d*, 23 ºC) δ 143.89, 143.76, 143.72, 143.61, 137.55, 137.27, 114.90, 114.70, 112.01, 111.75, 44.98, 44.95, 44.82, 44.76, 44.71, 44.60, 44.57, 44.50, 41.54, 41.50, 41.04, 41.02, 37.24, 37.08, 36.89, 36.77, 36.74, 36.67, 36.55, 36.52, 36.33, 36.28, 36.16,

36.11, 36.07, 36.04, 36.01, 35.91, 35.81, 35.77, 35.67, 35.48, 35.09, 35.03, 34.81, 34.64, 34.61, 34.55, 33.07, 32.32, 31.99, 31.86, 31.84, 31.45, 31.38, 31.31, 31.02, 30.90, 30.81, 30.79, 30.66, 30.58, 30.45, 29.98, 29.94, 29.72, 29.29, 29.17, 28.87, 28.77, 28.72, 28.55, 28.32, 28.28, 28.20, 28.15, 28.05, 27.73, 27.66.

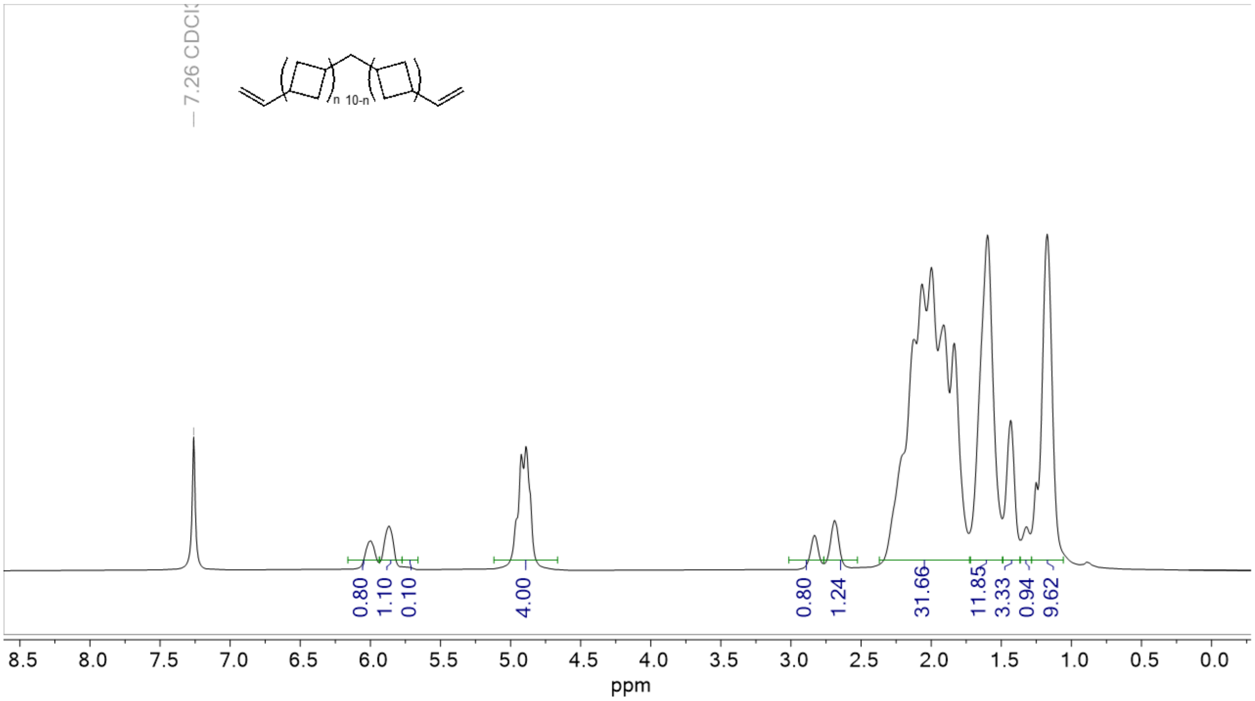


**Figure S9.** Representative $^1$H NMR spectrum for the B10P1 oligomer. $^1$H NMR (500 MHz, chloroform-*d*, 23 ºC) δ 6.11 – 5.94 (m, 0.8H), 5.94 – 5.78 (m, 1H), 5.78 – 5.66 (m, 0.1H), 5.08 – 4.69 (m, 4H), 2.96 – 2.77 (m, 0.8H), 2.77 – 2.56 (m, 1H), 2.35 – 1.73 (m, 32H), 1.72 – 1.49 (m, 12H), 1.49 – 1.37 (m, 3H), 1.37 – 1.29 (m, 1H), 1.29 – 1.00 (m, 10H).

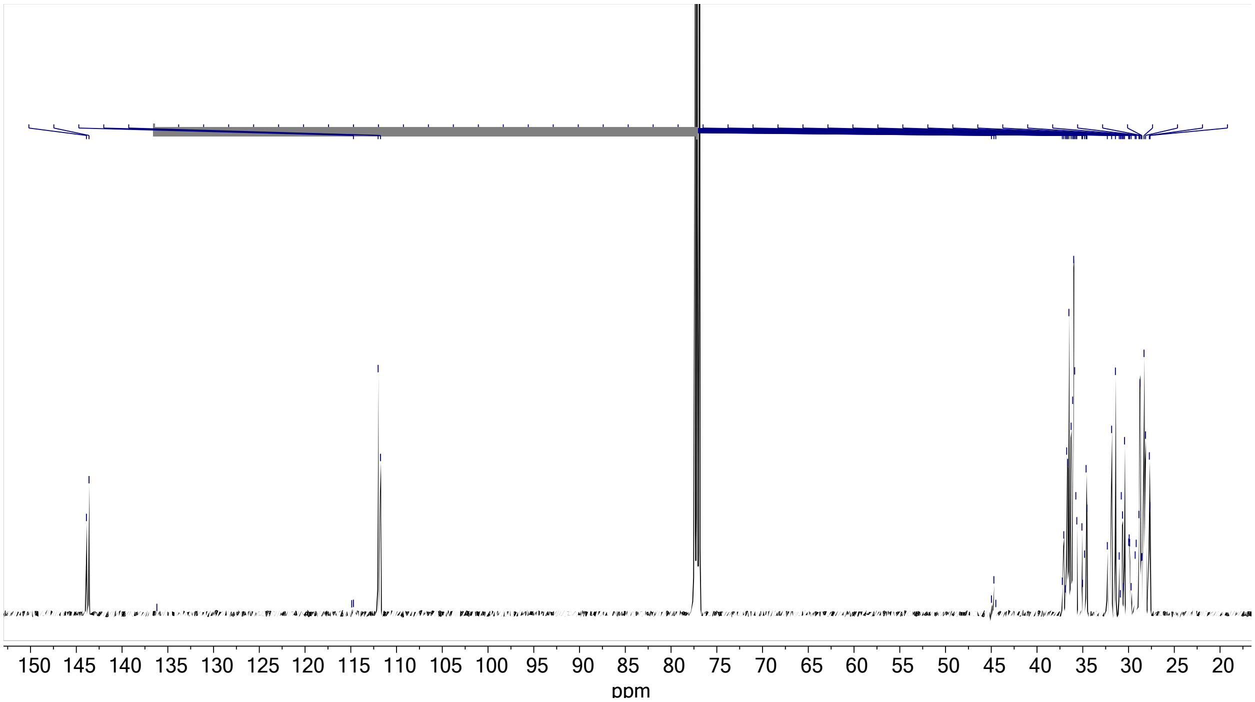


**Figure S10.** Representative $^{13}C$ NMR spectrum for the B10P1 oligomer. $^{13}C\{^{1}H\}$ NMR (126 MHz, chloroform-*d*, 23 ºC) δ 143.89, 143.61, 136.19, 114.90, 114.71, 112.01, 111.75, 44.98, 44.72, 44.51, 37.23, 37.08, 36.89, 36.76, 36.66, 36.52, 36.28, 36.10, 35.99, 35.89, 35.76, 35.66, 35.10, 35.03, 34.81, 34.64, 34.55, 32.32, 31.85, 31.44, 31.02, 30.90, 30.79, 30.66, 30.57, 30.44, 29.98, 29.93, 29.88, 29.71, 29.28, 29.16, 28.86, 28.74, 28.60, 28.54, 28.31, 28.14, 27.72, 27.65.

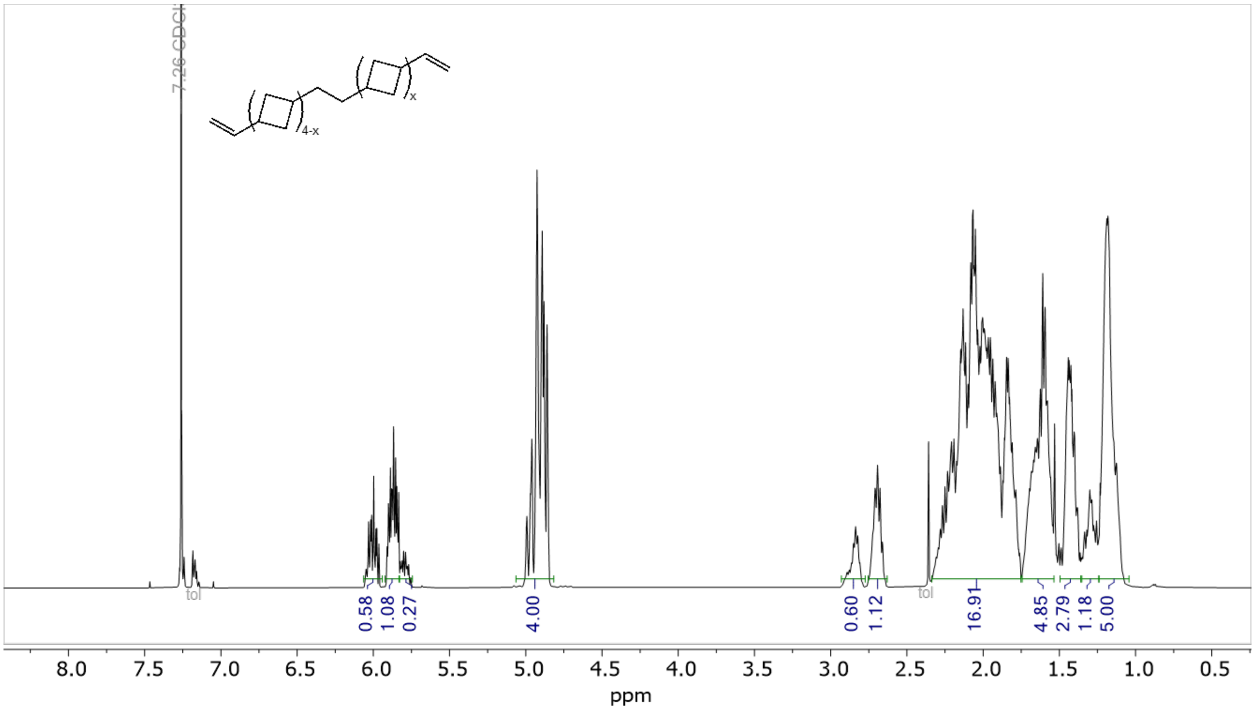


**Figure S11.** Representative $^{1}H$ NMR spectrum for the B4H1 oligomer. $^{1}H$ NMR (500 MHz, chloroform-*d*, 23 ºC) δ 6.06 – 5.95 (m, 0.6H), 5.87 (ddd, *J* = 16.8, 10.4, 6.4, 3.8 Hz, 1H), 5.79 (dtd, *J* = 17.0, 6.4, 3.6 Hz, 0.3H), 5.02 – 4.83 (m,

4H), 2.95 – 2.78 (m, 0.6H), 2.70 (h, $J$ = 8.1 Hz, 1H), 2.34 – 1.75 (m, 17H), 1.75 – 1.54 (m, 5H), 1.51 – 1.36 (m, 3H), 1.35 – 1.25 (m, 1H), 1.25 – 1.05 (m, 5H).

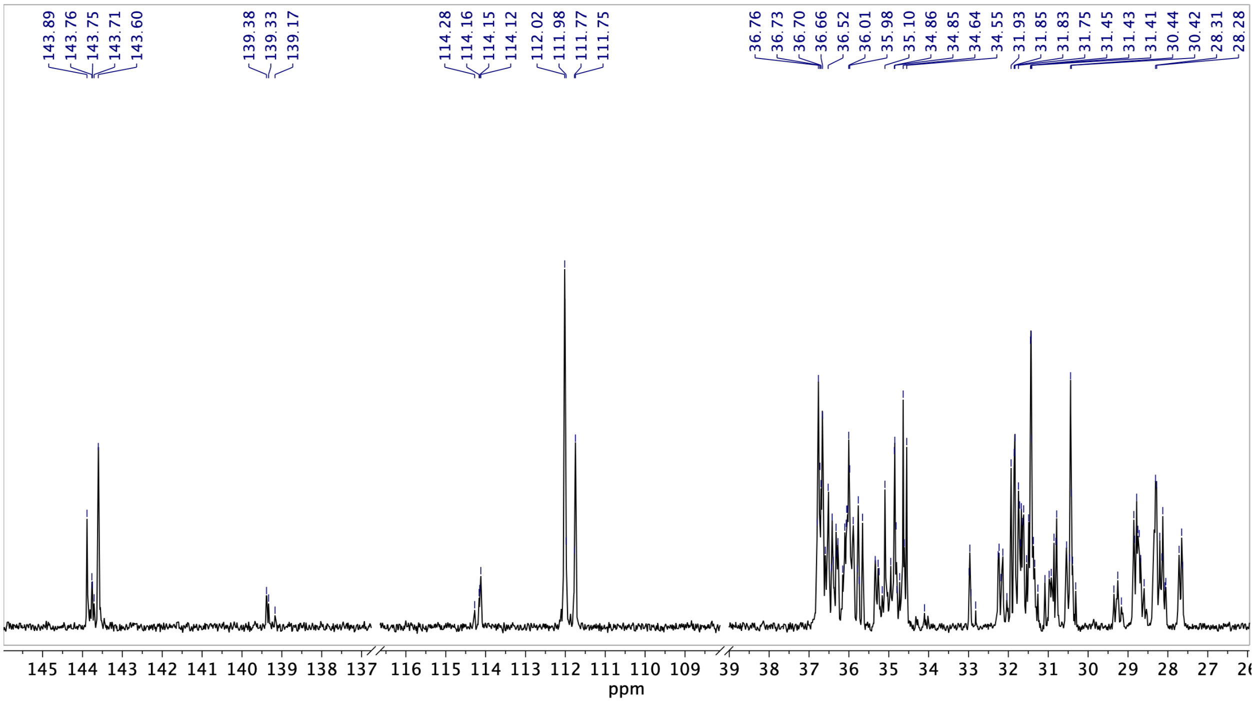

**Figure S12.** Representative $^{13}$C NMR spectrum for the B4H1 oligomer. $^{13}C\{^1H\}$ NMR (126 MHz, chloroform-*d*, 23 ºC) δ 143.89, 143.76, 143.75, 143.71, 143.60, 139.38, 139.33, 139.17, 114.28, 114.16, 114.15, 114.12, 112.02, 111.98, 111.77, 111.75, 36.80, 36.76, 36.73, 36.70, 36.66, 36.59, 36.52, 36.44, 36.42, 36.39, 36.32, 36.30, 36.27, 36.16, 36.10, 36.07, 36.05, 36.03, 36.01, 35.98, 35.89, 35.79, 35.76, 35.74, 35.66, 35.34, 35.27, 35.24, 35.16, 35.10, 34.95, 34.86, 34.85, 34.82, 34.80, 34.73, 34.64, 34.61, 34.55, 34.10, 32.99, 32.97, 32.94, 32.82, 32.26, 32.23, 32.19, 32.14, 32.04, 31.93, 31.85, 31.83, 31.75, 31.72, 31.70, 31.68, 31.64, 31.62, 31.54, 31.49, 31.45, 31.43, 31.41, 31.37, 31.34, 31.26, 31.08, 30.98, 30.92, 30.86, 30.81, 30.79, 30.54, 30.47, 30.44, 30.42, 30.39, 30.31, 29.35, 29.26, 29.16, 28.85, 28.83, 28.78, 28.75, 28.72, 28.68, 28.60, 28.31, 28.28, 28.20, 28.15, 28.13, 28.07, 28.05, 27.72, 27.65, 27.63.

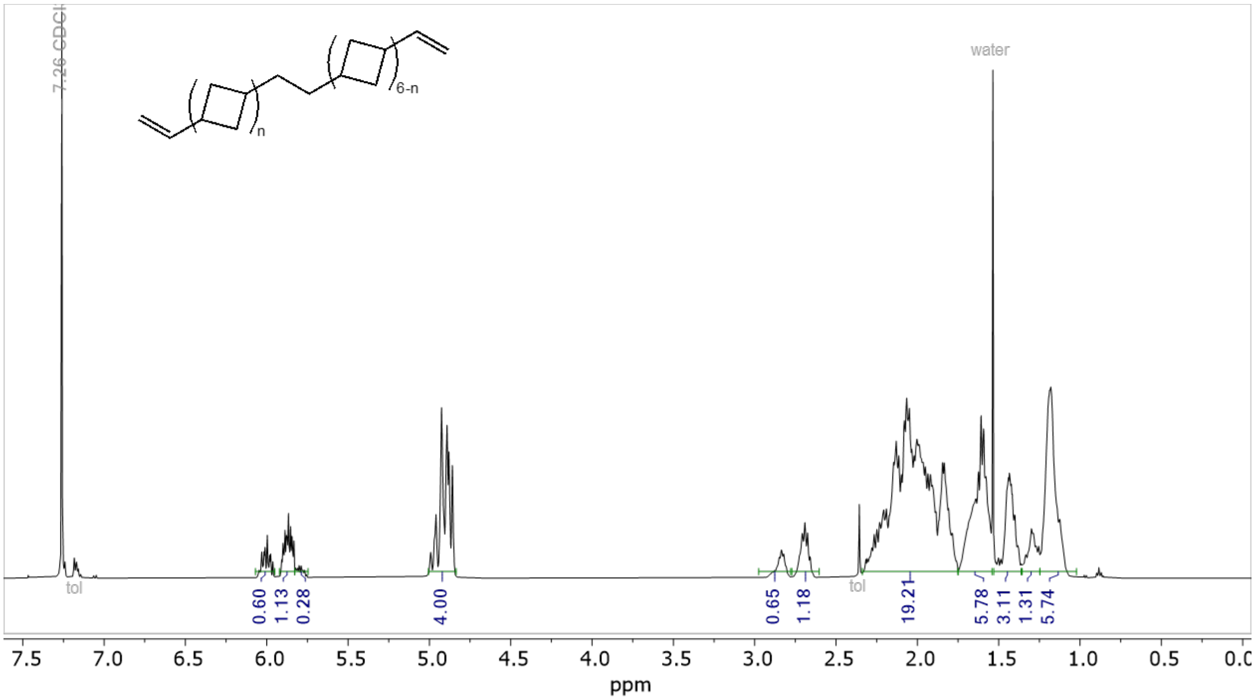


**Figure S13.** Representative $^{1}$H NMR spectrum for the B6H1 oligomer. $^{1}$H NMR (500 MHz, chloroform-*d*, 23 ºC) δ 6.00 (ddd, *J* = 17.0, 10.2, 6.9 Hz, 0.6H), 5.87 (ddd, *J* = 20.8, 10.4, 6.2 Hz, 1H), 5.79 (td, *J* = 10.7, 5.5 Hz, 0.2H), 5.03 – 4.81 (m, 4H), 2.94 – 2.78 (m, 0.7H), 2.68 (h, *J* = 8.2 Hz, 1H), 2.34 – 1.74 (m, 19H), 1.74 – 1.55 (m, 6H), 1.51 – 1.36 (m, 3H), 1.36 – 1.24 (m, 1H), 1.24 – 1.01 (m, 6H).

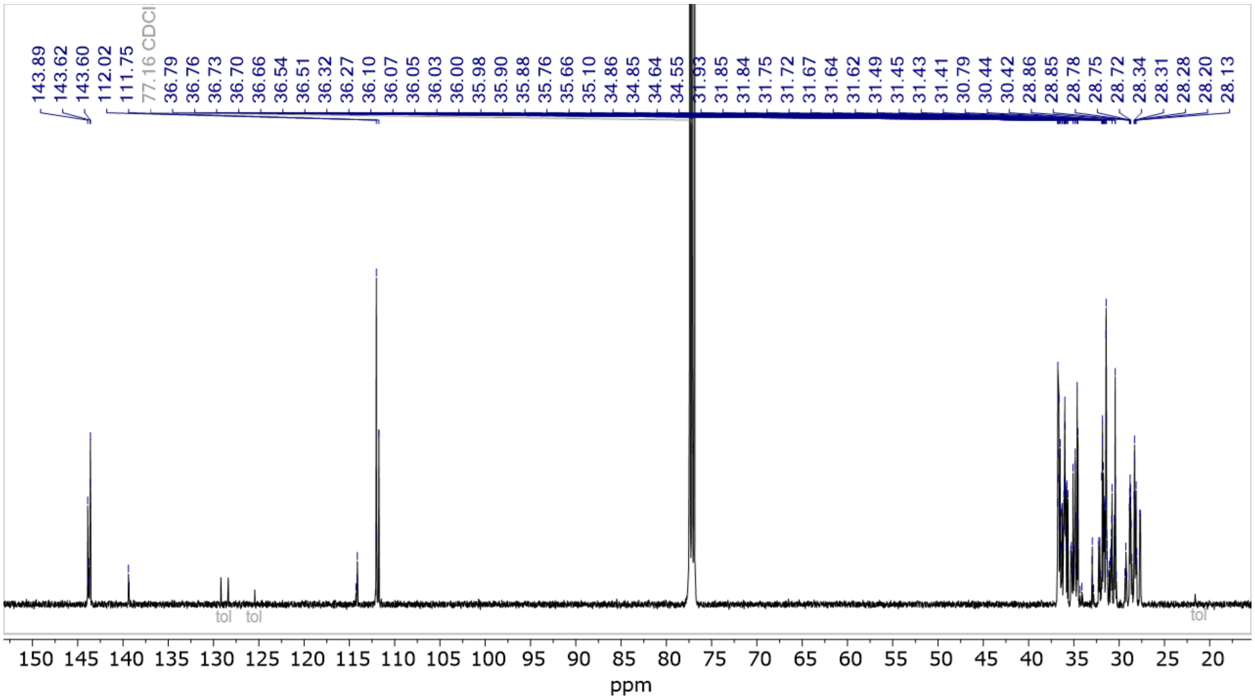


**Figure S14.** Representative $^{13}$C NMR spectrum for the B6H1 oligomer. $^{13}$C{$^{1}$H} NMR (126 MHz, chloroform-*d*, 23 ºC) δ 143.89, 143.82, 143.77, 143.72, 143.62, 143.60, 139.39, 139.34, 114.27, 114.16, 114.12, 112.02, 111.98, 111.75, 36.79, 36.76, 36.73, 36.70, 36.66, 36.59, 36.54, 36.51, 36.44, 36.42, 36.39, 36.32, 36.30, 36.27, 36.15, 36.12, 36.10, 36.07,

36.05, 36.03, 36.00, 35.98, 35.90, 35.88, 35.79, 35.76, 35.66, 35.34, 35.27, 35.24, 35.16, 35.10, 34.95, 34.86, 34.85, 34.81, 34.73, 34.64, 34.61, 34.60, 34.55, 34.31, 34.10, 32.99, 32.97, 32.94, 32.82, 32.26, 32.23, 32.14, 32.04, 31.93, 31.85, 31.84, 31.75, 31.72, 31.70, 31.67, 31.64, 31.62, 31.54, 31.49, 31.45, 31.43, 31.41, 31.38, 31.34, 31.26, 31.08, 30.98, 30.95, 30.92, 30.90, 30.88, 30.86, 30.81, 30.79, 30.54, 30.47, 30.44, 30.42, 30.39, 30.31, 29.35, 29.26, 29.16, 28.86, 28.85, 28.78, 28.75, 28.72, 28.68, 28.60, 28.34, 28.31, 28.28, 28.20, 28.15, 28.13, 28.07, 28.05, 27.72, 27.65.

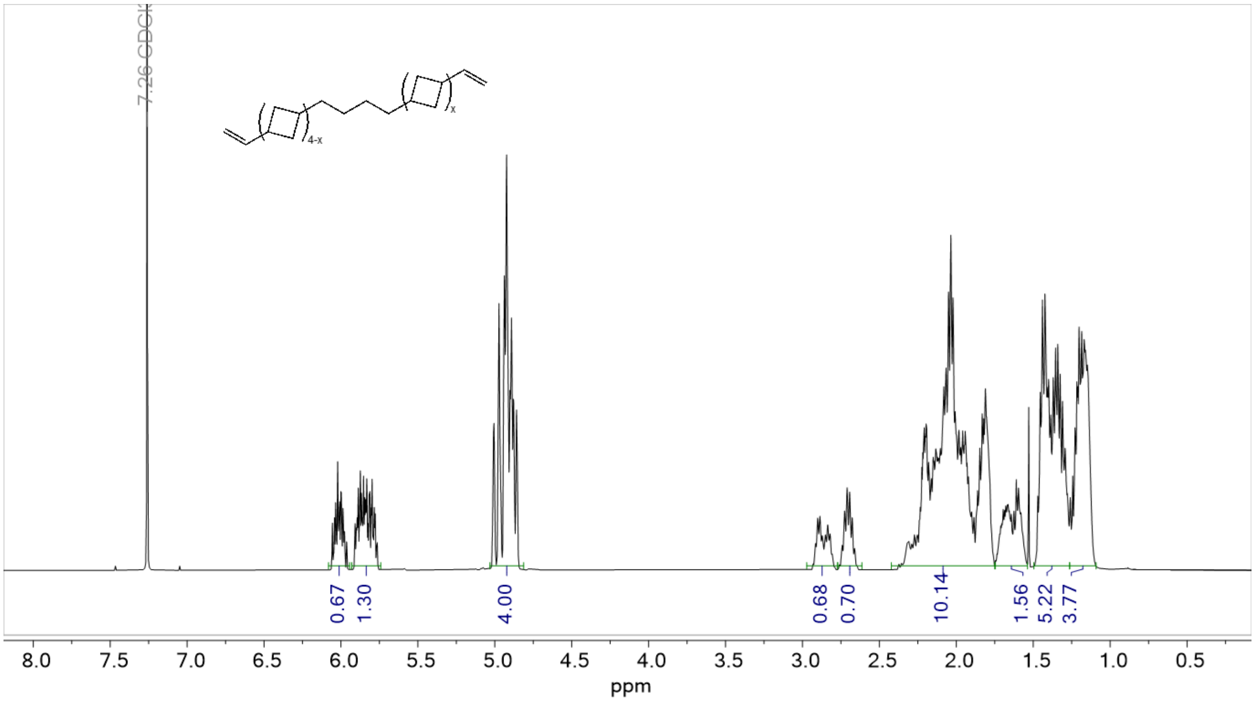


**Figure S15.** Representative $^{1}H$ NMR spectrum for the B4O1 oligomer. $^{1}H$ NMR (500 MHz, Chloroform-*d*) δ 6.07 – 5.95 (m, 0.6 H), 5.92 – 5.75 (m, 1H), 5.03 – 4.82 (m, 4H), 2.96 – 2.77 (m, 1H), 2.77 – 2.63 (m, *J* = 8.5 Hz, 1H), 2.39 – 1.75 (m, 16H), 1.75 – 1.54 (m, 4H), 1.50 – 1.27 (m, 6H), 1.27 – 1.08 (m, 6H).

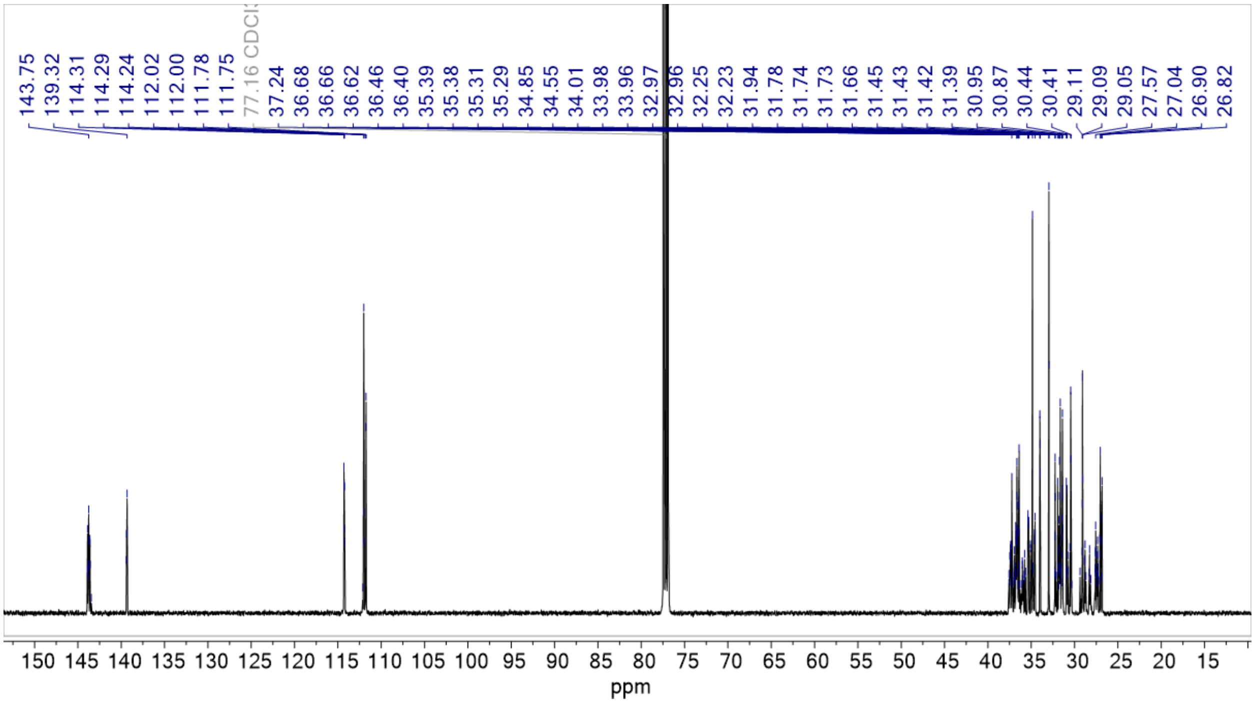


**Figure S16.** Representative $^{13}C$ NMR spectrum for the B4O1 oligomer. $^{13}C$ NMR (126 MHz, $CDCl_3$) δ 143.89, 143.87, 143.82, 143.80, 143.77, 143.75, 143.72, 143.61, 143.60, 143.58, 143.45, 139.40, 139.38, 139.32, 114.31, 114.29, 114.26, 114.24, 114.23, 112.12, 112.02, 112.00, 111.98, 111.78, 111.75, 37.56, 37.51, 37.46, 37.42, 37.37, 37.34, 37.32, 37.24, 37.19, 37.16, 36.99, 36.96, 36.92, 36.85, 36.83, 36.79, 36.77, 36.73, 36.69, 36.68, 36.66, 36.64, 36.62, 36.60, 36.59, 36.55, 36.52, 36.46, 36.44, 36.42, 36.40, 36.31, 36.06, 36.03, 36.01, 35.99, 35.92, 35.77, 35.74, 35.66, 35.39, 35.38, 35.31, 35.29, 35.10, 35.08, 34.85, 34.81, 34.79, 34.64, 34.61, 34.55, 34.01, 33.98, 33.96, 32.97, 32.96, 32.29, 32.27, 32.25, 32.23, 32.16, 32.12, 31.94, 31.93, 31.86, 31.83, 31.81, 31.78, 31.76, 31.74, 31.73, 31.69, 31.68, 31.66, 31.54, 31.52, 31.51, 31.49, 31.48, 31.45, 31.43, 31.42, 31.39, 31.36, 30.95, 30.91, 30.88, 30.87, 30.81, 30.79, 30.53, 30.44, 30.41, 30.34, 29.37, 29.12, 29.11, 29.09, 29.05, 28.86, 28.82, 28.80, 28.68, 28.30, 28.28, 28.16, 28.14, 27.62, 27.59, 27.58, 27.57, 27.54, 27.51, 27.42, 27.40, 27.38, 27.35, 27.34, 27.31, 27.28, 27.09, 27.08, 27.06, 27.04, 26.92, 26.90, 26.88, 26.82.

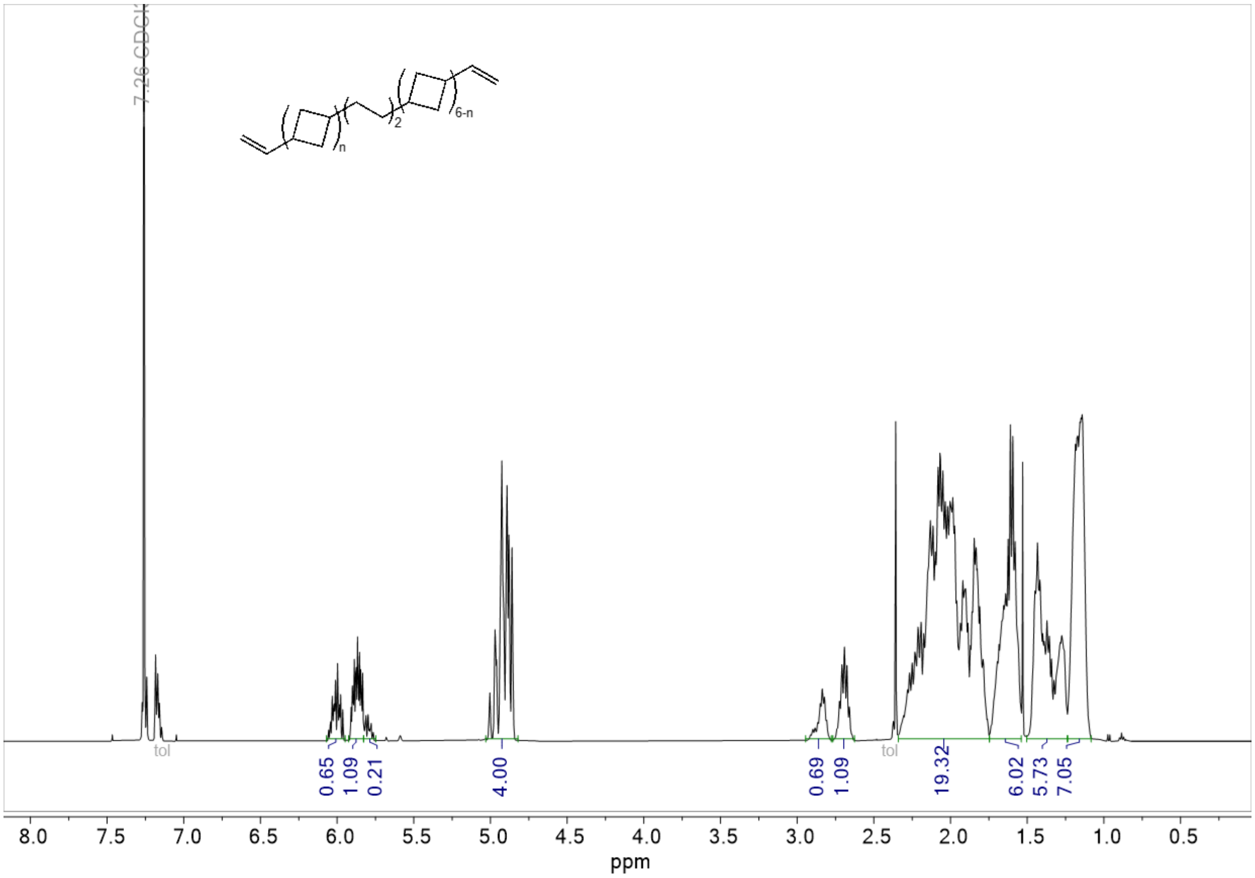


**Figure S17.** Representative $^1$H NMR spectrum for the B6O1 oligomer. $^1$H NMR (500 MHz, chloroform-*d*, 23 ºC) δ 6.00 (ddd, *J* = 17.1, 10.2, 6.4 Hz, 0.7H), 5.92 – 5.83 (m, 1H), 5.83 – 5.76 (m, 0.2H), 5.03 – 4.83 (m, 4H), 2.94 – 2.78 (m, 0.7H), 2.69 (hept, *J* = 8.5 Hz, 1H), 2.34 – 1.75 (m, 19H), 1.75 – 1.54 (m, 6H), 1.50 – 1.24 (m, 6H), 1.24 – 1.07 (m, 7H).

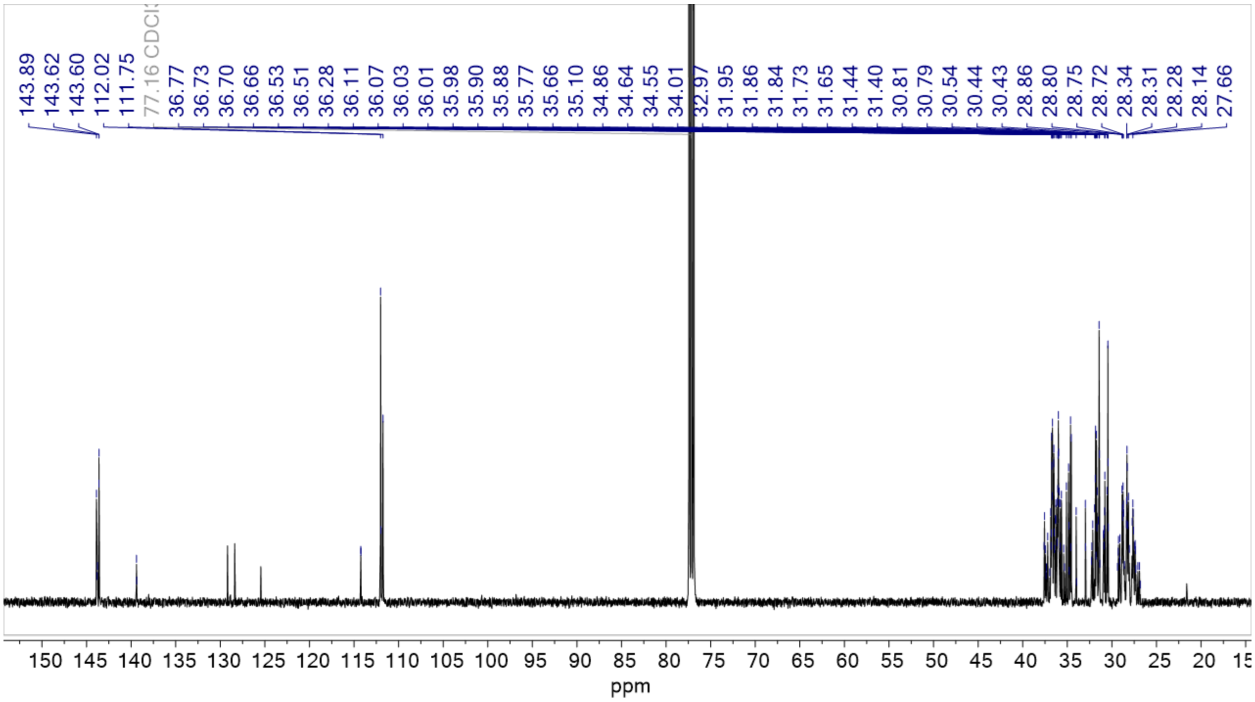


**Figure S18.** Representative $^{13}$C NMR spectrum for the B6O1 oligomer. $^{13}$C NMR (126 MHz, $CDCl_3$) δ 143.89, 143.82, 143.77, 143.62, 143.60, 139.40, 139.38, 114.24, 114.22, 112.02, 111.98, 111.75, 37.60, 37.56, 37.46, 37.38, 37.34, 37.19, 36.96, 36.85, 36.81, 36.79, 36.77, 36.73, 36.70, 36.66, 36.53, 36.51, 36.46, 36.43, 36.32, 36.30, 36.28, 36.16, 36.11, 36.07, 36.03, 36.01, 35.98, 35.90, 35.88, 35.79, 35.77, 35.66, 35.40, 35.31, 35.10, 34.86, 34.81, 34.79, 34.64, 34.61, 34.55, 34.01, 33.96, 32.97, 32.96, 32.28, 32.24, 32.15, 31.95, 31.86, 31.84, 31.78, 31.73, 31.69, 31.65, 31.54, 31.44, 31.40, 31.36, 30.95, 30.91, 30.89, 30.87, 30.81, 30.79, 30.54, 30.44, 30.43, 30.41, 29.37, 29.28, 29.26, 29.12, 29.09, 28.86, 28.80, 28.78, 28.75, 28.72, 28.69, 28.60, 28.37, 28.34, 28.31, 28.28, 28.21, 28.14, 28.05, 27.72, 27.66, 27.62, 27.57, 27.44, 27.39, 27.36, 27.31, 27.08, 26.90, 26.82.

## S4.2. Polymer NMR Data

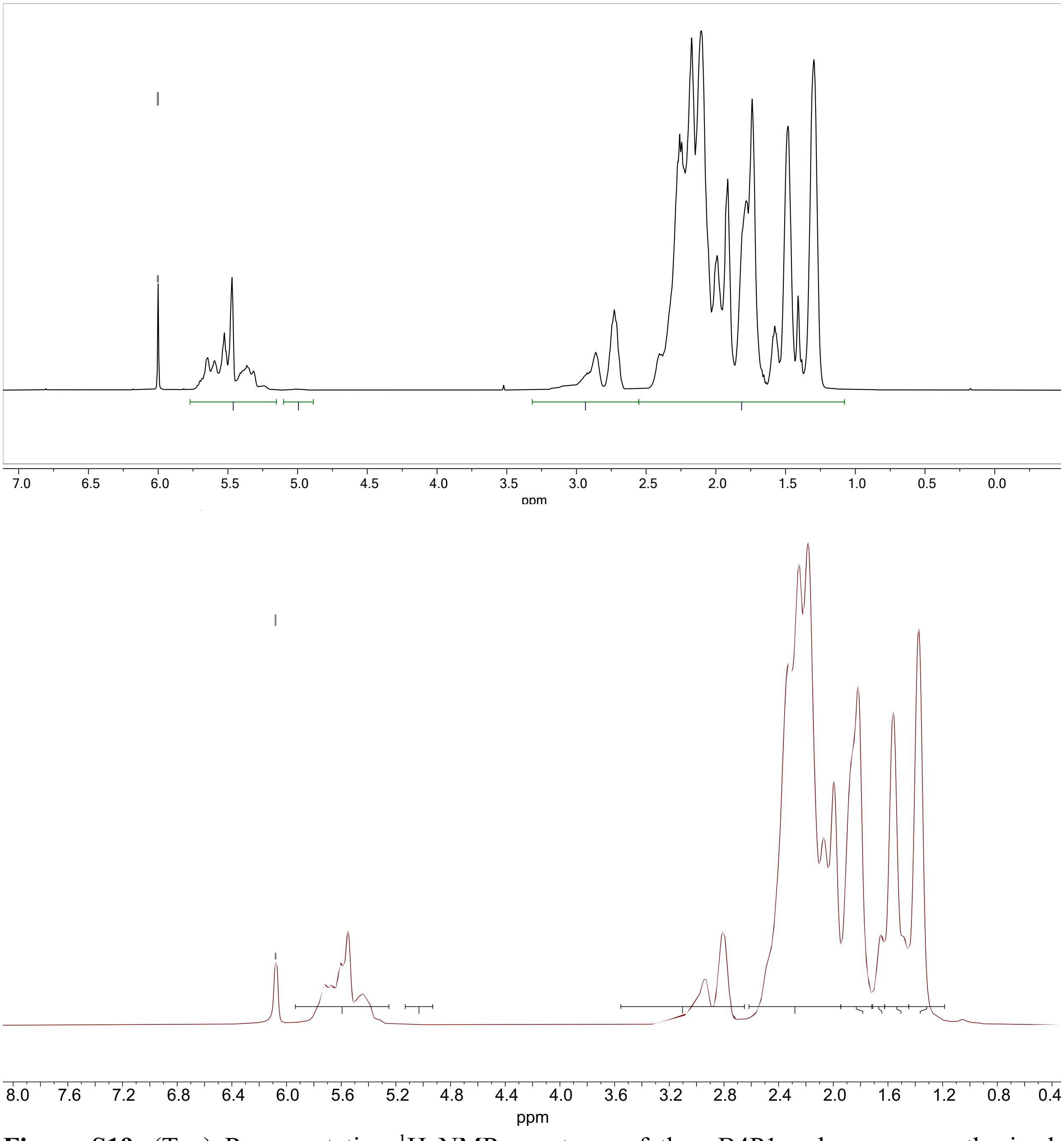


**Figure S19.** (Top) Representative $^{1}H$ NMR spectrum of the pB4P1 polymer as synthesized. (Bottom) Representative $^{1}H$ NMR spectrum of the pB4P1 polymer after melt-pressing.

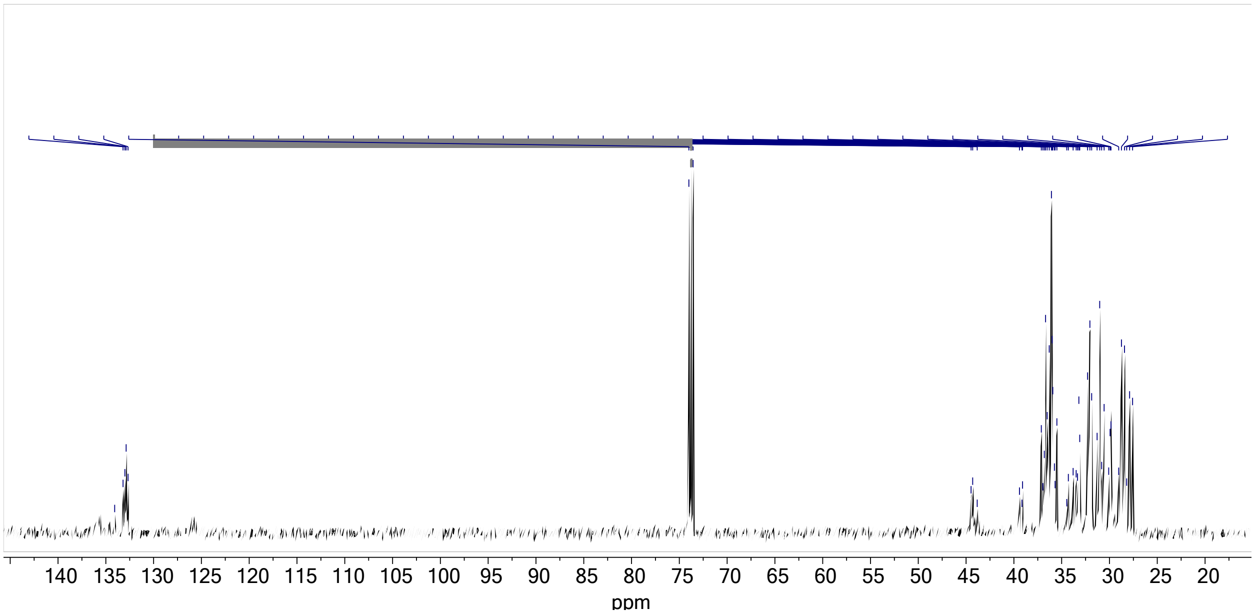


**Figure S20.** Representative $^{13}$C NMR spectrum of the pB4P1 polymer. $^{13}$C NMR (126 MHz, Tetrachloroethane-$d_2$) δ 134.07, 133.20, 133.00, 132.87, 132.67, 74.00, 73.78, 73.56, 44.48, 44.31, 43.83, 39.42, 39.15, 39.09, 37.13, 36.96, 36.82, 36.69, 36.51, 36.30, 36.07, 36.00, 35.93, 35.76, 35.69, 35.50, 34.47, 34.30, 33.80, 33.48, 33.34, 33.20, 33.11, 32.29, 32.05, 31.86, 31.29, 31.02, 30.82, 30.56, 30.08, 29.93, 29.84, 29.03, 28.74, 28.43, 28.20, 27.89, 27.58.

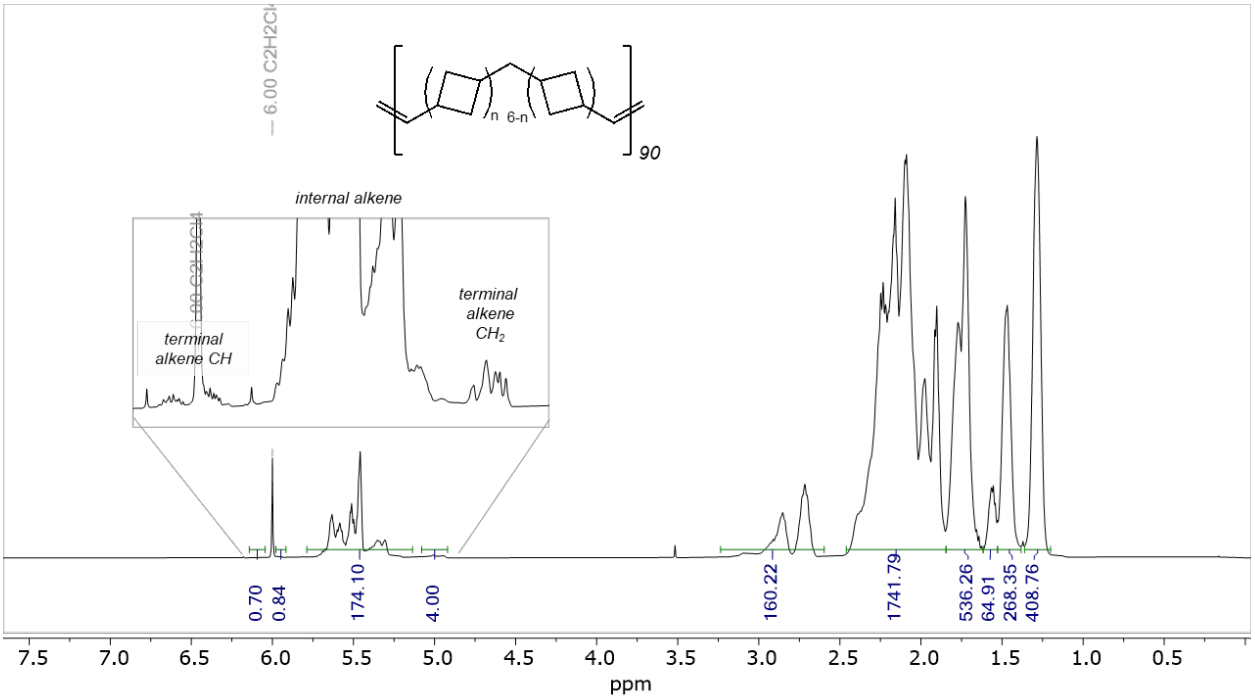


**Figure S21.** Representative $^{1}$H NMR spectrum of the pB6P1 polymer.

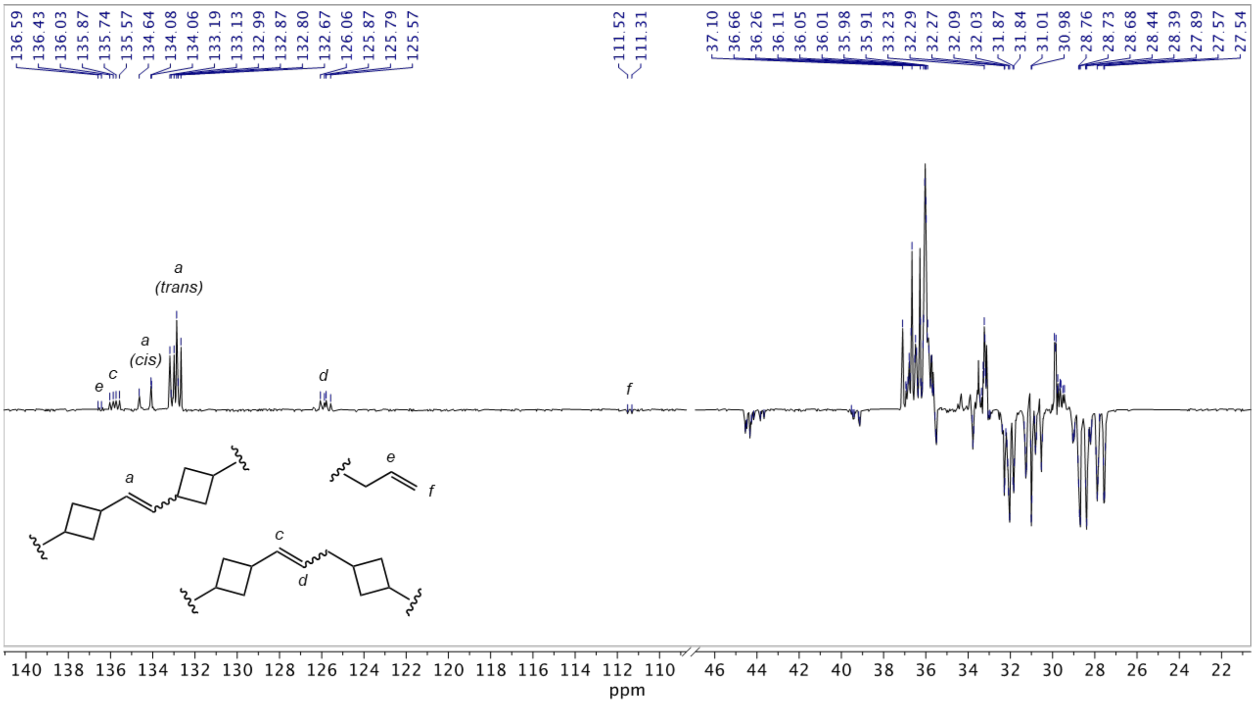


**Figure S22.** Representative $^{13}$C NMR spectrum of the pB6P1 polymer. $^{13}$C NMR (126 MHz, Tetrachloroethane-$d_2$) δ 136.59, 136.43, 136.03, 135.87, 135.74, 135.57, 134.64, 134.08, 134.06, 133.19, 133.13, 132.99, 132.87, 132.80, 132.67, 126.06, 125.87, 125.79, 125.57, 111.52, 111.31, 44.55, 44.48, 44.33, 44.26, 44.15, 43.84, 43.66, 39.52, 39.45, 39.40, 39.18, 39.13, 37.10, 36.94, 36.91, 36.84, 36.79, 36.78, 36.75, 36.70, 36.66, 36.52, 36.49, 36.44, 36.40, 36.37, 36.26, 36.24, 36.21, 36.17, 36.13, 36.11, 36.05, 36.01, 35.98, 35.94, 35.91, 35.86, 35.78, 35.73, 35.69, 35.67, 35.64, 35.57, 35.53, 35.50, 33.81, 33.77, 33.74, 33.43, 33.36, 33.28, 33.26, 33.23, 33.20, 33.18, 33.14, 33.05, 32.96, 32.38, 32.29, 32.27, 32.21, 32.17, 32.12, 32.09, 32.03, 31.87, 31.84, 31.81, 31.34, 31.31, 31.29, 31.27, 31.24, 31.04, 31.01, 30.98, 30.86, 30.81, 30.76, 30.53, 30.48, 29.91, 29.87, 29.84, 29.77, 29.75, 29.66, 29.64, 29.61, 29.52, 29.44, 29.04, 29.02, 28.97, 28.94, 28.79, 28.76, 28.73, 28.68, 28.44, 28.39, 28.29, 28.23, 28.20, 28.17, 28.15, 27.95, 27.92, 27.89, 27.86, 27.83, 27.78, 27.60, 27.57, 27.54, 27.51.

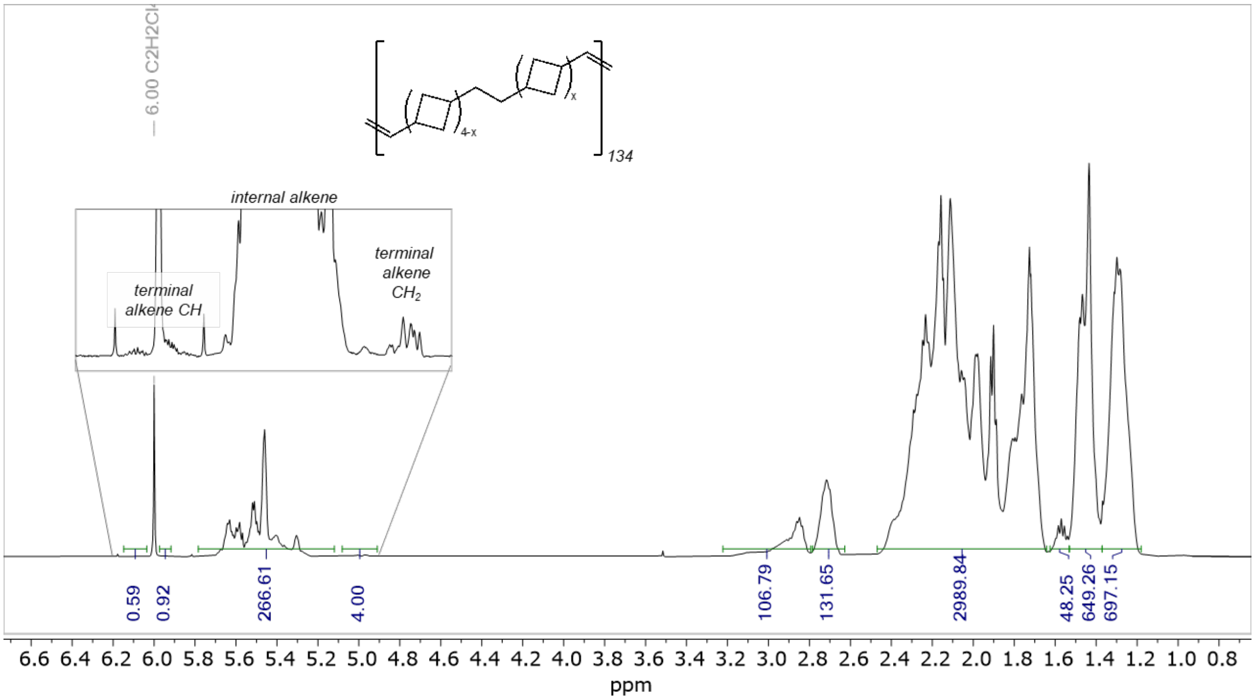


**Figure S23.** Representative $^{1}H$ NMR spectrum of the pB4H1 polymer.

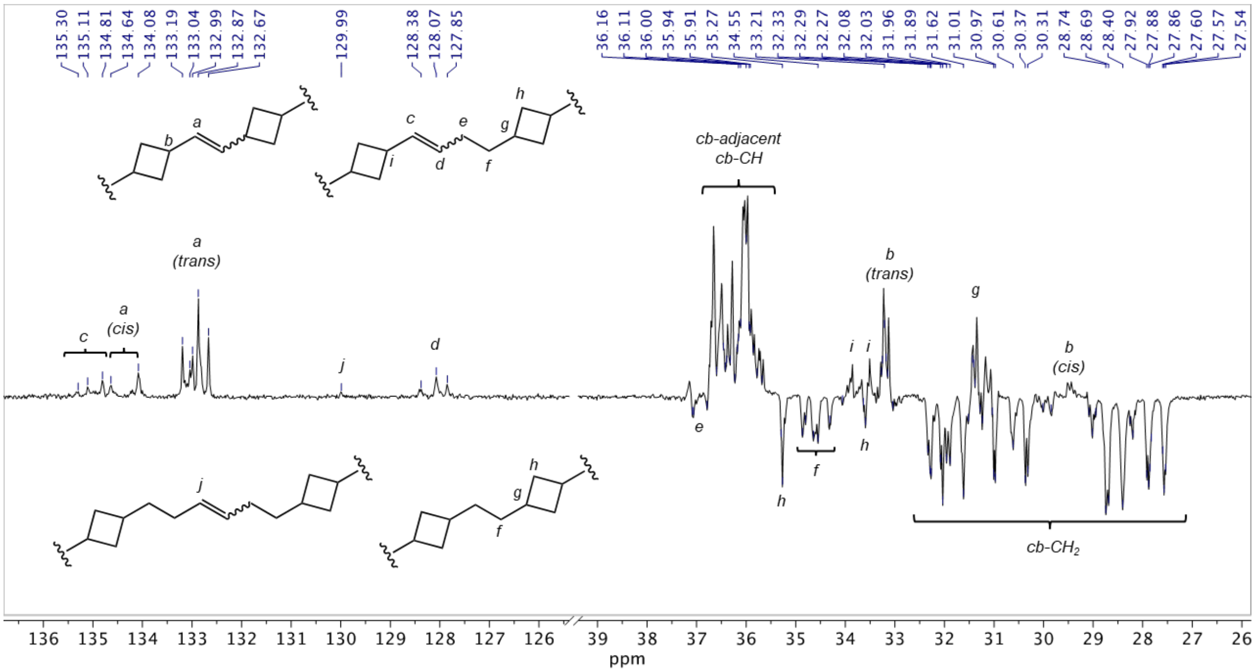


**Figure S24.** Representative $^{13}C$ NMR spectrum of the pB4H1 polymer. $^{13}C$ NMR (126 MHz, Tetrachloroethane-$d_2$) δ 135.30, 135.11, 134.81, 134.64, 134.08, 133.19, 133.04, 132.99, 132.87, 132.67, 129.99, 128.38, 128.07, 127.85, 37.09, 37.06, 36.79, 36.60, 36.46, 36.42, 36.40, 36.32, 36.23, 36.18, 36.16, 36.11, 36.00, 35.94, 35.91, 35.86, 35.78, 35.68, 35.30, 35.27, 34.87, 34.80, 34.64, 34.55, 34.31, 34.06, 33.63, 33.59, 33.27, 33.25, 33.21, 33.18, 33.15, 33.04, 32.33, 32.29, 32.27, 32.08, 32.03, 31.96, 31.89, 31.62, 31.52, 31.43, 31.41, 31.38, 31.28, 31.24, 31.04, 31.01, 30.97, 30.61, 30.37, 30.31, 30.01, 29.85, 29.08, 29.02, 28.95, 28.74, 28.69, 28.40, 28.26, 28.20, 27.92, 27.88, 27.86, 27.83, 27.60, 27.57, 27.54.

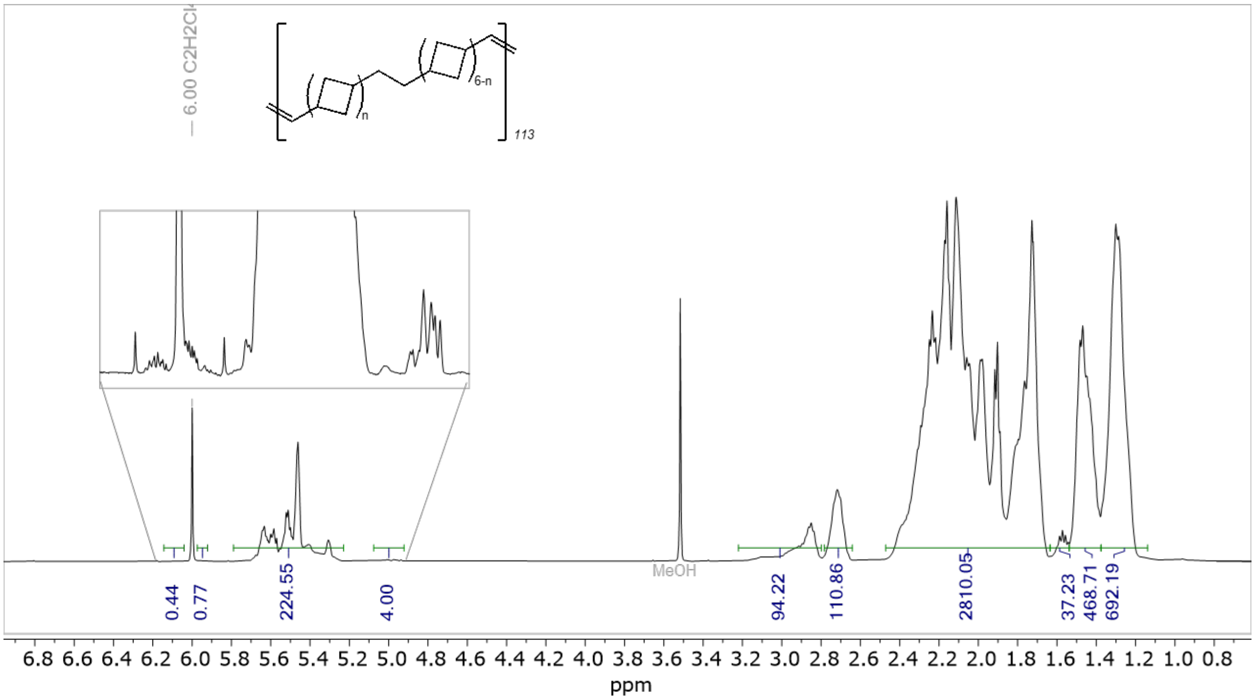

**Figure S25.** Representative $^1$H NMR spectrum of the pB6H1 polymer.

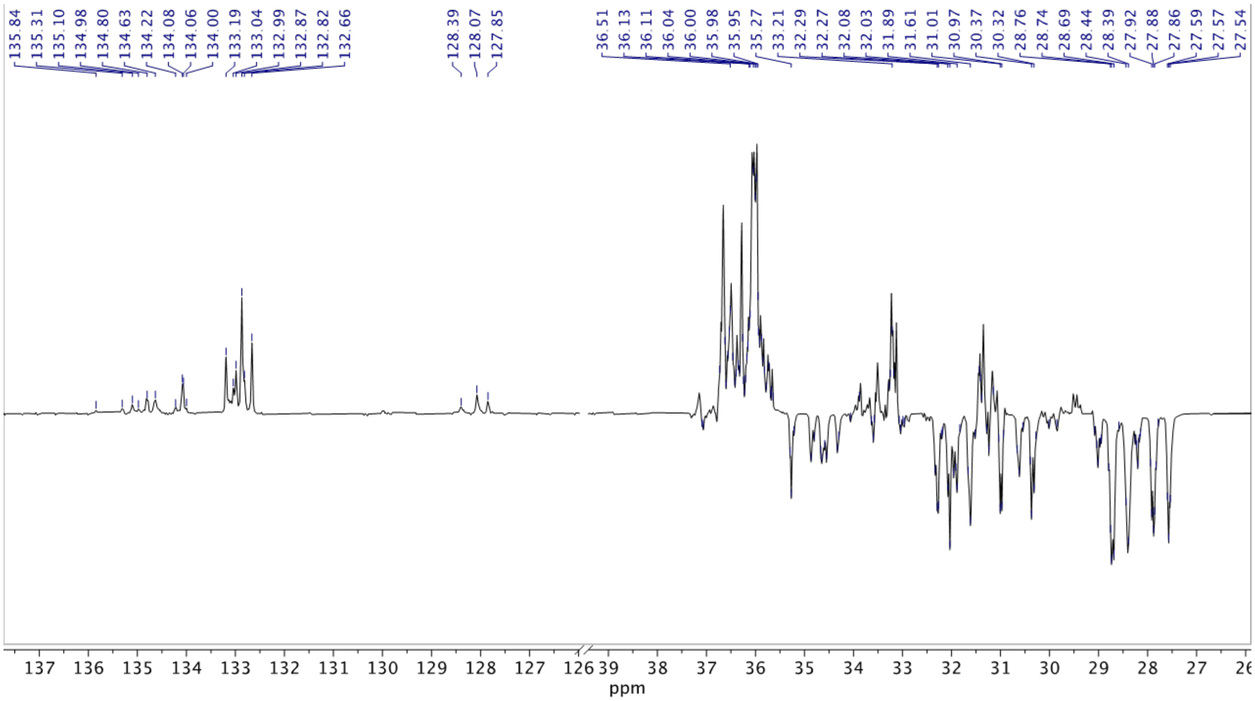

**Figure S26.** Representative $^{13}$C NMR spectrum of the pB6H1 polymer. $^{13}$C NMR (126 MHz, Tetrachloroethane-$d_2$) δ 135.84, 135.31, 135.10, 134.98, 134.80, 134.63, 134.22, 134.08, 134.06, 134.00, 133.19, 133.04, 132.99, 132.87, 132.82, 132.66, 128.39, 128.07, 127.85, 37.09, 37.06, 36.73, 36.62, 36.60, 36.56, 36.53, 36.51, 36.46, 36.42, 36.39, 36.35, 36.32, 36.26, 36.24, 36.23, 36.20, 36.18, 36.16, 36.13, 36.11, 36.04, 36.00, 35.98, 35.95, 35.94, 35.91, 35.87, 35.85, 35.81, 35.78, 35.73, 35.69, 35.67, 35.64, 35.30, 35.27, 35.21, 34.87, 34.80, 34.67, 34.65, 34.59, 34.55, 34.33, 34.30, 34.06, 33.88, 33.63, 33.59, 33.57, 33.27, 33.25, 33.21, 33.18, 33.07, 33.04, 32.99, 32.96, 32.33, 32.29, 32.27, 32.20, 32.19, 32.08, 32.03, 31.96, 31.91, 31.89,

31.83, 31.66, 31.61, 31.52, 31.43, 31.41, 31.39, 31.28, 31.24, 31.14, 31.04, 31.01, 30.97, 30.94, 30.66, 30.62, 30.55, 30.39, 30.37, 30.32, 30.28, 30.01, 29.85, 29.08, 29.04, 29.01, 28.97, 28.95, 28.79, 28.76, 28.74, 28.69, 28.58, 28.44, 28.39, 28.26, 28.22, 28.20, 28.17, 28.15, 27.94, 27.92, 27.88, 27.86, 27.83, 27.78, 27.59, 27.57, 27.54.

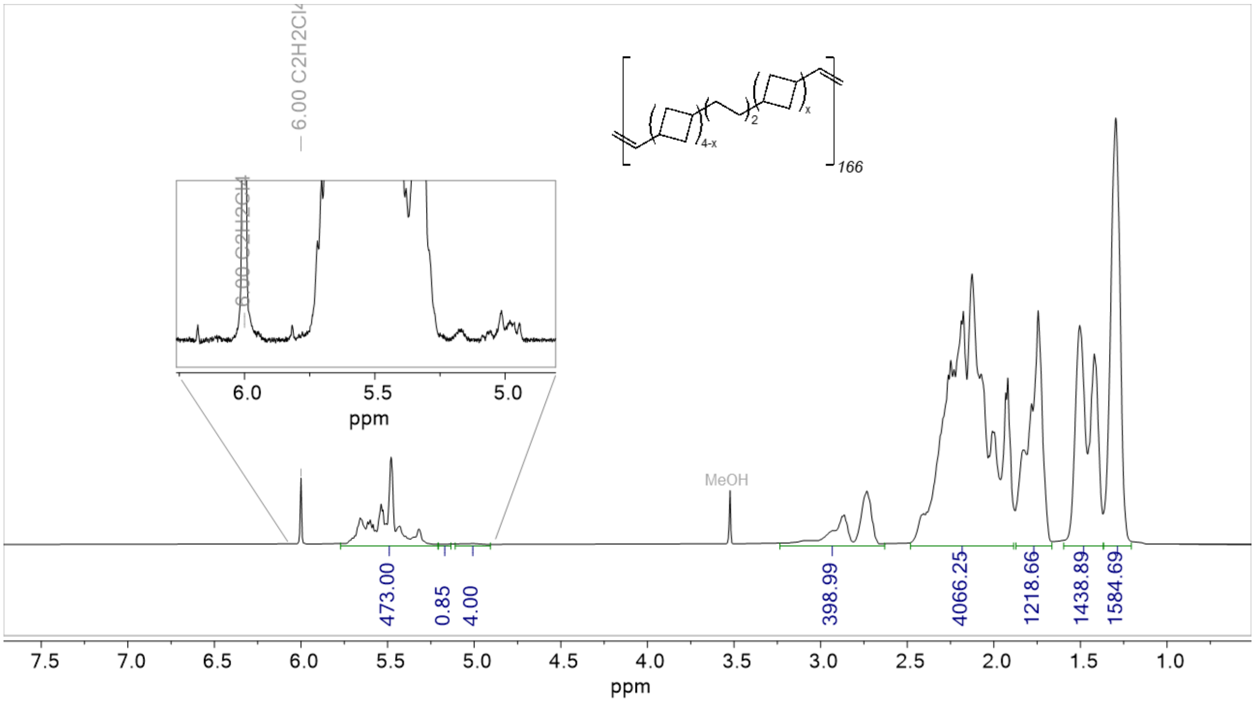


**Figure S27.** Representative $^{1}$H NMR spectrum of the pB4O1 polymer.

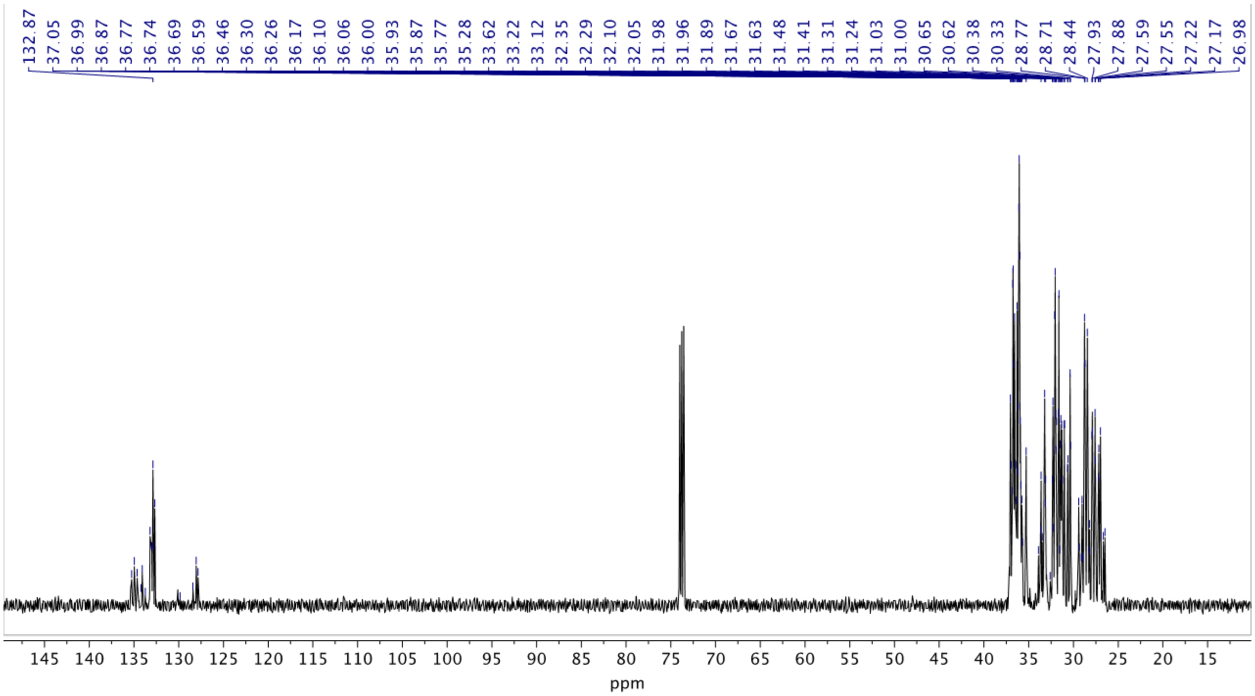


**Figure S28.** Representative $^{13}$C NMR spectrum of the pB4O1 polymer. $^{13}$C NMR (126 MHz, $EtD_2Cl_4$) δ 135.26, 134.96, 134.64, 134.22, 134.08, 133.73, 133.20, 133.00, 132.87, 132.82, 132.67, 129.84, 128.41, 128.04, 127.83, 37.05, 36.99, 36.87, 36.77, 36.74, 36.69, 36.59, 36.46, 36.39, 36.30, 36.26, 36.17, 36.10, 36.06, 36.00, 35.93, 35.87, 35.77, 35.70, 35.28, 33.91, 33.65, 33.62, 33.49, 33.22, 33.15, 33.12, 32.60, 32.35, 32.29, 32.23, 32.10, 32.05, 31.98, 31.96, 31.89, 31.67, 31.63, 31.54, 31.48, 31.41, 31.31, 31.24, 31.03, 31.00, 30.65, 30.62, 30.38, 30.33, 29.40, 29.36, 29.10, 29.03, 28.97, 28.77, 28.71, 28.44, 28.26, 28.20, 27.93, 27.88, 27.59, 27.55, 27.22, 27.17, 26.98, 26.66, 26.48.

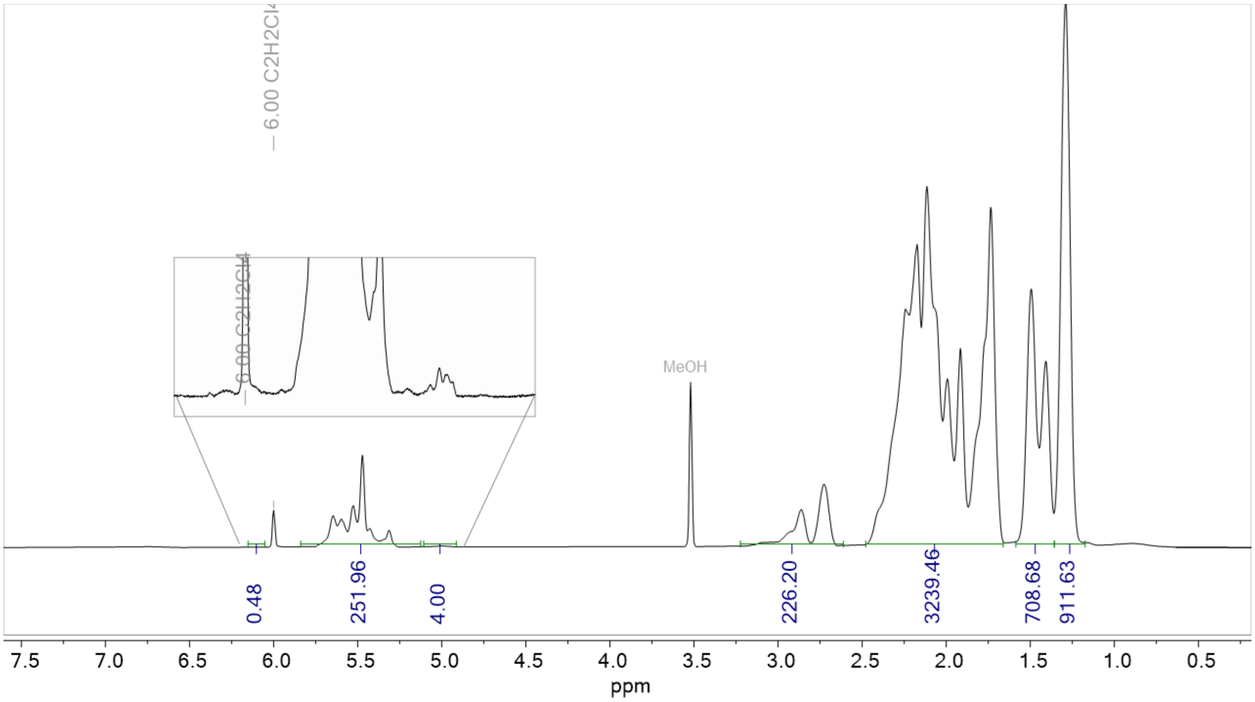


**Figure S29.** Representative $^{1}$H NMR spectrum of the pB6O1 polymer.

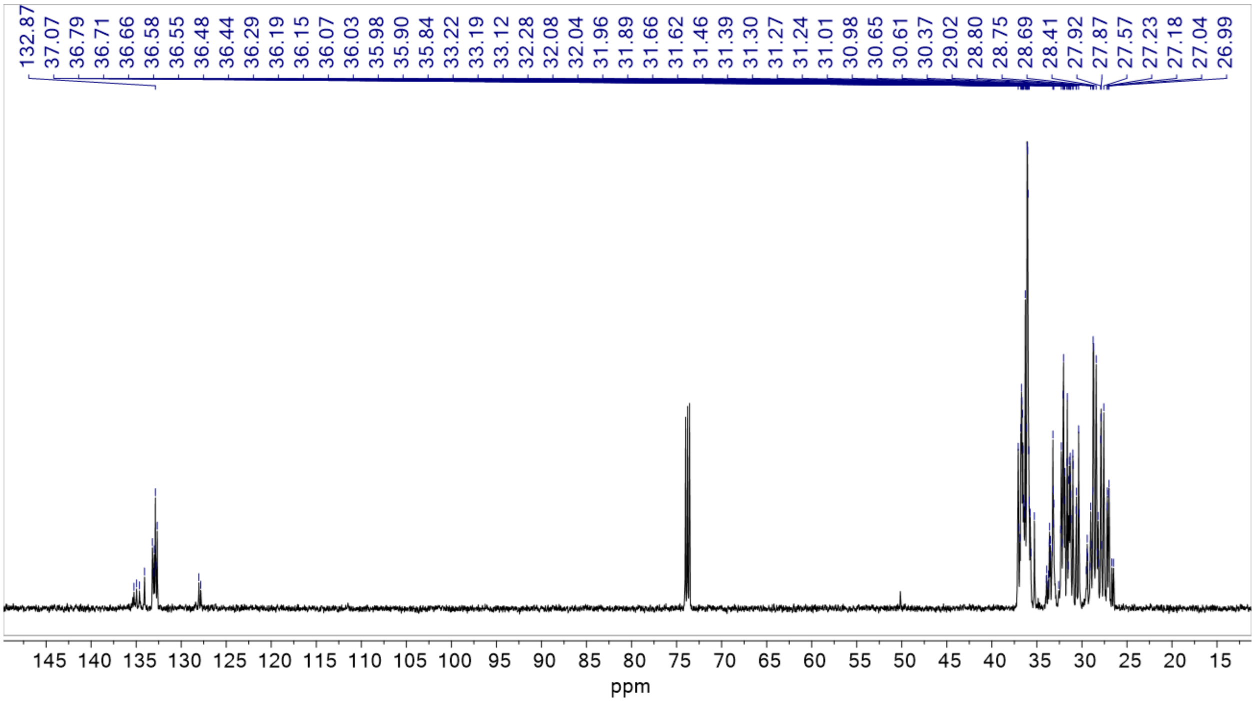

**Figure S30.** Representative $^{13}C$ NMR spectrum of the pB6O1 polymer. $^{13}C$ NMR (126 MHz, $EtD_2Cl_4$) δ 135.25, 134.96, 134.64, 134.07, 133.19, 133.04, 132.99, 132.87, 132.80, 132.66, 128.03, 127.82, 37.07, 37.01, 36.88, 36.79, 36.71, 36.66, 36.58, 36.55, 36.48, 36.44, 36.37, 36.29, 36.19, 36.15, 36.07, 36.03, 35.98, 35.90, 35.84, 35.74, 35.67, 35.28, 33.96, 33.91, 33.76, 33.71, 33.62, 33.59, 33.54, 33.50, 33.29, 33.22, 33.19, 33.16, 33.12, 32.58, 32.35, 32.28, 32.21, 32.08, 32.04, 31.96, 31.89, 31.66, 31.62, 31.52, 31.46, 31.39, 31.30, 31.27, 31.24, 31.20, 31.01, 30.98, 30.65, 30.61, 30.37, 30.31, 29.52, 29.45, 29.41, 29.36, 29.10, 29.02, 28.95, 28.80, 28.75, 28.69, 28.41, 28.26, 28.20, 28.14, 27.92, 27.87, 27.81, 27.57, 27.23, 27.18, 27.04, 26.99, 26.67, 26.49.

## S5. Thermogravimetric Characterization

### S5.1. Oligomer TGA

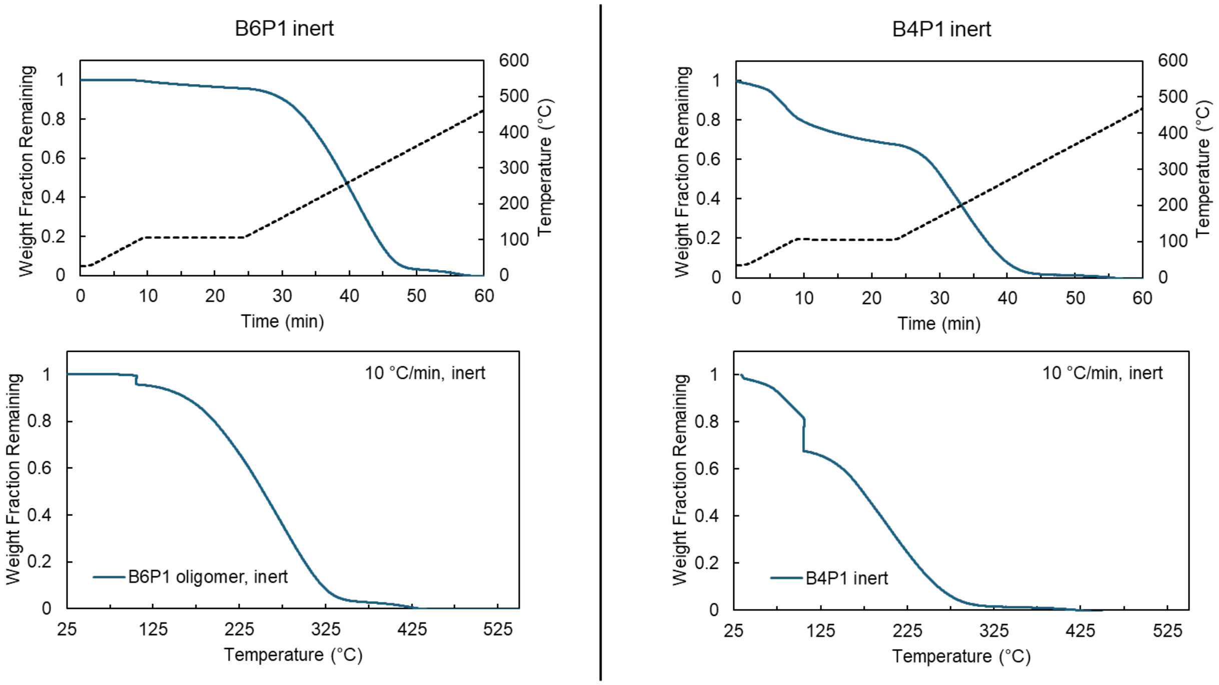


**Figure S31.** TGA measurements for B6P1 (left) and B4P1 (right) oligomers under an inert (nitrogen) gas flow, taken at a ramp rate of 10 °C/min including an isothermal step at 105 °C for 15 min.

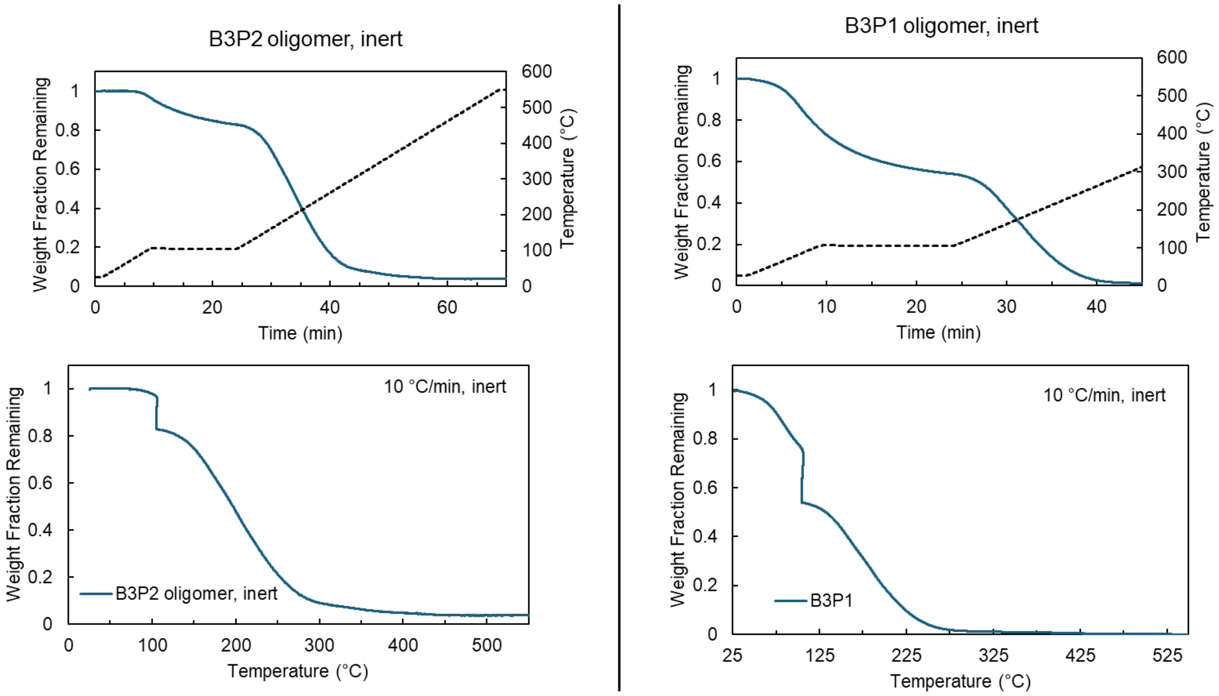


**Figure S32.** TGA measurements for B3P2 (left) and B3P1 (right) oligomers under an inert

(nitrogen) gas flow, taken at a ramp rate of 10 °C/min including an isothermal step at 105 °C for 15 min.

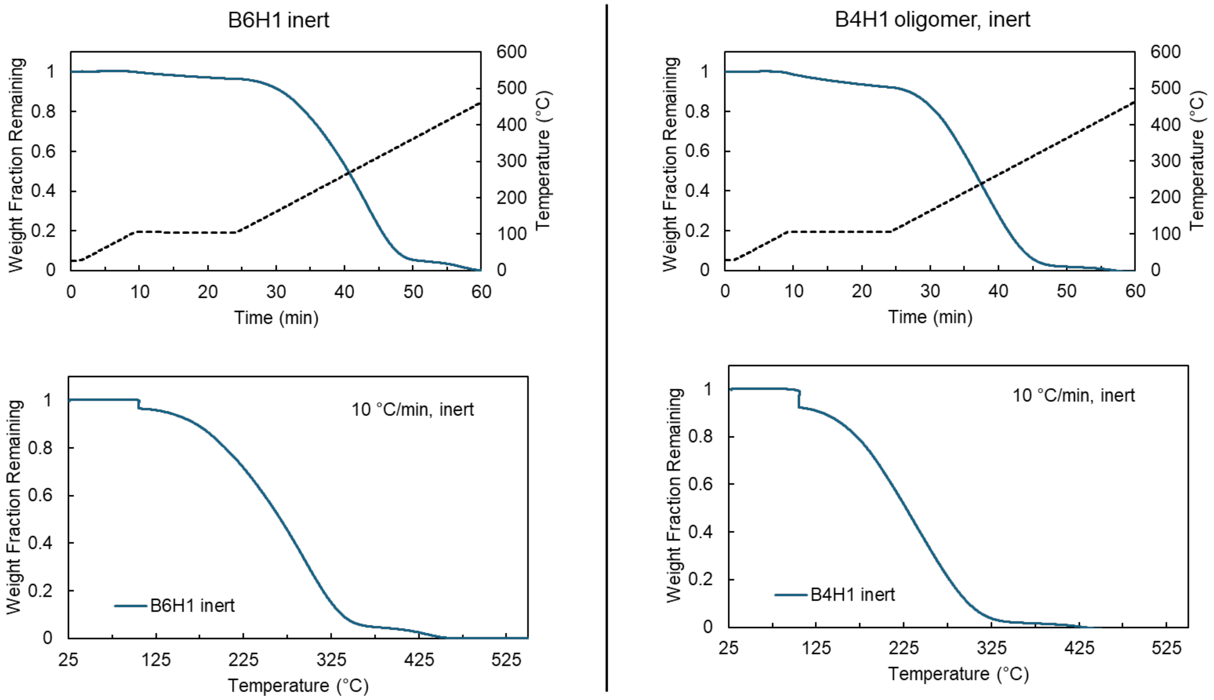


**Figure S33.** TGA measurements for B6H1 (left) and B4H1 (right) oligomers under an inert (nitrogen) gas flow, taken at a ramp rate of 10 °C/min including an isothermal step at 105 °C for 15 min.

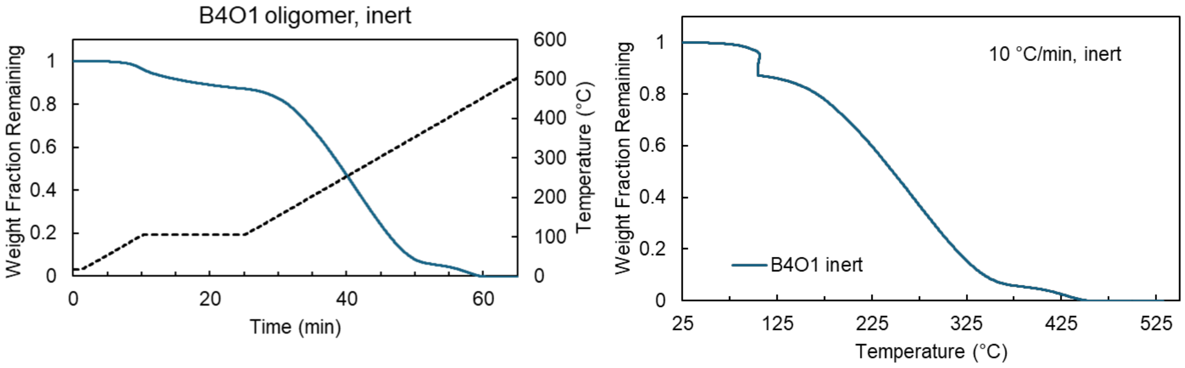


**Figure S34.** TGA measurements for B4O1 oligomer under an inert (nitrogen) gas flow, taken at a ramp rate of 10 °C/min including an isothermal step at 105 °C for 15 min.

## S5.2. Polymer TGA Data

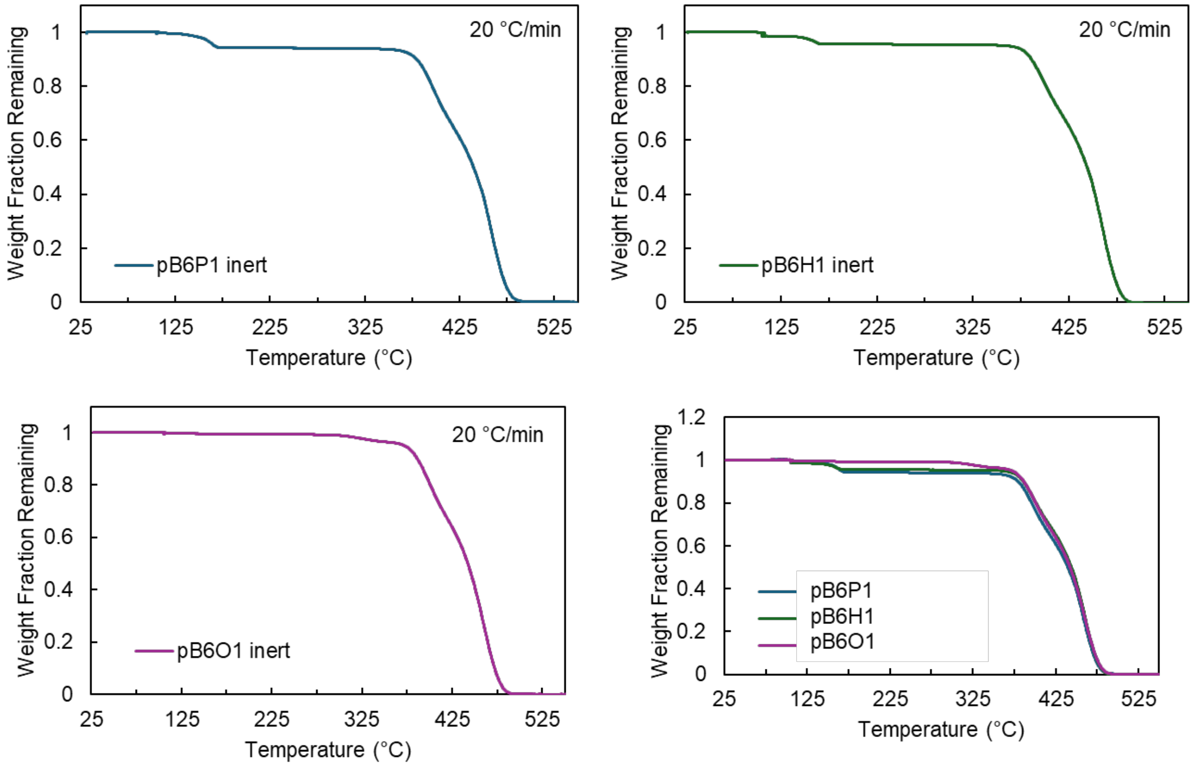


**Figure S35.** TGA measurements for pB6P1, pB6H1, and pB6O1 polymers under an inert (nitrogen) gas flow, taken at a ramp rate of 20 °C/min including an isothermal step at 105 °C for 15 min.

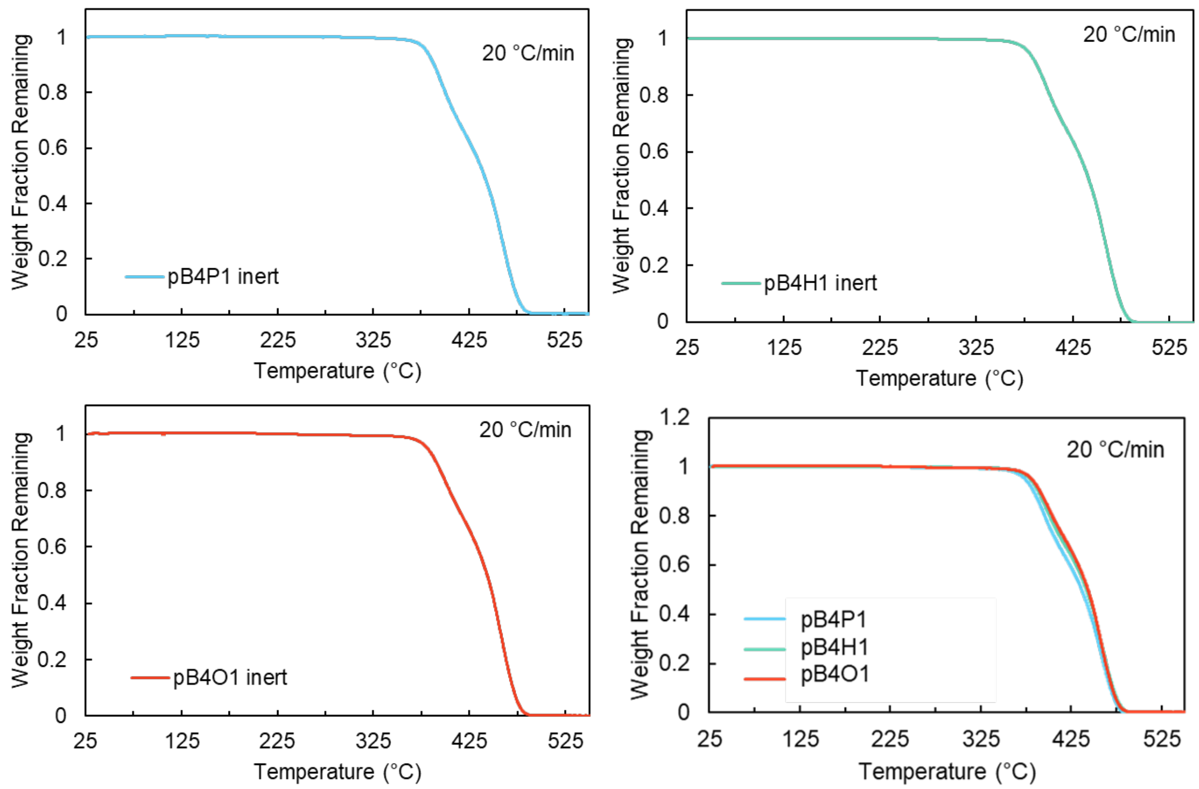


**Figure S36.** TGA measurements for pB4P1, pB4H1, and pB4O1 polymers under an inert (nitrogen) gas flow, taken at a ramp rate of 20 °C/min including an isothermal step at 105 °C for 15 min.

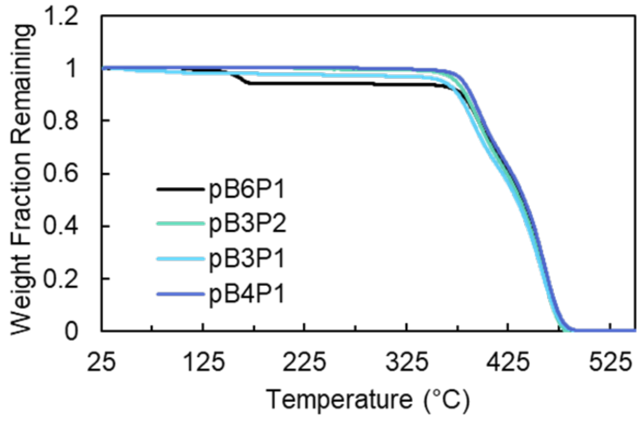


**Figure S37.** TGA measurements for pB6P1, pB3P2, pB3P1, and pB4P1 polymers under an inert (nitrogen) gas flow, taken at a ramp rate of 20 °C/min including an isothermal step at 105 °C for 15 min.

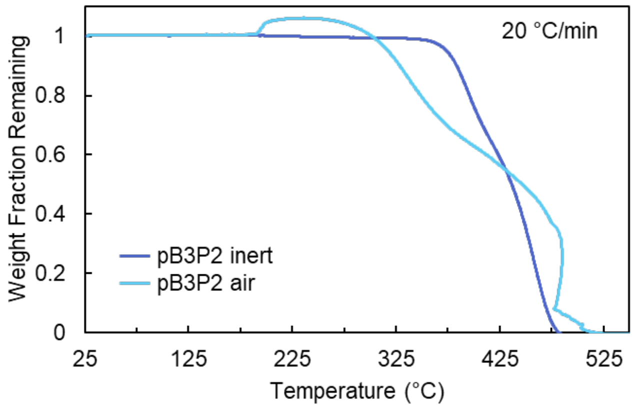


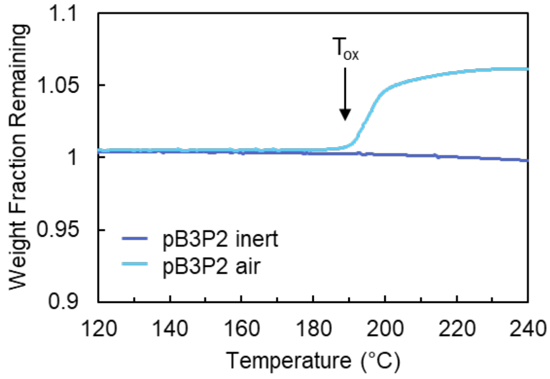


**Figure S38.** TGA measurements for pB3P2 polymers, taken at a ramp rate of 20 °C/min under an inert (nitrogen) gas flow or under air flow. Oxidation ($T_{ox}$) is noted as an increase in mass when run in an air environment compared to inert.

## S6. Calorimetric Characterization

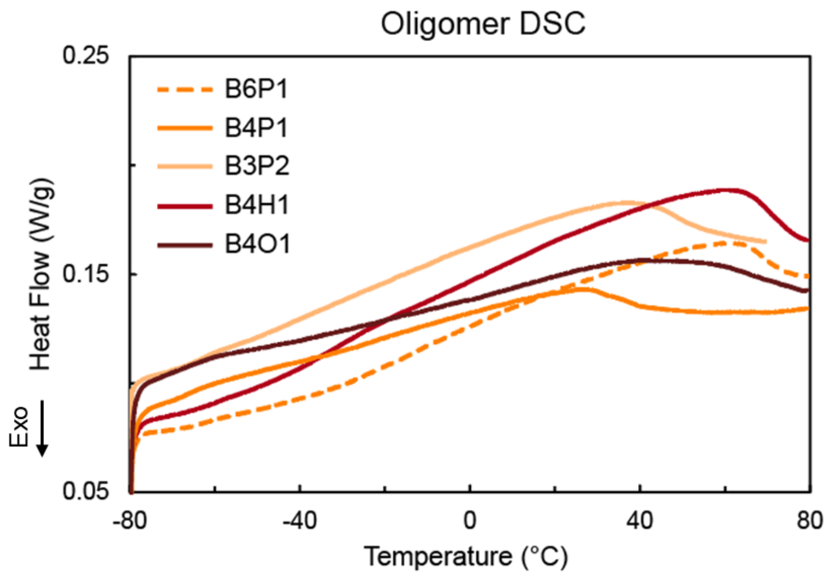


**Figure S39.** DSC measurements of B6P1, B4P1, B3P2, B4H1, and B4O1 oligomers, showing broad melting peaks between ~20-70 °C.

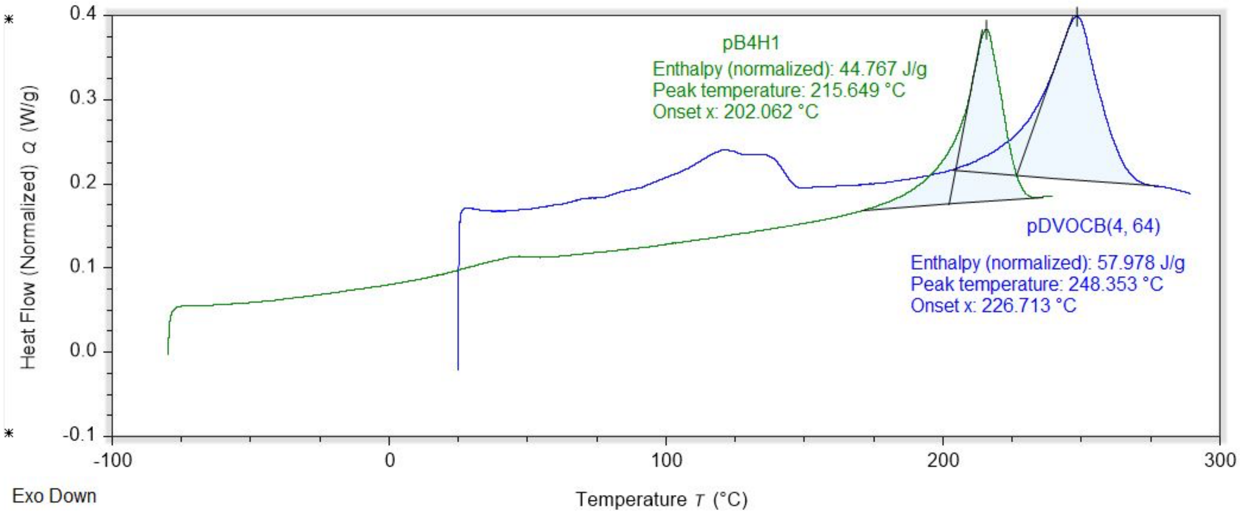


**Figure S40.** Melting enthalpy comparison of pB4H1 sample and pDVOCB(4, 64) sample, showing a melting enthalpy of 44.8 J/g for pB4H1 compared to 58.0 J/g for pDVOCB(4, 64).

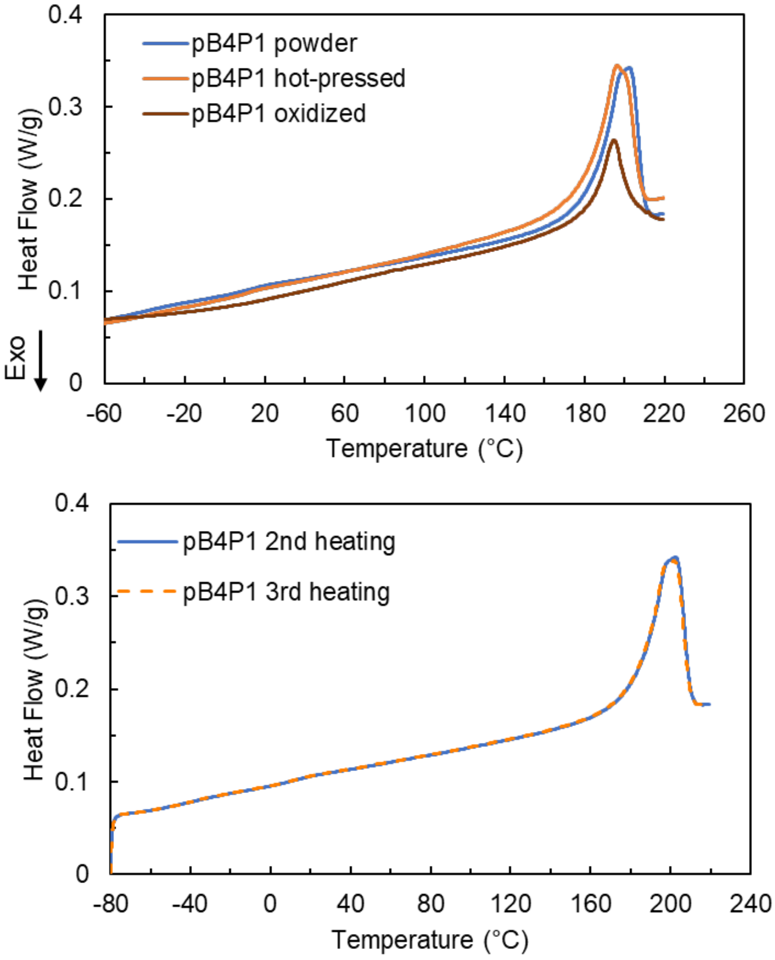

**Figure S41.** DSC stability tests. (Top) DSC comparison of second heating traces of pB4P1 sample from powder, after melt-pressing, vs an oxidized sample resulting from leaving a melt-pressed sample in air for > 6 months. The powder and freshly hot-pressed samples show negligible differences in the DSC second heating trace, whereas the oxidized sample shows a definite decrease in melting enthalpy due to oxidative crosslinking limiting crystallinity. (Bottom) Second vs third DSC heating cycles for pB4P1 polymer indicating stability over multiple heating and cooling cycles.

**Table S3.** Melting temperatures and melting enthalpy values for pB*n*X1.

| **Sample** | $T_{m,DSC}$ (°C) | $\Delta H_m$ (J/g) |
|---|---|---|
| pB6P1 | 223 | 42.5 |
| pB4P1 | 202 | 40.1 |
| pB3P2 | 191 | 38.7 |
| pB3P1 | 174 | 35.5 |
| pB6H1 | 225 | 52.2 |
| pB4H1 | 216 | 44.8 |
| pB6O1 | 215 | 47.5 |
| pB4O1 | 196 | 38.9 |

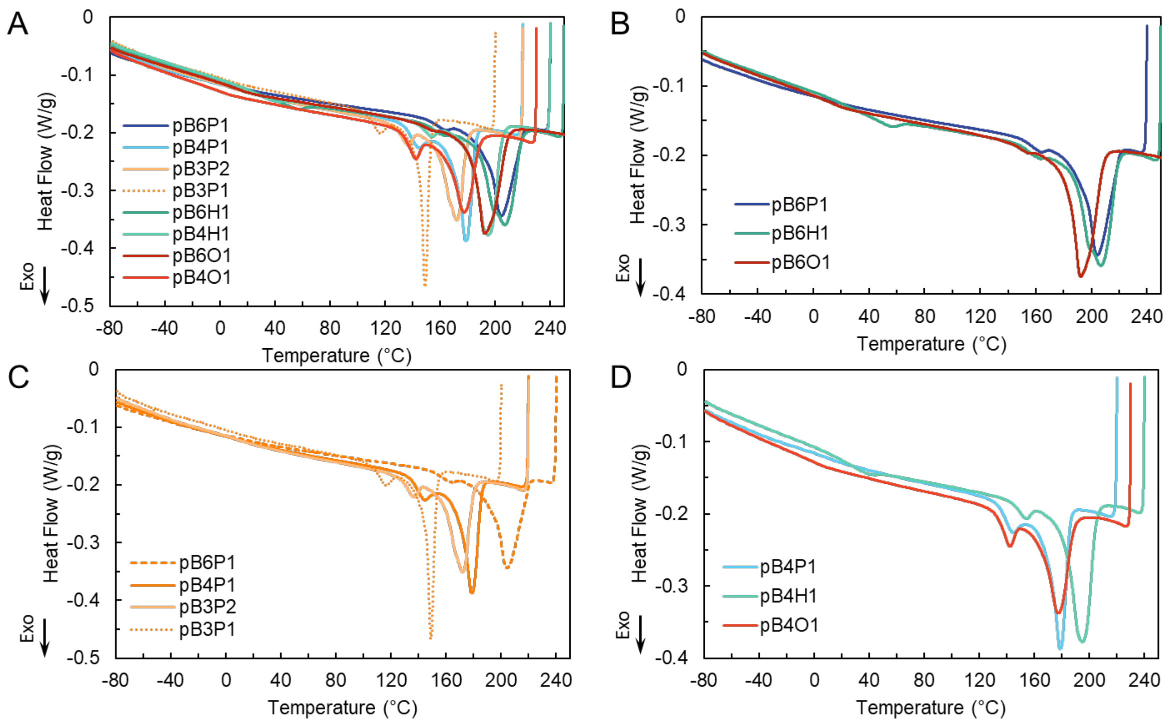


**Figure S42.** DSC cooling curves for pBnX1 polymers. Data shown is taken from the first cooling cycle at a ramp rate of 5 °C/min. (A) Cooling curves for full series of pBnX1, (B) cooling curves for pB6X1 series, (C) cooling curves for polymers created from BD:PD cross-oligomers, (D) cooling curves for pB4X1 series.

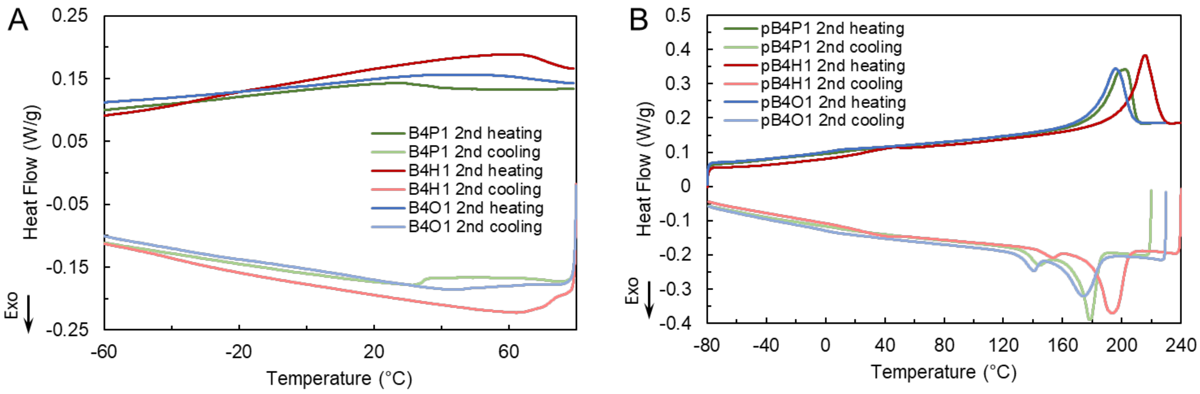


**Figure S43.** Representative DSC 2$^{nd}$ heating and 2$^{nd}$ cooling curve comparisons for (A) B4X1 oligomers and (B) pB4X1 polymers. The heating and cooling comparison and degree of undercooling shown is representative for other BnX1 oligomers and pBnX1 polymers.

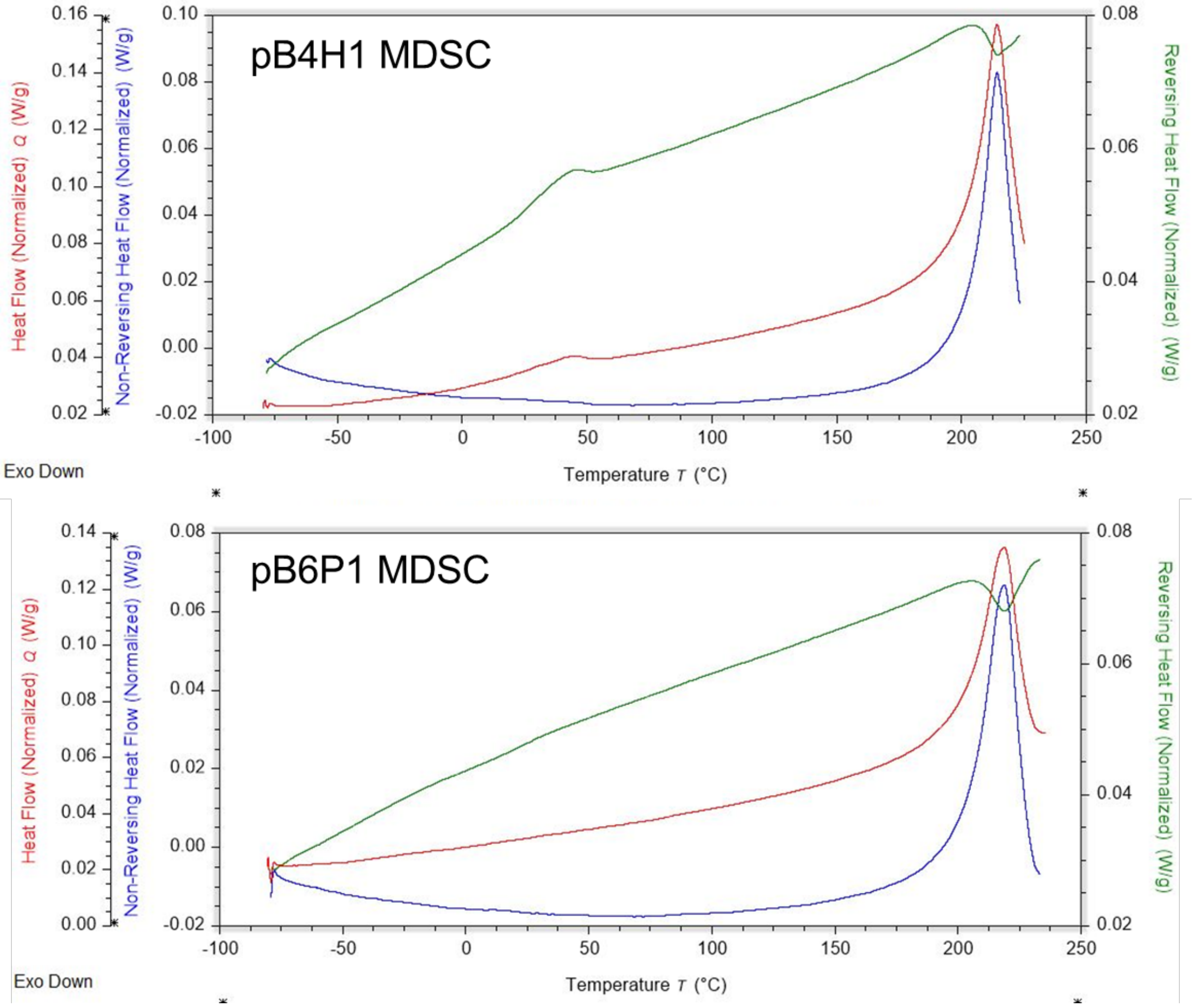


**Figure S44.** Modulated DSC (MDSC) second heating traces for pB4H1 (top) and pB6P1 (bottom) samples, showing total heat flow (red), non-reversing heat flow (blue), and reversing heat flow (green). The pB4H1 shows that the small endothermic peak around 40 °C remains in the reversing heat flow, not the non-reversing heat flow.

## S7. Experimental X-ray Scattering Measurements

### S7.1. Variable-temperature Wide-Angle X-ray Scattering

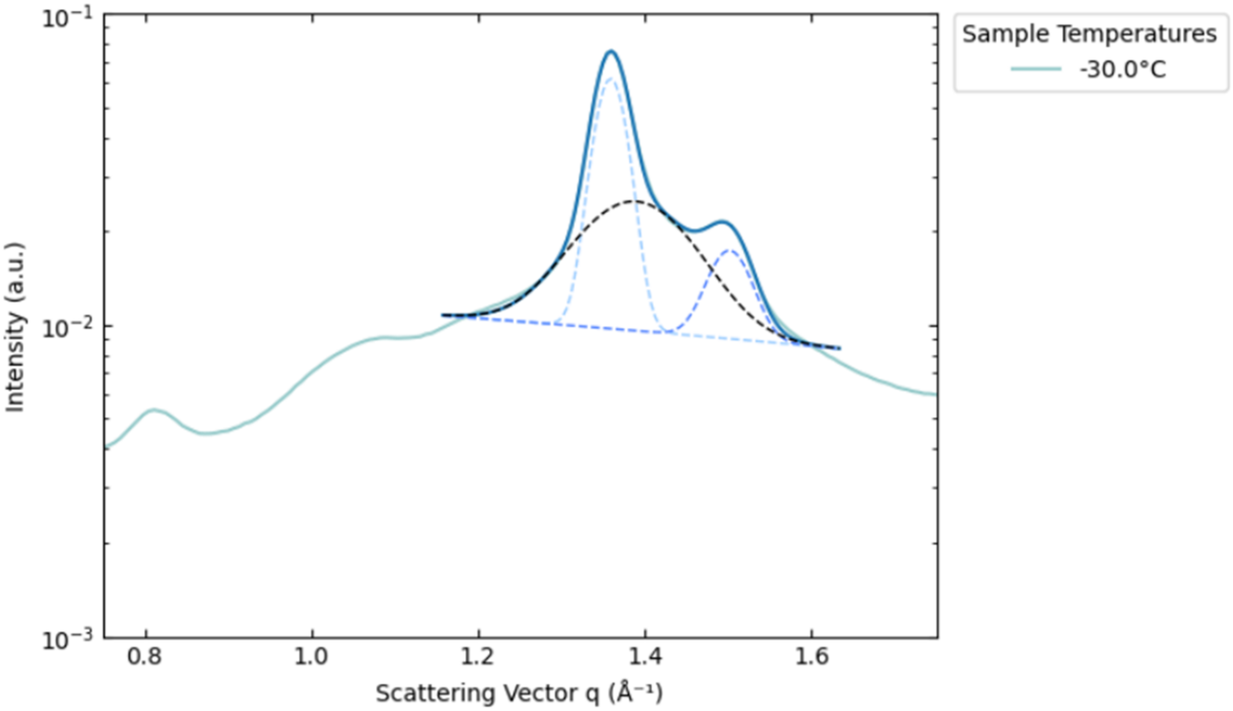


**Figure S45.** Example of WAXS peak fitting used to extract peak centers, shown for a pB4H1 sample. The data is fit to a convolution of three Gaussian peaks and a linear baseline (with two of the Gaussian peaks representing crystalline peaks, shown as blue dotted lines with the linear baseline added, and one representing the amorphous halo, shown as a black dotted line including the linear baseline).

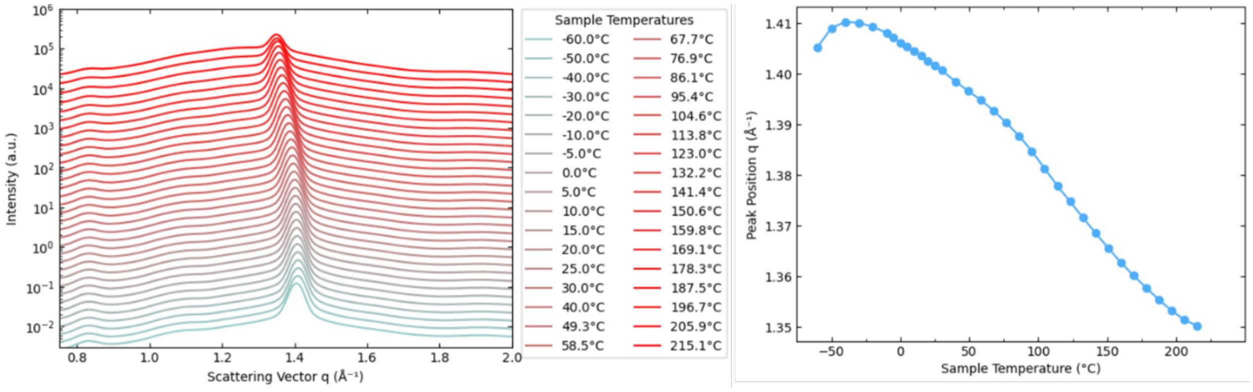


**Figure S46.** Variable-temperature wide-angle X-ray scattering results for pB4P1 polymer, showing I(q) vertically shifted with temperature (left) and the extracted primary scattering peak positions as a function of temperature (right).

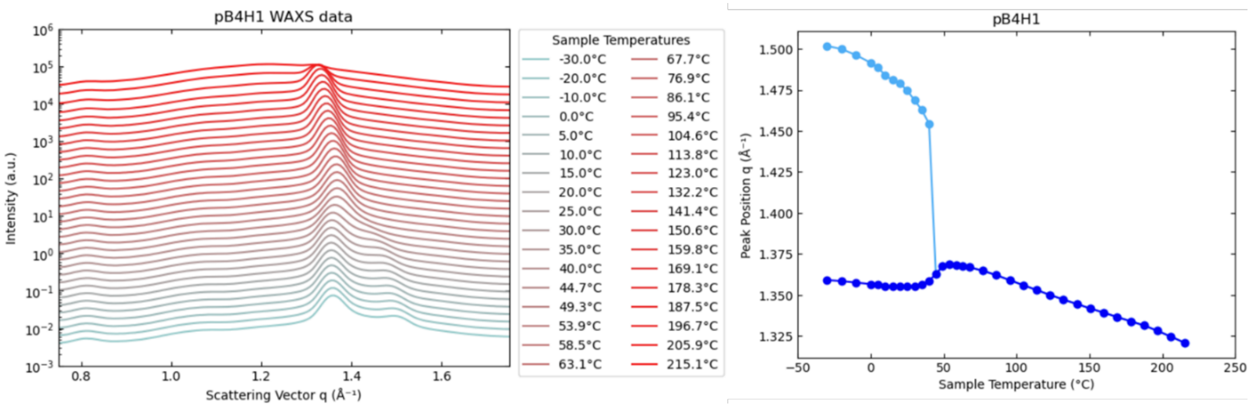


**Figure S47.** Variable-temperature wide-angle X-ray scattering results for pB4H1 polymer, showing I(q) vertically shifted with temperature (left) and the extracted primary scattering peak positions as a function of temperature (right) until melting.

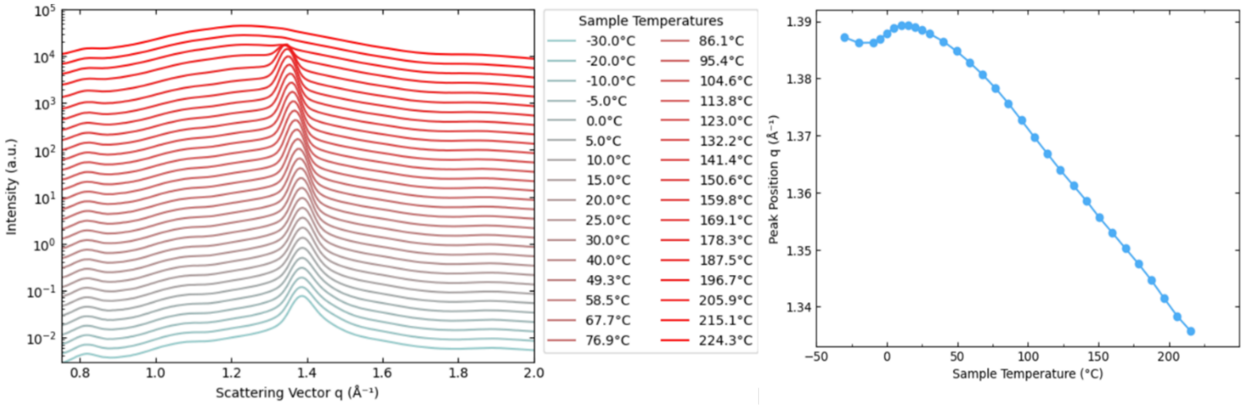


**Figure S48.** Variable-temperature wide-angle X-ray scattering results for pB4O1 polymer, showing I(q) vertically shifted with temperature (left) and the extracted primary scattering peak positions as a function of temperature (right) until melting.

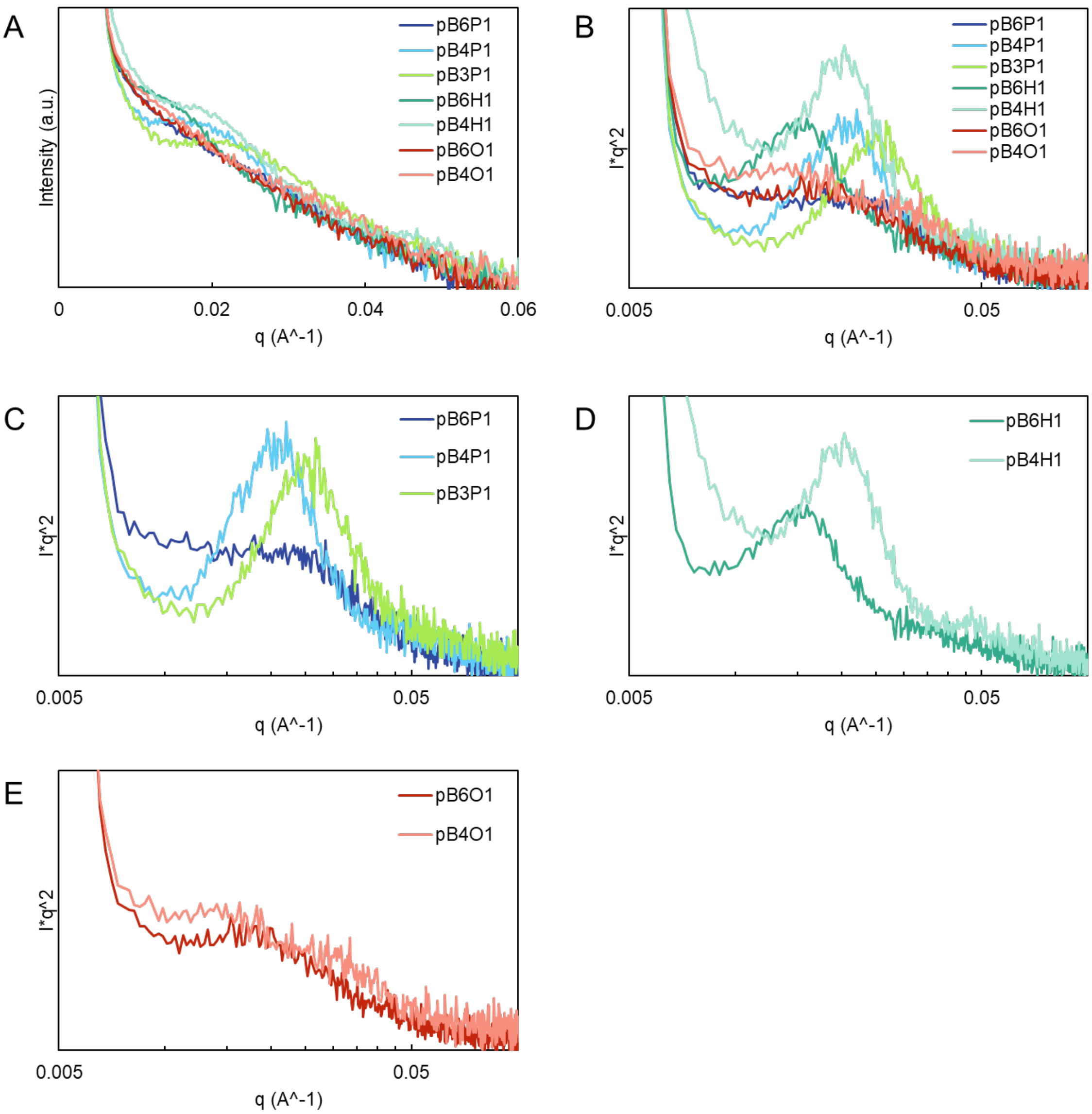


**Figure S49.** SAXS I(q) data for pBnX1 series, showing the peak related to the long period (corresponding to crystal thickness plus amorphous layer thickness for lamellar crystal structure). (A) Compiled SAXS data for pBnX1 polymers plotted as I(q). (B) Kratky plot ($I*q^2$) for pBnX1 compiled SAXS data. Kratky plot comparisons are shown for (C) pBnP1 polymers, (D) pBnH1 polymers, and (E) pBnO1 polymers. Comparison of pB4P1 and pB3P1 (or of pB6H1 and pB4H1) shows that the scattering peak shifts to smaller q (larger d spacing) with increasing comonomer ratio *n*.

### S7.2 Tensile-stage Wide-Angle X-ray Scattering

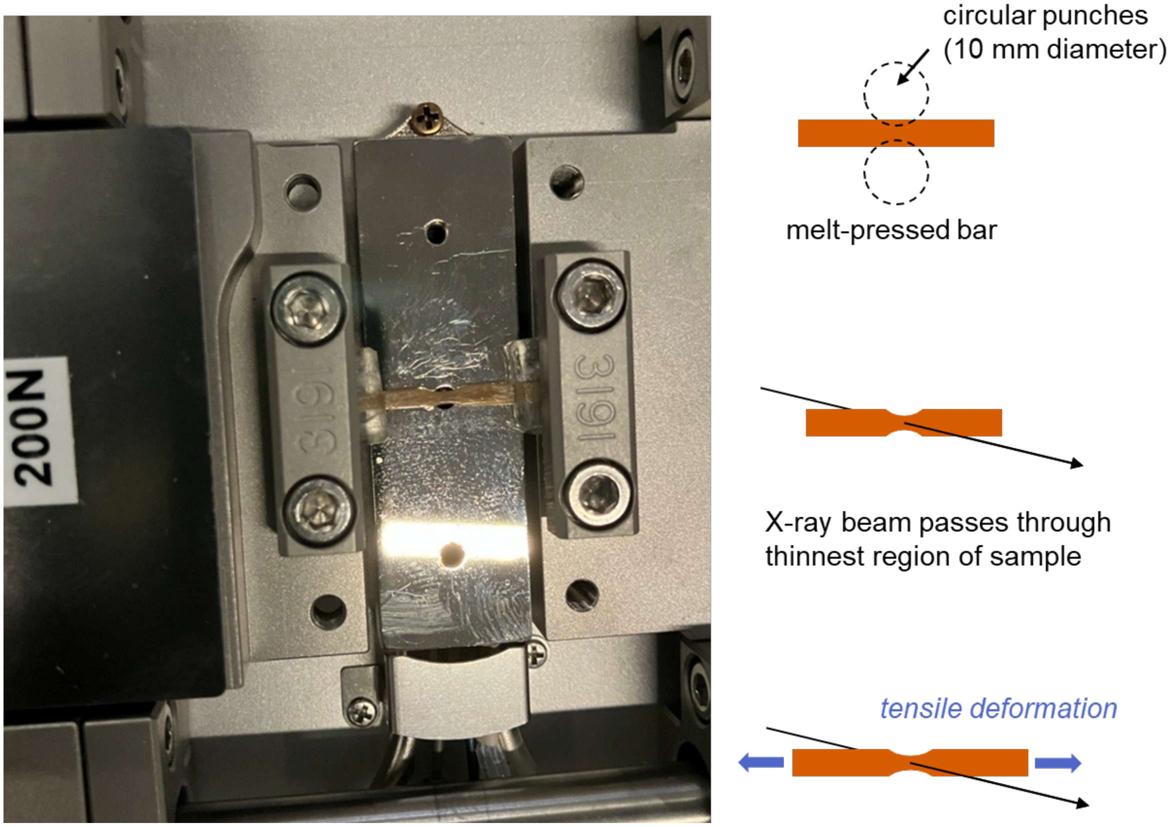


**Figure S50.** Photograph (left) and schematics (right) depicting sample mounting and preparation for tensile-stage X-ray measurements. A melt-pressed bar of polymer is prepared, then punched out on either side to create a rounded narrow section in the center of the sample, which is then aligned with the X-ray beam path and clamped on either side. This preparation is performed to encourage initial necking to occur in the path of the X-ray beam upon sample elongation.

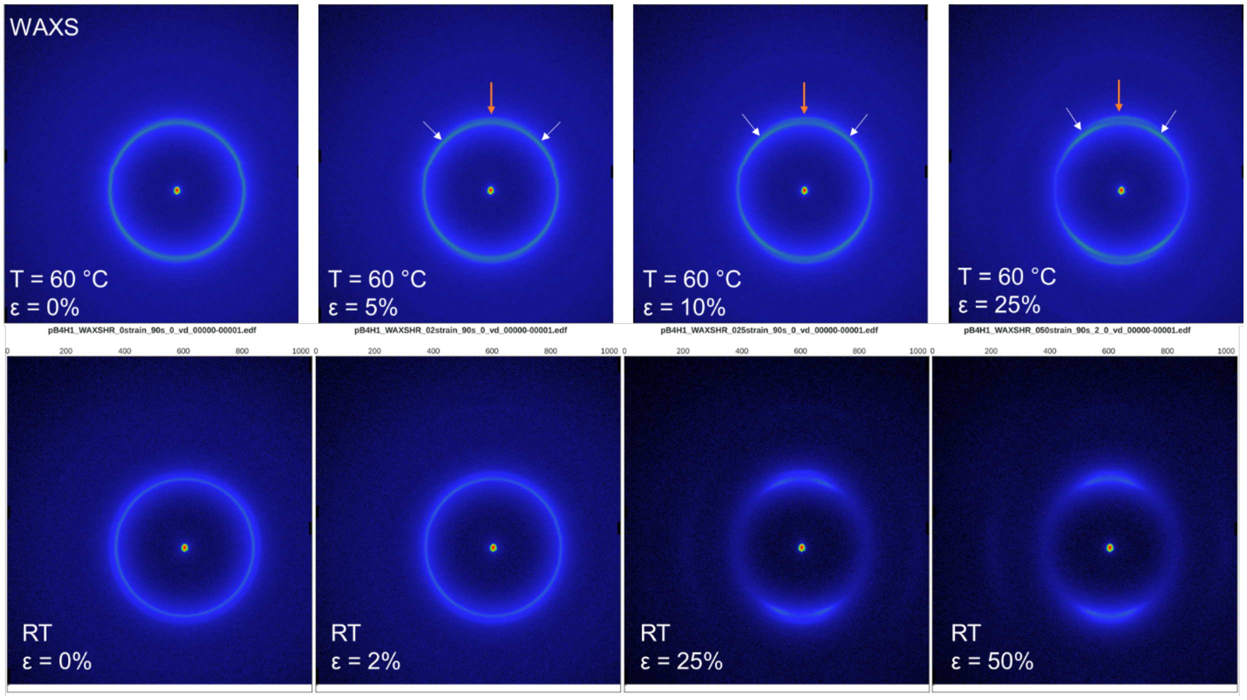


**Figure S51.** 2D tensile WAXS data for pB4H1 samples, including data taken while holding the sample at 60 °C (top row) and data taken during room temperature elongation (bottom row). For both rows, the strain direction is horizontal. In both cases, an inner crystalline peak develops orientation in a four-spot pattern with increasing strain, while an outer peak becomes oriented in a two-spot pattern perpendicular to the strain direction.

## S8. Mechanical Characterization

### S8.1 Dynamic Mechanical Analysis

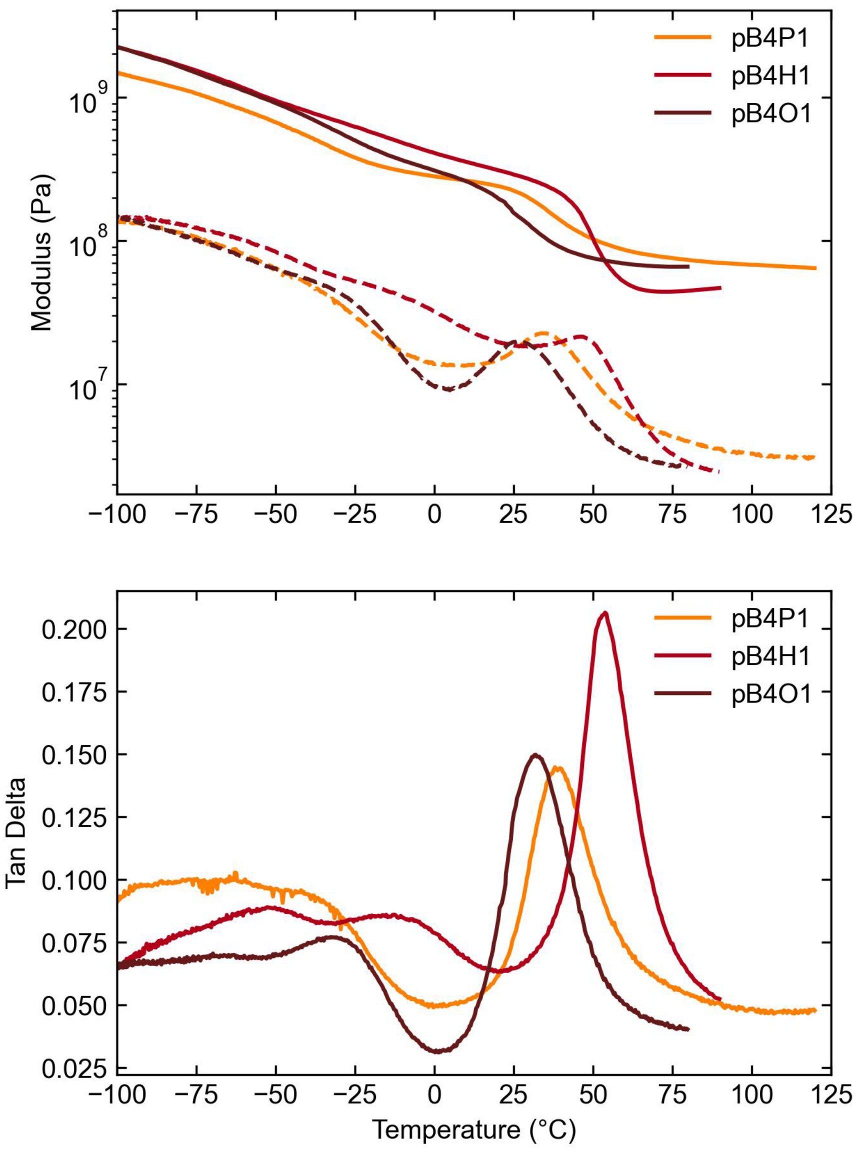


**Figure S52.** Storage modulus (top, solid lines), loss modulus (top, dashed lines), and tan delta (bottom) for pB4P1, pB4H1, and pB4O1 polymers. Data is collected from a DMA temperature sweep at a frequency of 1 Hz and heating rate of 3 °C/min.

**S8.2 Tensile Testing**

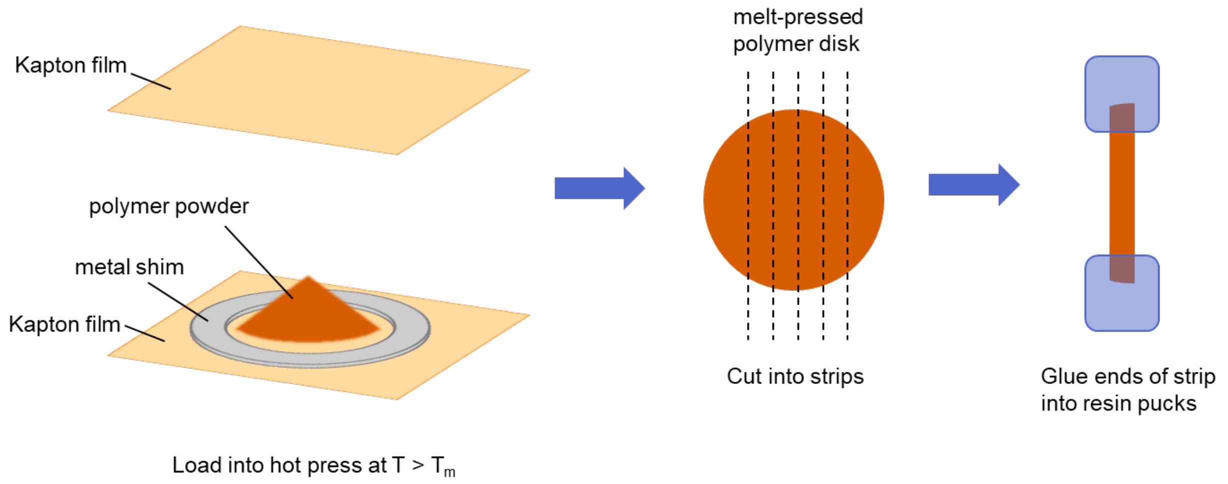


**Figure S53.** Schematic depicting sample preparation for Instron samples: first, loading a metal shim (20 mm inner diameter) with polymer powder between two sheets of Kapton before hot-pressing. After pressing, the sample disk is removed from the shim and sliced into several strips ~2 mm in width. The strips are then each mounted in resin pucks using superglue. During tensile testing, the resin pucks are clamped into the Instron grips to minimize sample damage from clamping. This procedure was followed to minimize the amount of material required for tensile testing.

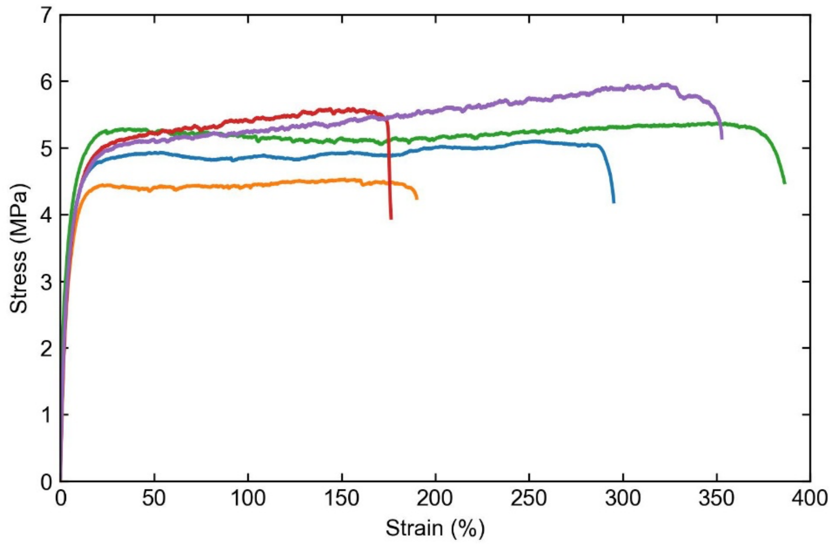


**Figure S54.** Instron mechanical testing results for pB4P1 polymer samples, performed at a strain rate of 3% engineering strain per min until failure.

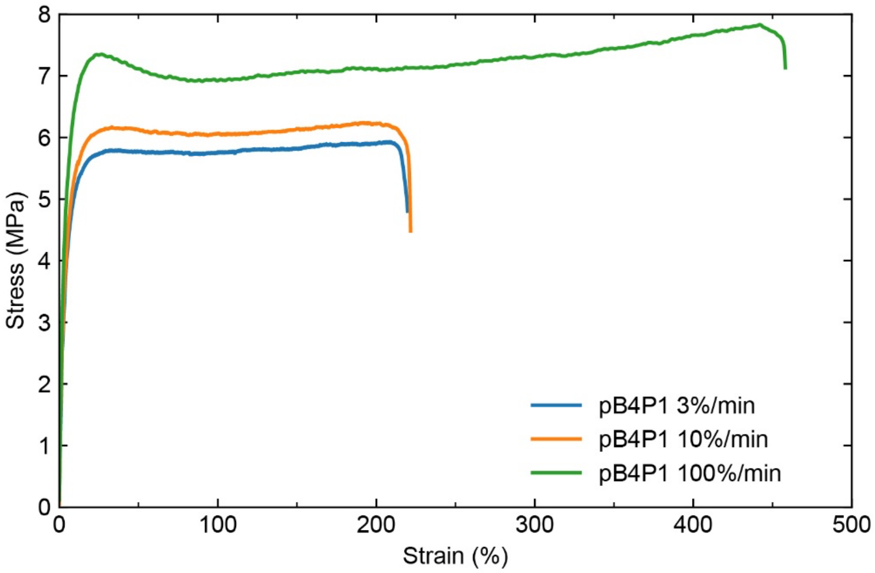


**Figure S55.** Strain rate dependence of pB4P1 mechanical behavior. Tensile testing results shown for pB4P1 samples taken at different strain rates (3, 10, and 100% engineering strain per min, as noted in legend). The three samples shown here were all prepared from the same melt-pressed disk.

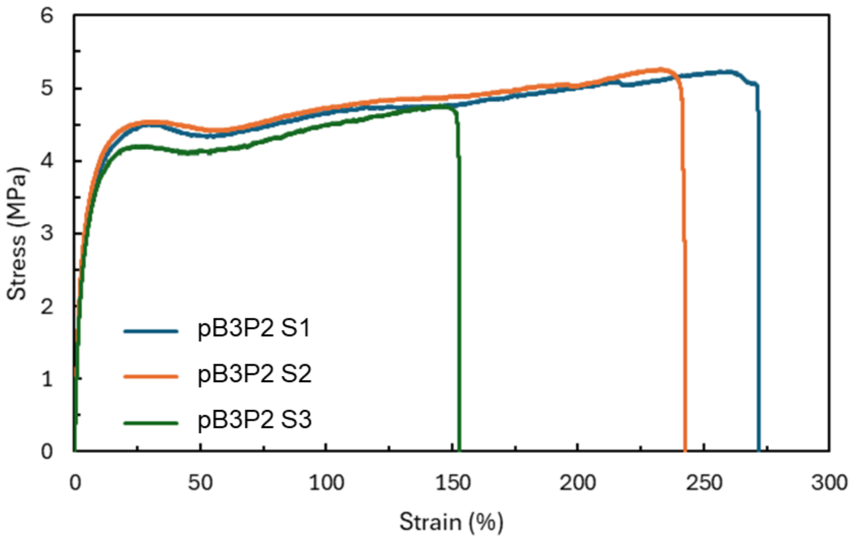


**Figure S56.** Instron mechanical testing results for pB3P2 polymer samples, performed at a strain rate of 3% engineering strain per min until failure.

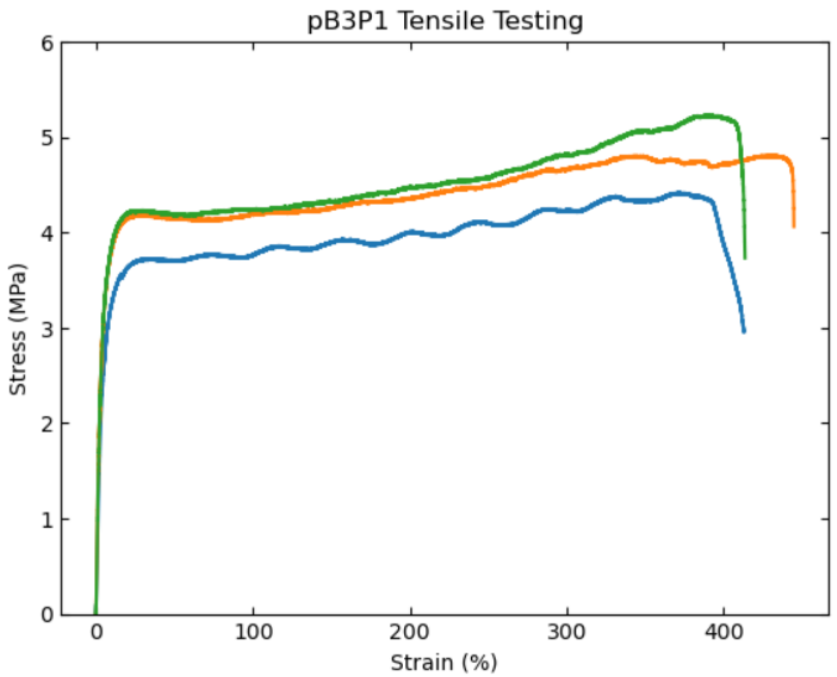


**Figure S57.** Instron mechanical testing results for pB3P1 polymer samples, performed at a strain rate of 3% engineering strain per min until failure. The oscillations in the blue data are artifacts and not representative of the mechanical behavior of the sample.

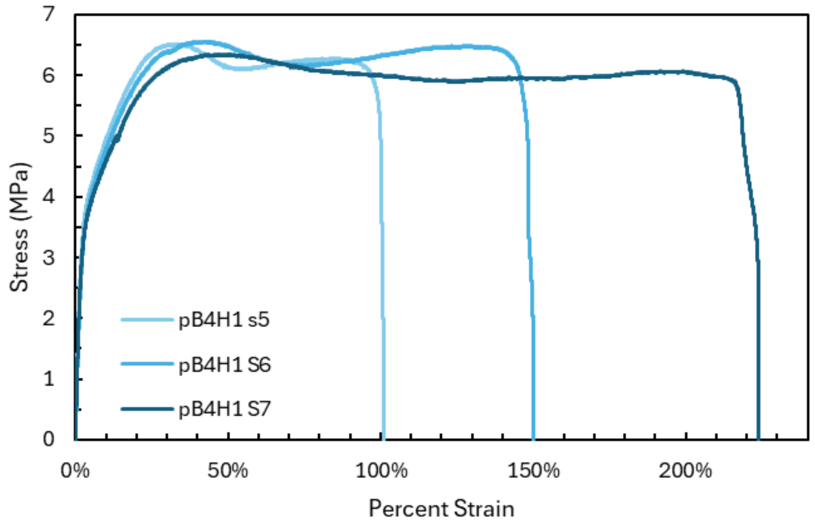


**Figure S58.** Instron mechanical testing results for pB4H1 polymer samples, performed at a strain rate of 3% engineering strain per min until failure.

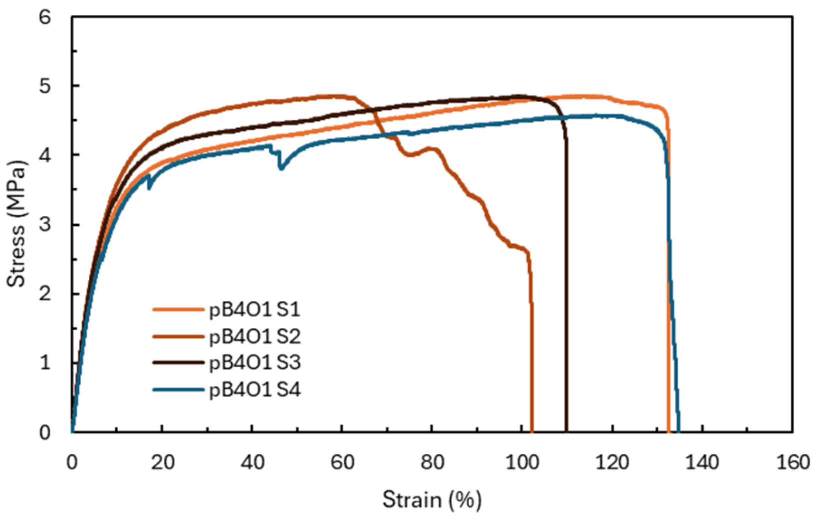


**Figure S59.** Instron mechanical testing results for pB4O1 polymer samples, performed at a strain rate of 3% engineering strain per min until failure. Due to premature failure, sample 2 was excluded from following toughness and modulus calculations.

**Table S4.** Toughness values for pB*n*X1 polymers compared to pDVOCB

| **Sample** | **Average** (MJ/m^3) | **Standard Deviation** (MJ/m^3) |
|---|---|---|
| pB4P1 | 14.2 | 5.4 |
| pB3P1 | 18.1 | 1.8 |
| pB4H1 | 9.5 | 3.5 |
| pB4O1 | 5.2 | 0.5 |
| pDVOCB(5,40) | 1.2 | - |

Note: pDVOCB(5,40) toughness is calculated from mechanical data from literature for comparison.[4]

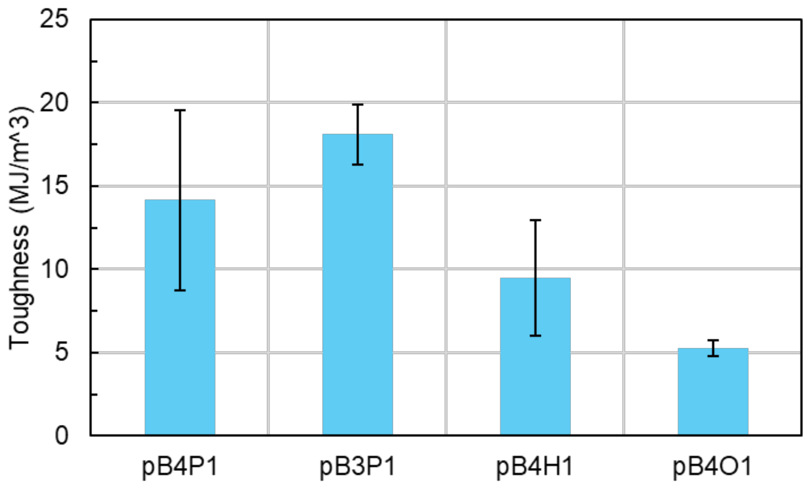


**Figure S60.** Toughness values for pB*n*X1 polymers calculated from the integrated area underneath stress strain curves (numerical data shown in **Table S4**). The error bars represent the standard deviation over at least three samples.

**Table S5.** Young's modulus values for pB*n*X1 polymers

| Sample | **Average** (MPa) | **Standard Deviation** (MPa) |
|---|---|---|
| pB4P1 | 120.9 | 10.8 |
| pB3P1 | 105.3 | 14.9 |
| pB4H1 | 191.8 | 2.7 |
| pB4O1 | 57.6 | 6.7 |

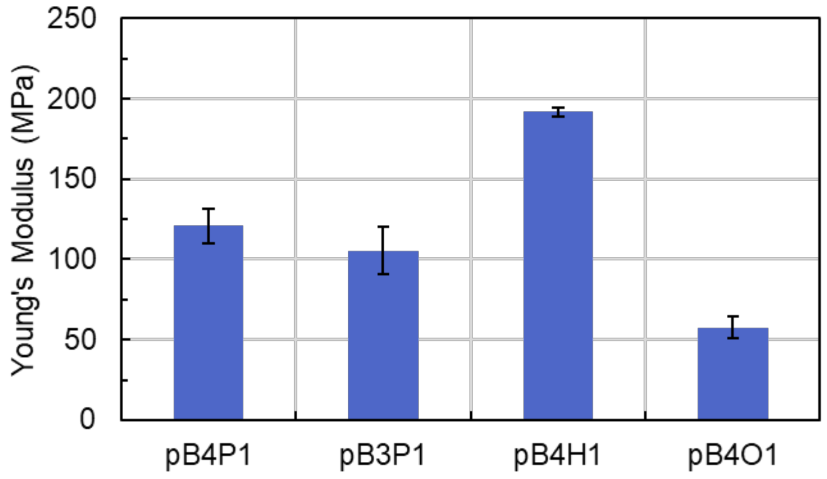


**Figure S61.** Young's modulus values for pB*n*X1 polymers calculated from the initial slope of stress strain curves (numerical values shown in **Table S5**). The error bars represent the standard deviation over at least three samples.

## S9. Simulation Details

The simulations include pB4P1, pB4H, pB4H'1, pB4O1, pB4N1, pB4D1, and pDVOCB(4,*m*) polymers. For each polymer, three independent amorphous systems and one crystal system were generated to estimate the uncertainty of amorphous system properties. The initial amorphous systems are prepared by randomly placing six ~30 kDa polymer chains into a 100 Å simulation box, resulting in ~33,000 atoms in total. Tight ring configurations were used to avoid chains going through the center of rings in the initial system. These tight rings will expand to normal size during the energy minimization process when the equilibration simulation starts. Then the systems were equilibrated by 0.1 ns NVT, 1 ns NPT, 21-steps,[17] and 2 ns NPT simulation. The final configurations were used for temperature ramp simulations: 1 ns 300K-400K, 4 ns 400K NPT, 30 ns 400K-100K NPT, and 40 ns 100K-500K NPT simulation. The last heating processes were used to collect density data and calculate glass transition temperature through hyperbola fit.[18]

The initial crystal system was prepared in two steps: (1) generate straight, random sequence polymer chains and connect their two ends through the periodic boundary in the y direction, and (2) arrange the chains into hexagonal packing. In this way, we created crystal structures with 196 infinitely long polymer chains and ~60,000 atoms in total. Then the systems were simulated by 0.1 ns 300K NVT, 10 ns 300K NPT, 2 ns 300K-100K NPT, 1 ns 100K NPT, 40 ns 100K-500K, and 40 ns 500K-100K. The two-peak XRD feature of pB4H1 was not found in the temperature ramp. To help the system build crystal structure, we deformed the simulation box in the z-direction while keeping the barostat on in the x- and y-directions. This deformation forces the chains to align parallel or perpendicular to the z-direction, which may be stable after turning on the barostat in the z-direction. The original simulation boxes were around 61 Å in z-direction and the deformed simulation boxes were (57, 58, 59, 60, 61, 62, 63, 64, 65, 66) Å. After the deformation, the systems showed orientational order and the two-peak feature in XRD, as shown in **Figure S62**. Then the configurations after deformation were equilibrated by 15 ns 250K NPT simulations. Only the compressed pB4H1 kept the two-peak feature in XRD after equilibration, which indicates that other polymers could not form a stable two-peak crystal structure, and agrees with the crystal structure observed for pB4H1 in experiments. The final configurations were then simulated by 15 ns 250K-100K NPT and 40 ns 100K-500K NPT. The last heating processes were used to collect density data and calculate transition temperature from density through a hyperbolic fit. The configurations during the last heating processes are used as initial configurations for NPT simulations to calculate the rotational autocorrelation functions.

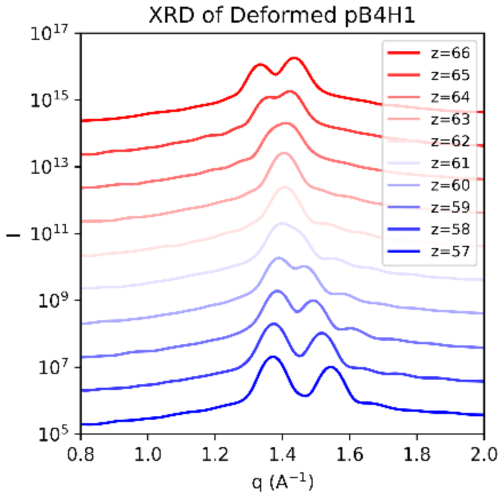


**Figure S62.** The XRD of deformed pB4H1 crystal systems.

## S9.1. Simulation Results of XRD

Simulated scattering intensities were obtained based on the virtual diffraction algorithm.[19] The simulation XRD results of all the crystal polymer systems are shown in **Figure S63** and **Figure S64**.

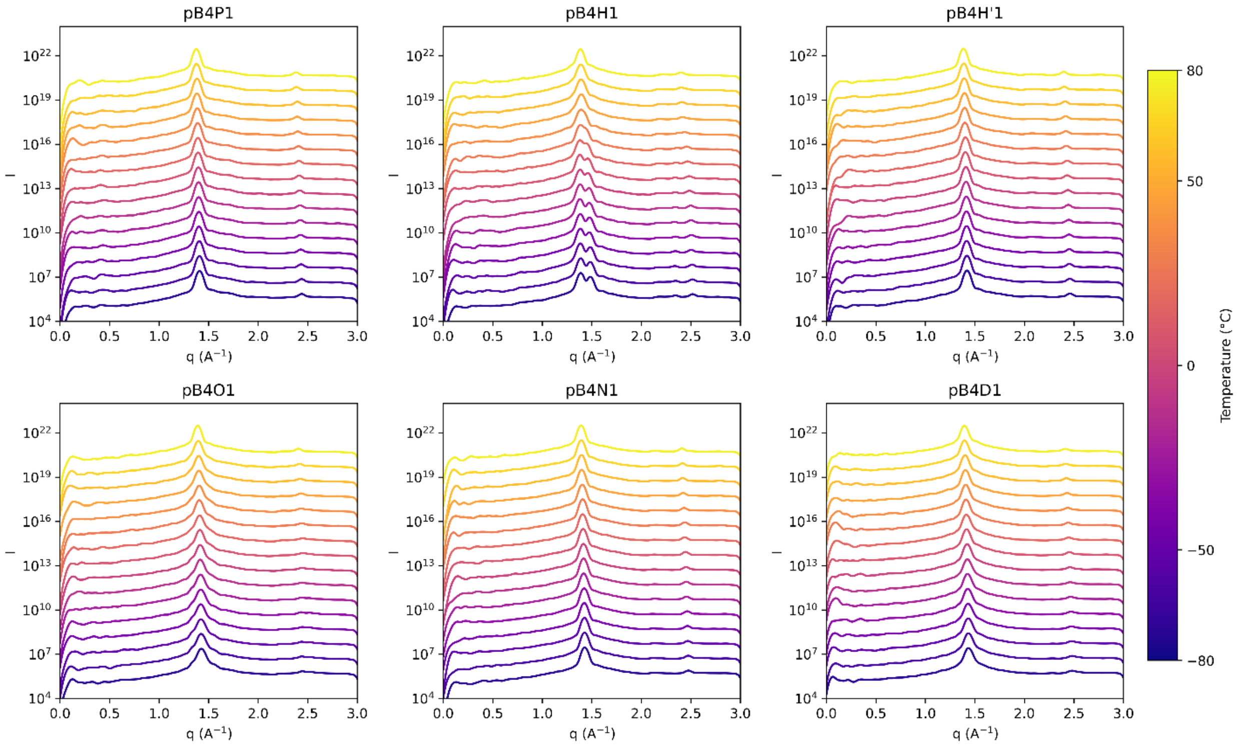


**Figure S63.** The XRD results of pB4X1.

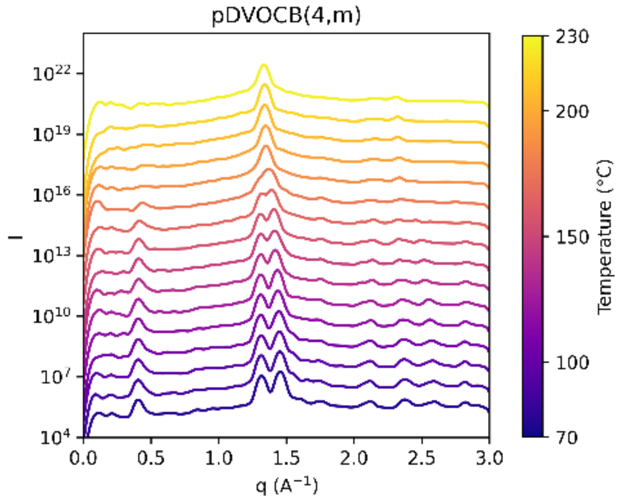


**Figure S64.** The XRD result of pDVOCB(*4,m*)

### S9.2 Simulation Results of Order Parameter

Two order parameters are used to measure the orientational order in the whole chain and single ring level. The definition of ring orientation is the same as our previous work.[6] For a specific ring, denoting the carbons as $i, j$, $k$, and $l$ with $j$ and $l$ as the central carbons, then the ring orientation is $\vec{\alpha} = \sum_{a\in\{i,k\}} \sum_{b\in\{j,l\}} (r_b - r_a)$. For a specific chain, the chain orientation is $\vec{\beta} = \sum \mathrm{sign}(\hat{x} \cdot \vec{\alpha})\vec{\alpha}$.

The chain parallel order parameter for chain $i$ is:

$$p_i^c = \prod_{j\in\mathcal{N}(i)} H\left(\cos\theta_{ij}^{(\beta)} - \cos\theta_{\max}\right)$$

Here, $\cos\theta_{ij}^{(\beta)} \equiv \left(\vec{\beta_i} - \vec{\beta_i} \cdot \hat{y}\hat{y}\right) \cdot \left(\vec{\beta_j} - \vec{\beta_j} \cdot \hat{y}\hat{y}\right)$ is the angle between chain $i$ and $j$ in the $xy$ plane. $\mathcal{N}(i)$ denotes the local neighborhood of $i$ (i.e., the six nearest-neighbor chains), $H(\cdot)$ denotes the Heaviside step function, and $\theta_{\max} = \pi/4$ is the threshold angle. The chain parallel order parameter for the system is:

$$P^c = \frac{1}{N_\mathrm{c}} \sum_i^{N_\mathrm{c}} p_i^c$$

Here, $N_\mathrm{c}$ is the number of chains in the system.

The ring parallel order parameter for ring $k$ on chain $i$ is:

$$p_k^r = \prod_{l\in j,\min\left(r_{k,l}\right), j\in\mathcal{N}(i)} H\left(\cos\theta_{kl}^{(\alpha)} - \cos\theta_{\max}\right)$$

Here, $l$ is the ring on chain $j$ that is closest to ring $i$. The ring parallel order parameter for the system is:

$$P^r = \frac{1}{N_\mathrm{r}} \sum_i^{N_\mathrm{r}} p_i^r$$

Here, $N_\mathrm{r}$ is the number of rings in the system. Based on the threshold, the order parameter for a system with random orientations is $(1/2)^6 \sim 0.0156$.

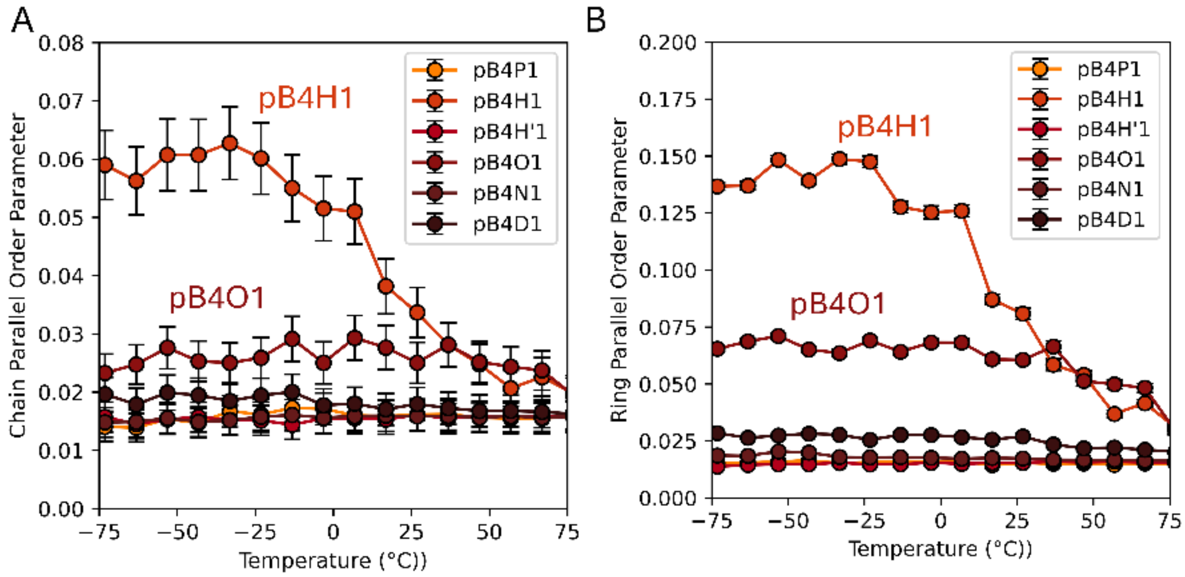


**Figure S65.** The chain and ring parallel order parameter change with temperature.

We calculate two orientation order parameters for pB4X1 materials from simulations to examine the crystal structure. pB4H1 has relatively strong parallel order at both the single-ring and whole-chain levels, which suggests a monoclinic structure and agrees with the two peaks observed in scattering results. Other methylene-modified polymers with even methylene spacers, pB4O1 and pB4D1, have much smaller chain level parallel order parameters, which indicates that there is essentially no large-scale orientational order across the system and a hexagonal structure is preferred at low temperature. In pB4O1, small but non-negligible ring level order parameters are found, which suggests that it has weak locally ordered structures aligning with the larger tail at the second peak position in the scattering results (**Figure S65**). All simulated odd methylene-modified polymers (i.e., pB4P1, pB4H'1, and pB4N1) show order parameters close to random orientation statistics, which indicates that they lack orientational order and remain in hexagonal structures at low temperatures. These results suggest that pB4X1 samples containing an even number of methylene spacers form more ordered structures at low temperatures, which in turn results in higher $T_r$ values. Additionally, the hexagonal crystal phase in polymers other than pB4H1 confirms that most pB4X1 polymers transition from a hexagonal crystal to a hexagonal rotator phase at the $T_r$, resulting in a single scattering peak throughout the $T_r$ transition.

### S9.3 Simulation Results of ACF

The rotational autocorrelation function (ACF) is calculated by

$$ACF(t) = \langle \vec{\alpha}(0) \cdot \vec{\alpha}(t) \rangle \approx (1 - A)\exp[-(t/t_1)] + A\exp\left[-(t/t_2)^{\beta}\right]$$

Here $A$, $t_1$, $t_2$, and $\beta$ are fitting parameters.

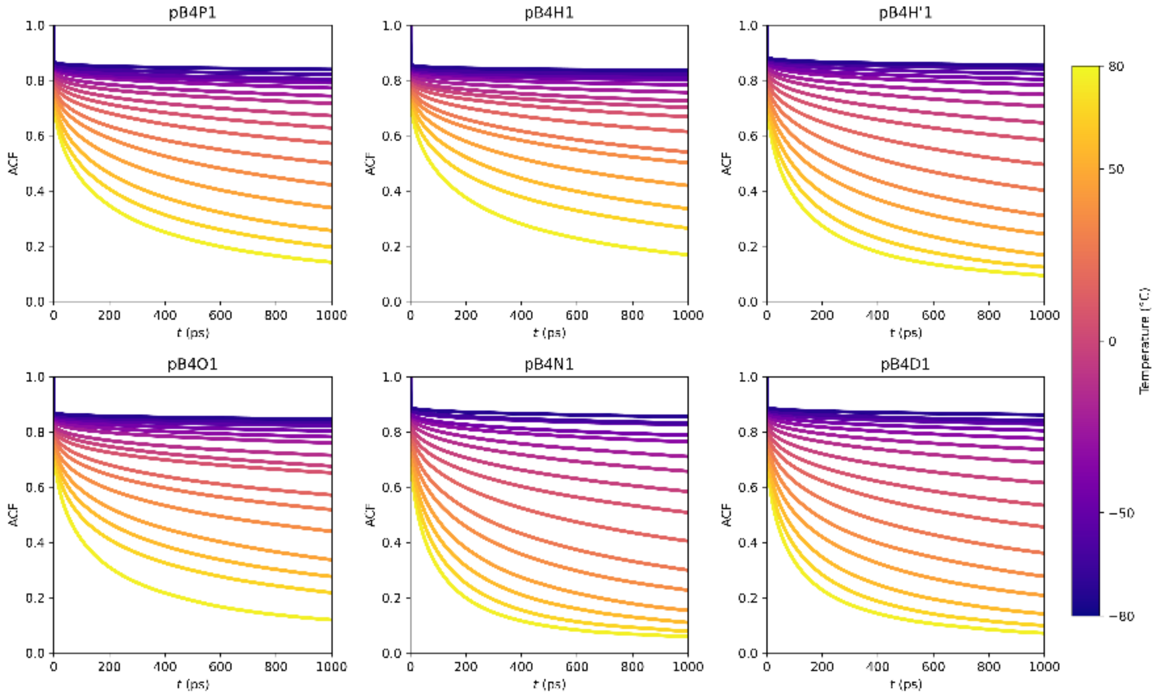


**Figure S66.** The ACF of all the pB4X1 polymers.

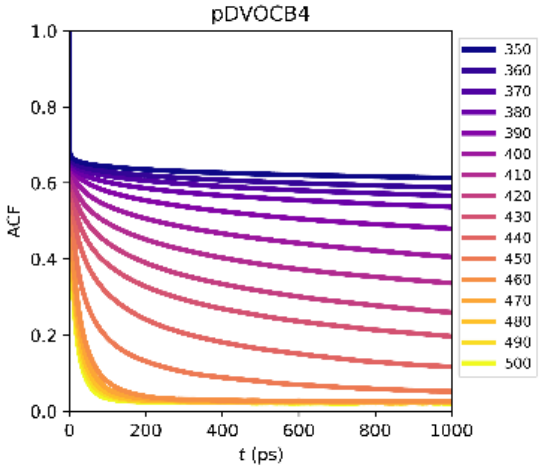


**Figure S67.** The ACF of the pDVOCB(*4,m*) polymers.

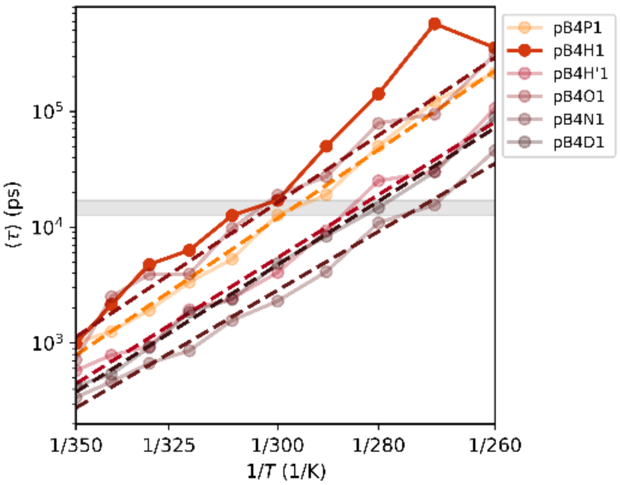


**Figure S68.** The fitting of average rotational relaxation time and temperature. The simulation data is shown as solid line and dot, the fitting is shown as dashed line, and the $T_r$ range is shown as gray shadow.

To determine the $T_r$ from ACF of pB4X1 materials, we performed a linear fitting between $\ln(\langle\tau\rangle)$ and $1/T$. The hypothesis is that because of the hexagonal structure, rings have favored orientations, and the rotation process is controlled by the energy barrier, so $\langle\tau\rangle = A\exp(-E_b/k_BT)$, in which $A$ is a constant and $E_b$ is the height of the energy barrier. After rearrangement, $\ln(\langle\tau\rangle) = -E_b/k_BT + ln(A)$; we fit $\langle\tau\rangle$ between 260K and 350K to this model. The simulation data show good agreement with the model with $R^2 > 0.978$. From the fitted model, we calculate the temperature ranges of different pB4X1 materials, in which their $\langle\tau\rangle$ match with pB4H1's $\langle\tau\rangle$ during the rotator phase transition. The centers of these ranges are used as the values of $T_r$ from ACF, and the half widths of these ranges are used as uncertainties. We also note that the fitted $E_b$ of different pB4X1 materials are around 10 kcal/mol and show an odd-even effect: pB4X1 materials with even methylene spacers have relatively higher $E_b$ during the ring rotation process.

## S9.4 Simulation Results of MSD

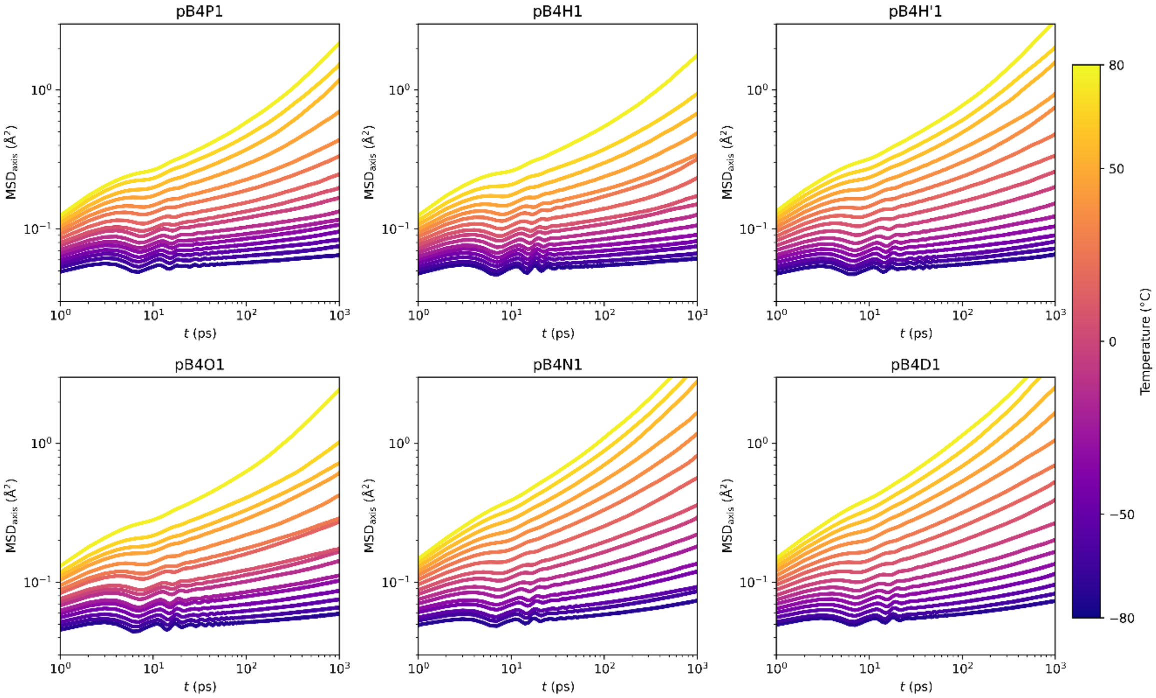


**Figure S69** The MSD along chain direction of all the pB4X1 polymers.

## References


(1) Pangborn, A. B.; Giardello, M. A.; Grubbs, R. H.; Rosen, R. K.; Timmers, F. J. Safe and Convenient Procedure for Solvent Purification. *Organometallics* **1996**, *15* (5), 1518–1520.

(2) Russell, S. K.; Darmon, J. M.; Lobkovsky, E.; Chirik, P. J. Synthesis of Aryl-Substituted Bis(Imino)Pyridine Iron Dinitrogen Complexes. *Inorg. Chem.* **2010**, *49* (6), 2782–2792.

(3) Russell, S. K.; Hoyt, J. M.; Bart, S. C.; Milsmann, C.; Stieber, S. C. E.; Semproni, S. P.; DeBeer, S.; Chirik, P. J. Synthesis, Electronic Structure and Reactivity of Bis(Imino)Pyridine Iron Carbene Complexes: Evidence for a Carbene Radical. *Chem. Sci.* **2014**, *5* (3), 1168–1174.

(4) Nie, C.; Maguire, S. M.; Zheng, C. W.; Mohadjer Beromi, M.; Register, R. A.; Priestley, R. D.; Davidson, E. C.; Chirik, P. J. A Butadiene-Derived Semicrystalline Polyolefin with Two-Tiered Chemical Recyclability. *Chem* **2024**, *10* (2), 698–712.

(5) Black, D. R.; Mendenhall, M. H.; Brown, C. M.; Henins, A.; Filliben, J.; Cline, J. P. Certification of Standard Reference Material 660c for Powder Diffraction. *Powder Diffr.* **2020**, *35* (1), 17–22.

(6) Zhang, H.; Maguire, S. M.; Nie, C.; Priestley, R. D.; Chirik, P. J.; Register, R. A.; Davidson, E. C.; Webb, M. A. Rotator Phases in Chemically Recyclable Oligocyclobutanes. *Chem. Mater.* **2024**, *36* (23), 11596–11605.

(7) Archer, W. R.; Nie, C.; Maguire, S. M.; Sundar, S. V.; Priestley, R. D.; Davidson, E. C.; Chirik, P. J.; Register, R. A. Synthesis and Tunable Properties of Chemically Recyclable Multiblock Copolymers via Tandem Olefin Metathesis Polymerizations. *Macromolecules* **2025**, *58* (8), 4272–4280.

(8) Thompson, A. P.; Aktulga, H. M.; Berger, R.; Bolintineanu, D. S.; Brown, W. M.; Crozier, P. S.; in 't Veld, P. J.; Kohlmeyer, A.; Moore, S. G.; Nguyen, T. D.; Shan, R.; Stevens, M. J.; Tranchida, J.; Trott, C.; Plimpton, S. J. LAMMPS - a Flexible Simulation Tool for Particle-Based Materials Modeling at the Atomic, Meso, and Continuum Scales. *Comput. Phys. Commun.* **2022**, *271*, 108171.

(9) Hockney, R. W.; Eastwood, J. W. *Computer Simulation Using Particles*; CRC Press: Boca Raton, 2021.

(10) Hoover, W. G. Canonical Dynamics: Equilibrium Phase-Space Distributions. *Phys. Rev. A* **1985**, *31* (3), 1695–1697.

(11) Seo, B.; Lin, Z.-Y.; Zhao, Q.; Webb, M. A.; Savoie, B. M. Topology Automated Force-Field Interactions (TAFFI): A Framework for Developing Transferable Force Fields. *J. Chem. Inf. Model.* **2021**, *61* (10), 5013–5027.

(12) Hoyt, J. M.; Schmidt, V. A.; Tondreau, A. M.; Chirik, P. J. Iron-Catalyzed Intermolecular [2+2] Cycloadditions of Unactivated Alkenes. *Science* **2015**, *349* (6251), 960–963.

(13) Mohadjer Beromi, M.; Kennedy, C. R.; Younker, J. M.; Carpenter, A. E.; Mattler, S. J.; Throckmorton, J. A.; Chirik, P. J. Iron-Catalysed Synthesis and Chemical Recycling of Telechelic 1,3-Enchained Oligocyclobutanes. *Nat. Chem.* **2021**, *13* (2), 156–162.

(14) Kennedy, C. R.; Zhong, H.; Joannou, M. V.; Chirik, P. J. Pyridine(Diimine) Iron Diene Complexes Relevant to Catalytic [2+2]-Cycloaddition Reactions. *Adv. Synth. Catal.* **2020**, *362* (2), 404–416.

(15) Russell, S. K.; Lobkovsky, E.; Chirik, P. J. Iron-Catalyzed Intermolecular [2π + 2π] Cycloaddition. *J. Am. Chem. Soc.* **2011**, *133* (23), 8858–8861.

(16) Cramer, H. H.; Duchemin, C.; Kovel, C. B.; Kim, J.; Pecoraro, M. V.; Chirik, P. J. Ligand Field Sensitive Spin Acceleration in the Iron-Catalyzed [2 + 2] Cycloaddition of Unactivated Alkenes and Dienes. *J. Am. Chem. Soc.* **2024**, *146* (14), 9947–9956.
(17) Abbott, L. J.; Hart, K. E.; Colina, C. M. Polymatic: A Generalized Simulated Polymerization Algorithm for Amorphous Polymers. *Theor. Chem. Acc.* **2013**, *132* (3), 1334.
(18) Patrone, P. N.; Dienstfrey, A.; Browning, A. R.; Tucker, S.; Christensen, S. Uncertainty Quantification in Molecular Dynamics Studies of the Glass Transition Temperature. *Polymer* **2016**, *87*, 246–259.
(19) Coleman, S. P.; Spearot, D. E.; Capolungo, L. Virtual Diffraction Analysis of Ni [0 1 0] Symmetric Tilt Grain Boundaries. *Model. Simul. Mater. Sci. Eng.* **2013**, *21* (5), 055020.
